# Bottom-up reconstruction of synthetic pyrenoids provides insights into the mechanisms and evolution of carbon concentration by EPYC1 proteins

**DOI:** 10.1038/s41477-026-02349-x

**Published:** 2026-08-14

**Authors:** A. M. Küffner, B. Pommerenke, L. Kley, J. Z. Y. Ng, S. Prinz, M. Tinzl-Zechner, L. Schulz, P. Claus, N. Paczia, T. Chotel, M. Klose, J. Zarzycki, G. K. A. Hochberg, T. J. Erb

**Affiliations:** 1 Research Group Sustainable Biocatalysis, Max Planck Institute for Multidisciplinary Sciences, Göttingen, Germany; 2 Department of Biochemistry & Synthetic Metabolism, Max Planck Institute for Terrestrial Microbiology, Marburg, Germany; 3 Phillips-University of Marburg, Marburg, Germany; 4 Central Electron Microscopy Facility, Max Planck Institute of Biophysics, Frankfurt, Germany; 5 Microbes-for-Climate (SYNMIKRO), Marburg, Germany; 6 Center for Synthetic Microbiology (SYNMIKRO), Marburg, Germany

**Keywords:** Microbiology, Phylogenetics, Cell biology

## Abstract

Membraneless organelles play essential roles in many cellular processes. In various photosynthetic organisms, they are a crucial part of CO_2_/carbon-concentrating mechanisms (CCMs) that increase photosynthetic productivity. One example is the pyrenoid in *Chlamydomonas reinhardtii*, a liquid-phase-separated organelle that localizes and improves CO_2_ fixation via the intrinsically disordered protein essential pyrenoid component 1 (EPYC1_CR_). Modern-day pyrenoids are complex structures with an elaborate cellular architecture and dozens of components, raising the question of how they could have developed from simpler condensates. Here we develop a bottom-up approach to study the function of EPYC1s and explore their sequence–function space across phylogenetic diversity and evolution. We demonstrate that extant and ancestral EPYC1 sequences induce phase separation of Rubisco into synthetic pyrenoids with functional CCMs. Surprisingly, these CCMs are mainly based on enhanced carboxylation rates (rather than increased specificity), offering new insights into the construction, function and evolution of natural and synthetic pyrenoids.

## Abstract (web summary)

This study shows that the structural protein essential pyrenoid component 1 (EPYC1) and the enzyme Rubisco are sufficient to assemble into pyrenoid-like structures that are functionally active and show a basic carbon-concentrating activity.

## Main

Membraneless organelles play key roles in organizing cellular processes in time and space across all domains of life^1–11^. In living cells, the roles of these liquid–liquid-phase-separated structures, also called condensates, include RNA transportation, concentration buffering and modulating metabolic networks at pivotal branch points^10,12,13^.

Condensates are also involved in increasing the efficiency of photosynthesis. In various green algae, red algae, diatoms, hornworts and endosymbiont algae (haptophytes and dinoflagellates), a condensate inside the chloroplast or red plastid—called the pyrenoid—functions as a core part of the CO_2_/carbon-concentrating mechanism (CCM)^7,14–16^. Pyrenoids are functionally conserved and evolutionarily convergent structures that enrich inorganic carbon inside photosynthetic cells^15–17^. They increase the local carbon dioxide concentration in close proximity to ribulose-1,5-bisphosphate carboxylase oxygenase (Rubisco), the CO_2_-fixing enzyme of the Calvin–Benson–Bassham cycle^18,19^. This local increase in CO_2_ promotes the carboxylation reaction of Rubisco and suppresses the reaction of the enzyme with oxygen that causes the wasteful process of photorespiration^20^.

**Fig. 1:**
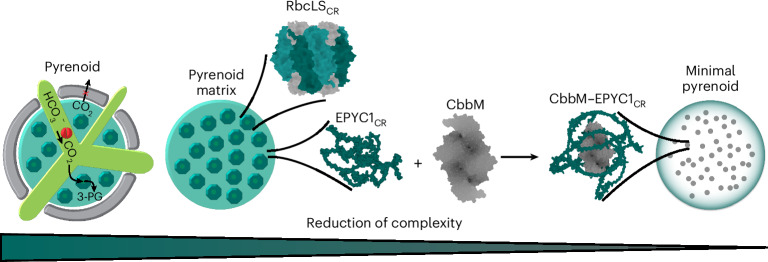
Reduction of pyrenoid complexity for in vitro investigation of the pyrenoid matrix catalytic mechanism. Our approach for reducing in vitro pyrenoid complexity to interrogate the fundamental catalytic function of EPYC1_CR_ in liquid-phase-separated condensates to then reproduce in the pyrenoid matrix reconstitution system consisting of RbcLS_CR_ and EPYC1_CR_.

The best-studied case of condensate-based CCMs is the pyrenoid in the green alga *Chlamydomonas reinhardtii*, which is composed of several components. The inner part, known as the pyrenoid matrix, contains almost all of the cell’s Rubisco. This matrix is formed through co-condensation of the protein essential pyrenoid component 1 (EPYC1_CR_, EPYC from *C. reinhardtii*), an intrinsically disordered protein (IDP), with canonical form I Rubisco, an enzyme complex that is composed of a catalytically active large subunit and an auxiliary small subunit (SSU)^21^. Matrix formation is essential for carbon concentration: EPYC1_CR_ deletion mutants show no pyrenoid formation and cannot survive at ambient CO_2_ concentrations^7,22^. Beyond this functional core, the CCM in *C. reinhardtii* contains additional elements (Fig. 1). The matrix itself is further surrounded by a starch sheath, which has been suggested to stabilize the pyrenoid and prevent CO_2_ leakage^8,23^. Additionally, thylakoid membrane tubules protrude into the pyrenoid and deliver inorganic carbon in the form of HCO_3_^−^ for carbon fixation^24^. Within these tubules, carbonic anhydrase converts HCO_3_^−^ into CO_2_, which diffuses into the matrix, where it serves as substrate for Rubisco and is incorporated into ribulose-1,5-bisphosphate (RuBP) to yield two molecules of 3-phosphoglycerate (3-PG).

However, it is still unclear how pyrenoids function at a molecular level and which minimal components would be required to reconstitute a functional CCM. This is even more interesting when thinking about the convergent evolution of pyrenoids in diverse clades (green algae, red algae, diatoms and hornworts) and the diverse Rubisco forms that are targeted to the pyrenoid, as well as the structural organization and complexity of pyrenoids between these groups of organisms^25,26^.

Recent work has delivered insights into the molecular basis of pyrenoid formation in *C. reinhardtii*. In vitro studies showed that EPYC1_CR_ interacts with the SSU (but not the large subunit) of form I Rubisco^9^ via short α-helical motifs to induce matrix formation^27^. However, whether EPYC1_CR_-based condensation directly affects Rubisco catalysis or whether the CO_2_/carbon-concentrating effect of the pyrenoid is rather caused by secondary effects (that is, substrate partitioning and/or delivery) is still unclear. In addition, what mechanisms could have driven the formation of simple condensates and what advantages such minimal condensates could have potentially provided have only been investigated on a theoretical basis^28^.

Here we combined synthetic biology and evolutionary biochemical approaches to study the mechanisms and capabilities of different IDP-containing proteins in condensate formation in vitro (Fig. 1). Using a reductionist approach, we demonstrate that EPYC1_CR_ homologues are sufficient to induce the formation of synthetic pyrenoids when fused to Rubisco. We further show that minimal pyrenoid formation is specific to EPYC1_CR_ homologues, while other (that is, non-pyrenoid-related) IDPs do not promote phase separation of Rubisco.

We further explored the sequence–function space of EPYC1s by testing their properties across the phylogenetic tree and used ancestral sequence reconstruction to assess ancestral EPYC1s. Extant as well as resurrected EPYC1s all form synthetic pyrenoids with functional CCMs that mainly enhance the carboxylation rate (rather than selectivity). Finally, we demonstrate that EPYC1-based condensates enhance carboxylation without substantially altering the catalytic parameters or structure of Rubisco, providing evidence that the basal CCM effect of EPYC1-based (synthetic) pyrenoids is rooted in mass action kinetics.

## Results

### EPYC1_CR_ causes the condensation of simple form II Rubisco

We first screened the capability of diverse IDPs for liquid–liquid phase separation (LLPS) of Rubisco. To that end, we selected the well-studied form II Rubisco from *Rhodospirillum rubrum* (CbbM) as a target. CbbM is a simple bacterial Rubisco that consists only of a large subunit, which exists as a homodimer and can be produced soluble at high protein concentrations. Although CbbM did not co-evolve with EPYC1_CR_ (or any other IDP), we reasoned that the enzyme provides a robust platform to study the effects of IDP fusions on Rubisco-based CO_2_ fixation.

**Fig. 2:**
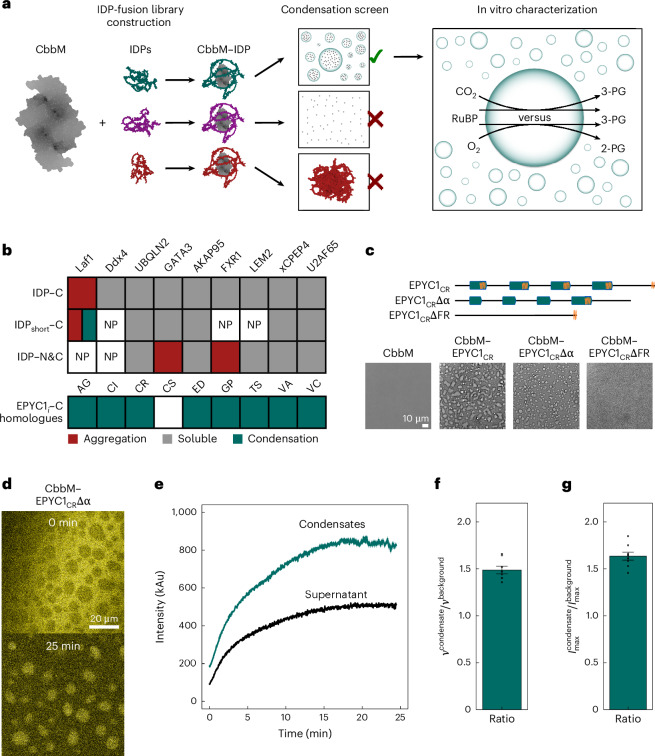
IDP-fusion screen to identify and characterize functional synthetic pyrenoids in vitro. **a**, General workflow. A library of IDPs^3,9,29–36^ was fused to CbbM. Each fusion protein was individually tested in a condensation screen for phase separation, no effect (that is, homogenous dispersal) or aggregation. Condensates were further subjected to carboxylation activity and specificity tests. **b**, Overview of the fusion protein library and condensation screen results. Full (or shortened) IDPs were fused to the C terminus (IDP–C and IDP_short_–C) or the N and C termini (IDP–N&C) of CbbM. Red indicates aggregation, grey indicates homogeneously dispersed protein, white indicates not producible (CbbM–Laf1N&C, CbbM–Ddx4N&C and CbbM–EPYC1_CS_) or not tested (CbbM–Ddx4_short_, CbbM–FXR1_short_ and CbbM–LEM2_short_) and green indicates condensate formation at concentrations of 1–20 μM. CbbM–Laf1_short_ undergoes LLPS when mixed with 1 μM GFP and shows aggregation at all other conditions tested. EPYC1 homologues are fused to the C terminus of CbbM and are further denoted as, for example, EPYC1_AG_, with AG referring to the homologue from *A. gubernucalifera*. Each condensation screen was tested three independent times. **c**, Architecture and behaviour of different EPYC1 variants from *C. reinhardtii*. The chloroplast-targeting peptide was removed prior to analysis and production (amino acids 1–27 of the native protein; Supplementary Table 1). Green arrows indicate repeat domains, and orange helices show SSU-recognition domains. CR, full-length IDP; Δα, SSU-recognition domain deletion variant (deletion of amino acids 37–46, 99–108, 159–168 and 286–290); ΔFR, repetition domain deletion variant (deletion of amino acids 22–46, 80–108, 140–168 and 200–229). The mutant with full deletion of all SSU-recognition domains could not be purified. The light microscopy pictures show homogeneously dispersed CbbM and CbbM–EPYC1_CR_ΔFR, while CbbM–EPYC1_CR_ and CbbM–EPYC1_CR_Δα form condensates in 50 mM Tris (pH 7.5 and 8.0), 1 mM MgCl_2_, 5 mM NaHCO_3_, 0.1 mg ml^−1^ CA and 0.3 mM RuBP. The condensate formation test was repeated three times. **d**, Fluorescence microscopy pictures from the resorufin-coupled activity assay (Supplementary Fig. 8) inside and outside of condensates at 0 min and at 25 min from one repetition. **e**, Fluorescence formation inside and outside of condensates from **d**. The original signal was scaled by 1,000 for readability; kAu, kilo artbitrary units. **f**, Ratio of initial fluorescence formation rates between condensate inside and outside, represented by the centre of the error bars. The data points represent eight technical replicates. The error bars denote standard error. **g**, Ratio of maximum fluorescence levels between condensate inside and outside, as in **f**. The data points represent eight technical replicates. The error bars represent standard error. *ν*^condensate^ is the initial rate of fluorescence evolution inside the condensate; *ν*^background^ is the initial rate of fluorescence evolution in the supernatant; $I_{\rm{max}}^{\rm{condensate}}$ is the maximum intensity measured inside the condensates; and $I_{\rm{max}}^{\rm{background}}$ is the maximum intensity measured in the supernatant. Source data

Using CbbM as a target, we fused a library of IDPs^3,9,29–36^ to the protein’s carboxy terminus and to its C and amino termini (Fig. 2a,b and Supplementary Table 1). This library contained EPYC1 from *C. reinhardtii* (EPYC1_CR_), as well as nine other IDPs that had previously shown condensate formation as individual proteins or when fused to other target enzymes. When tested between concentrations of 1 and 20 µM, only EPYC1_CR_ initiated functional and stable condensates with its non-natural target CbbM (Fig. 2b and Supplementary Figs. 1 and 2). All other IDPs—except for a few IDPs that aggregated—showed no phase-separating effect on CbbM (Supplementary Figs. 2 and 3) but seemed also not to dramatically alter the catalytic parameters of the enzyme (Supplementary Fig. 4). The fact that only EPYC1_CR_ (and its homologues; Fig. 2b) showed LLPS when fused to CbbM indicates that specific sequences are required to generate pyrenoid-like condensates in vitro. This is also supported by a distinct sequence composition of EPYC1 variants compared with other IDPs (Supplementary Figs. 5 and 6).

We also tested truncated versions of EPYC1_CR_. The deletion of the Rubisco SSU recognition motif (EPYC1_CR_Δα) did not change condensate formation, which is in line with the fact that form II Rubiscos do not possess a SSU that could interact with the SSU recognition motif (Fig. 2c). However, the deletion of the complete repeat domain of EPYC1_CR_ (EPYC1_CR_ΔFR) completely abolished condensate formation of CbbM, indicating the importance of these domains for phase separation (Fig. 2c). Interestingly, EPYC1_CR_ and EPYC1_CR_Δα—when not fused to CbbM—were capable of undergoing homotypic LLPS, albeit only at minimum tenfold higher concentrations (>10 µM; Supplementary Fig. 7) than their CbbM fusion analogues (~1 µM; Fig. 1b). This decrease in critical solution concentration of fusion proteins could be the result of ‘extension effects’^37–41^, more specifically the fact that CbbM forms a homodimer in solution, which increases the interaction frequency between IDPs and consequently increases phase separation.

In summary, our results show that condensate formation with EPYC1 is not restricted to form I Rubiscos but is also in principle possible with simpler form II Rubiscos. Notably, form II Rubiscos require EPYC1 for LLPS and cannot form condensates with other IDPs. In the following, we used these CbbM–EPYC1 fusions as orthogonal model systems to study the function of EPYC1 in CO_2_/carbon concentration.

### CbbM–EPYC1-based condensates are functionally active

We next tested whether our synthetic condensates were functionally active and showed CCM properties. To assess the activity of CbbM within the condensates, we developed a coupled fluorescence assay (Supplementary Fig. 8). This assay catalyses the oxidation of 3-PG to acetyl phosphate and hydrogen peroxide, which can be detected using horseradish peroxidase and resorufin. We used confocal microscopy to monitor the fluorescence signal of CbbM–EPYC1_CR_Δα inside and outside of condensates over time (Fig. 2d–g and Supplementary Video 1). In the absence of RuBP, the substrate of Rubisco, we observed only a weak background signal outside of the condensates. Upon the addition of RuBP, we detected fluorescence formation inside and outside of the condensates. The rate of fluorescence formation was increased inside the condensate, which is in line with the majority of protein being located within condensates, as confirmed by analytical size exclusion chromatography (SEC) (80–97%; Supplementary Fig. 9). These findings show that our condensates were enzymatically active and formed minimal ‘synthetic’ pyrenoids that could (partially) emulate the function of natural pyrenoids (that is, increased CO_2_ fixation). Interestingly, the resorufin intensity did not equilibrate between condensate and continuous phase after 25 min, despite resorufin not partitioning preferentially into the condensates (Supplementary Fig. 8).

**Fig. 3:**
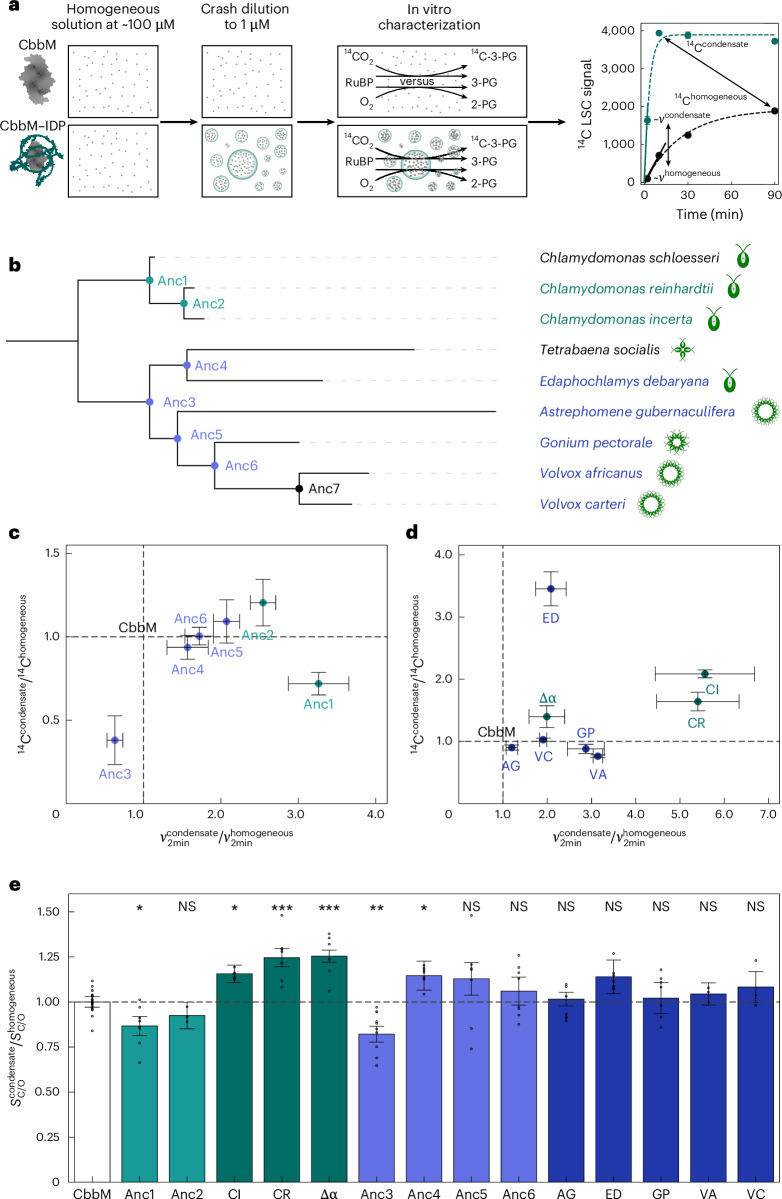
Characterization of EPYC1 homologues across the phylogenetic and evolutionary tree. **a**, General workflow. CbbM and CbbM–IDP fusion proteins were prepared at ~100 µM concentrations (yielding homogenously dispersed protein stocks). Crash dilution to a final concentration of 1 µM was used to induce phase separation of the CbbM–IDP fusions. Carboxylation assays were performed side-by-side at 25 °C using ^14^CO_2_, yielding time-curve data from liquid scintillation counter (LSC) measurements of homogenous CbbM (black lines) and phase-separated CbbM–IDP (green lines). The arrows indicate the computation of initial carboxylation rate ratios and maximum ^14^C incorporated ratios. **b**, Phylogeny of EPYC1_CR_ and its nine known homologues as inferred by maximum likelihood. The tree was rooted at the oldest node following the species relationships of the Chlamydomonadales order^42^. Teal and light purple show cloned, produced and stable EPYC1 ancestors; green and blue indicate cloned, produced and stable extant EPYC1 homologues. EPYC1_CS_ could not be subcloned; EPYC1_TS_ forms condensates in pure buffer, but these dissolve under reaction conditions; Anc7 showed little protein production and no phase separation. **c**, Maximum ^14^C measured ratios versus initial rate ratios for the produced fusion proteins of CbbM and EPYC1 ancestors represented by the centres of the error bars. Each ratio was computed from a ^14^C time curve of CbbM–IDP in condensate-forming conditions compared to CbbM only. Ancestral fusion proteins from the *Chlamydomonas* family are shown in teal and ancestral fusion proteins from the *Volvox* family in light purple. The dotted lines illustrate homogeneous CbbM. The reactions were conducted in non-saturating conditions at pH 7.5–8.0, 5 mM NaHCO_3_ or NH_4_HCO_3_, 0.1 mg ml^−1^ CA, 0.3 mM RuBP, 25 °C, 300 rpm and at atmospheric conditions. For each ratio, three independent replicates for homogeneous CbbM and for phase-separated CbbM–IDP were used. The variance of homogeneous CbbM was estimated from all CbbM measurements. Variance was computed using empirical error propagation. The error bars indicate standard errors. **d**, Maximum ^14^C measured ratios versus initial rate ratios for the produced fusion proteins of CbbM and EPYC homologues represented by the centres of the error bars. For each ratio, three independent replicates for homogeneous CbbM and for phase-separated CbbM–IDP were used. For EPYC1CR, two datasets were combined with two and three replicates, respectively. For EPYCED, two datasets of three replicates each were combined. The variance of homogeneous CbbM was estimated from all CbbM measurements. Variance was computed using empirical error propagation. The error bars indicate standard errors. **e**, Ratios of specificity (*S*_C/O_) between homogeneous CbbM and phase-separated CbbM–IDP measured in 4,991-ppm CO_2_ in O_2_ represented by the centres of the error bars. In each specificity assay, CbbM was measured alongside the fusion protein to control for daily variations. The data points represent 29, 8, 11, 4, 8, 7, 8, 8, 6, 7, 6, 7, 3, 3 and 6 independent replicates. Errors were propagated to the ratios using empirical error propagation. The error bars indicate standard errors. *P* values were computed using two-sided *t*-tests testing for differences from CbbM (*P < 0.05; **P < 0.01; ***P < 0.001; NS, not significant). *P* values from left to right are as follows: 0.0382, 0.3697, 0.0265, 0.0005, 0.0005, 0.0021, 0.0405, 0.0866, 0.3815, 0.7903, 0.0736, 0.7675, 0.6354, 0.3826). *ν*^condensate^ is the initial rate of the whole sample/system with condensates; *ν*^homogeneous^ is the initial rate of the whole sample/system without condensates; ^14^C^condensate^ is the maximum ^14^C measured in the whole sample/system with condensates; ^14^C^homogeneous^ is the maximum ^14^C measured in the whole sample/system without condensates; ${\upnu}_{2{\rm{min}}}^{\rm{condensate}}$ is the initial rate of the whole sample/system with condensates, utilizing the first two minutes of the time curve data; ${\upnu}_{2{\rm{min}}}^{\rm{homogeneous}}$ is the initial rate of the whole sample/system without condensates, utilizing the first two minutes of the time curve data; $S_{\rm{C/O}}^{\rm{condensate}}$ is the specificity of carboxylation over oxygenation (C/O) of the whole sample/system with condensates; $S_{\rm{C/O}}^{\rm{homogeneous}}$ is the specificity of carboxylation over oxygenation (C/O) of the whole sample/system without condensates. CI, *Chlamydomonas incerta*; CR, *Chlamydomonas reinhardtii*; CS, *Chlamydomonas schloesseri*; ED, *Edaphochlamys debaryana*; GP, *Gonium pectorale*; TS, *Tetrabaena socialis*; VA, *Volvox africanus*; and VC, *Volvox carteri*. Source data

**Fig. 4:**
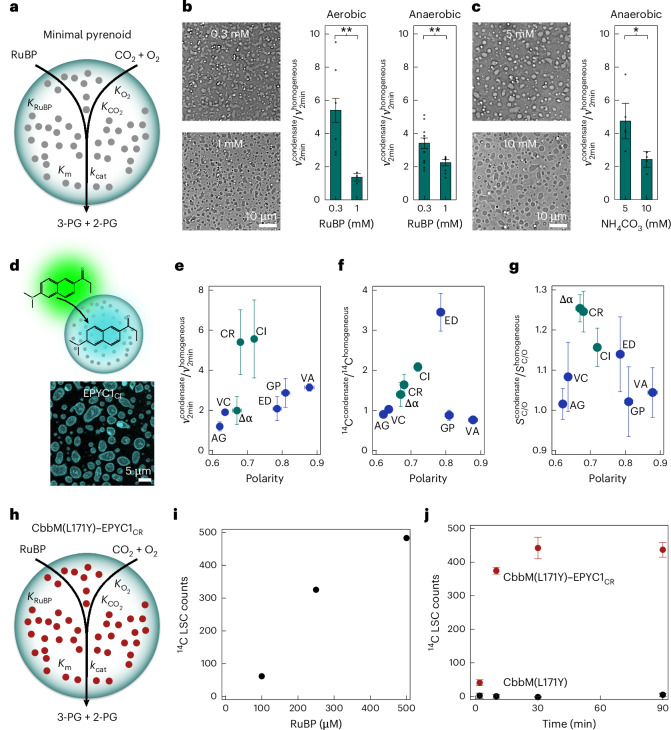
Kinetic analysis and material properties of synthetic pyrenoids. **a**, Parameters affected by partitioning of enzymes and substrates into the synthetic pyrenoids. **b**, Ratio of initial carboxylation rates between CbbM and CbbM–EPYC1_CR_ at 0.3 and 1 mM RuBP in aerobic and anaerobic conditions at pH 8.0, 0 mM NaCl, 1 mM MgCl_2_, 50 mM Tris and 5 mM NH_4_HCO_3_ represented by the centres of the error bars. Ratios were determined from 6 and 3 (aerobic) and 12 and 9 (anaerobic) independent replicates. The error bars denote standard errors empirically propagated to the ratio. *P* values were computed using two-sided *t*-tests testing for differences between high and low RuBP concentrations (**P < 0.01; *P* = 0.0061 and 0.0020). **c**, Ratio of initial carboxylation rates between CbbM and CbbM–EPYC1_CR_ at 5 mM and 10 mM NH_4_HCO_3_ in anaerobic conditions at pH 8.0, 0 mM NaCl, 1 mM MgCl_2_, 50 mM Tris and 0.3 mM RuBP represented by the centres of the error bars. Ratios were determined from five independent replicates each. The error bars denote standard errors empirically propagated to the ratio. *P* values were computed using two-sided *t*-tests testing for differences between high and low NH_4_HCO_3_ concentrations (*P < 0.05; *P* = 0.0344). Microscopy analysis showed no morphological difference between the different conditions used in **b** and **c** (Supplementary Fig. 11). **d**, Representative confocal microscopy image of condensates formed by CbbM–EPYC1_CI_ with partitioned solvatochromatic dye PRODAN (~1 mM, pH 7.5, 50 mM Tris) to quantify the polarity of condensates^50^ from one out of six independent repetitions. **e**, Ratios of initial carboxylation rates of different EPYC1 condensates plotted against condensate polarity. Polarity was measured from a minimum of 5 condensates in each replicate of AG, CI, ED, Δα, GP, VA, VC and CR; the number of independent replicates in each condition was 5, 6, 3, 3, 3, 3, 3 and 6, respectively. The error bars denote standard errors. **f**, Maximum ^14^C incorporated ratios from Fig. 3c,d plotted against condensate polarity. The error bars denote standard errors. **g**, Specificity ratios plotted against condensate polarity. The error bars denote standard errors. **h**, Parameters potentially affected by condensation in a CbbM mutant with substantially worse catalytic parameters (CbbM(L171Y))^53^. **i**, ^14^C incorporated over two hours in anaerobic conditions with 1 µM CbbM(L171Y), pH 7.5, 5 mM ^14^CO_3_^2−^, 1 mM MgCl_2_, 0.1 mg ml^−1^ CA, 50 mM Tris and RuBP concentrations of 100, 250 and 500 µM. **j**, Aerobic time-curve data for pyrenoid mimics formed by CbbM(L171Y)–EPYC1_CR_ and homogeneous CbbM(L171Y) in the same conditions as in **i** apart from using 300 µM RuBP. The error bars denote standard errors. *K*_m_ is the Michaelis Menten constant of Rubisco; *K*_RuBP_ is the partition coefficient of RuBP into the condensates; ${K_{{\rm{CO}}_{2}}}$ is the partition coefficient of CO_2_ into the condensates; and ${{K_{{\rm{O}}_{2}}}}$ is the partition coefficient of O_2_ into the condensates. Source data

To quantify the catalytic properties of EPYC1-condensed CbbM compared with homogeneous CbbM, we used radioisotope tracing with ^14^CO_2_. Because CCMs are most pronounced at sub-saturating substrate conditions (and our condensates showed limited stability at high substrate concentrations), we performed our assays at non-fully saturating substrate concentrations—that is, 5 mM H^14^CO_3_^−^ (≈0.08–0.25 mM CO_2_) and 0.3 mM RuBP—and followed [^14^C]-3-PG formation over time (Fig. 3a). We determined carboxylation rates over the first two minutes and quantified maximum total [^14^C]-3-PG levels as a proxy for carboxylation yield. In these experiments, phase-separated CbbM–EPYC1_CR_ showed carboxylation rates of about 4 CO_2_ s^−1^ per active site and carboxylation yields of about 0.3 mM [^14^C]-3-PG (≈50% of RuBP). In strong contrast, the carboxylation rates of homogeneous (that is, non-phase-separated) CbbM were substantially lower (fourfold to fivefold) at 0.7–1 CO_2_ s^−1^ per active site. Additionally, homogeneous CbbM formed only 0.2 mM [^14^C]-3-PG product (≈33% of RuBP). Overall, these data indicate that CbbM showed lower specific activities and catalysed a substantial fraction of oxygenation when not phase separated into a synthetic pyrenoid, which we subsequently confirmed experimentally (Figs. 3 and 4).

### Condensate-forming properties are conserved across the EPYC1 phylogeny

We next sought to investigate the potential of EPYC1 homologues to form synthetic pyrenoids. So far, nine EPYC1 homologues are known from green algae, all from the order of Chlamydomonadales. In the following, we use index labelling to indicate the originating species for these nine homologues (for example, EPYC1_AG_ for the homologue from *Astrephomene gubernaculifera*). Of these nine sequences, we could successfully produce eight as fusion proteins with CbbM. All eight homologues formed condensates (Fig. 2b and Supplementary Fig. 2), showing that the basic condensate-forming property is conserved across these homologues, despite a sequence conservation as low as 57% between the different variants (Supplementary Tables 3 and 4).

To study the evolution of these sequences, we created a phylogeny of the nine EPYC1-containing species in an unrooted tree (Supplementary Fig. 10), which we subsequently rooted using the known species relationships of Chlamydomonadales^42^ (Fig. 3b). In our phylogeny, two main families diverge within Chlamydomonadales: the *Volvox* family and the *Chlamydomonas* family. Within the *Chlamydomonas* branch, all species are strictly unicellular freshwater organisms, whereas almost all species of the *Volvox* branch inhabit fresh and saltwater and form multicellular spheroidal colonies, except the unicellular species *Edaphochlamys debaryana*^43–46^. We used ancestral sequence reconstruction to infer ancestral EPYC1 sequences at internal nodes along the phylogenetic tree (Anc1–7) and expressed them as C-terminal fusions to CbbM. Except for Anc7, which exhibited minimal protein production and condensate formation, all ancestral sequences resulted in stable proteins that induced phase separation of CbbM. These data suggest that the condensate-forming property is not an acquired trait of modern EPYC1 homologues but was already prevalent in ancestral EPYC1 sequences, including the last common ancestors of the *Chlamydomonas* (Anc1) and the *Volvox* family (Anc3).

### Extant and ancestral EPYC1s mainly enhance carboxylation rates in condensates

In the following, we tested all extant and ancestral sequences with respect to carboxylation rate enhancement and maximum amount of 3-PG produced using the radioisotope assay described above. All ancestors except Anc3 exhibited twofold to threefold higher rates than homogeneous CbbM at sub-saturating conditions, suggesting that rate enhancement is an ancestral trait (Fig. 3c–e). With respect to maximum 3-PG formation (as a proxy for specificity), the picture was more complex. Compared with homogeneous CbbM, the last common ancestors (Anc1 and Anc3) showed decreased 3-PG production, while 3-PG production was not substantially different in all other ancestors (and only slightly enhanced in Anc2). These results indicate that ancestral EPYC1 homologues might not have conferred specificity advantages, although we cannot exclude the possibility that the picture might change for systems consisting of ancestral EPYC1s and their cognate Rubiscos.

Expanding the investigation to extant homologues of EPYC1 (Fig. 3d,e) allowed us to infer general trends in rate enhancement along the evolutionary trajectories of the *Chlamydomonas* and *Volvox* families. In the *Chlamydomonas* branch, Anc1 and Anc2 showed twofold to threefold enhanced carboxylation rates in condensates. This effect was increased between fivefold and sixfold for the extant EPYC1 homologues of *C. reinhardtii* and *C. incerta* (Fig. 3d). In the *Volvox* branch, we observed a similar pattern. While Anc3 showed decreased carboxylation rates, we observed twofold to threefold enhanced rates for ancestors Anc4–6 of the *Volvox* family (except for *A. gubernaculifera*) in condensates.

With respect to the maximum amount of 3-PG produced, Anc2 in the *Chlamydomonas* branch showed an improved ratio of ~1.2-fold. This ratio was elevated to ~1.6- and 2-fold in extant *Chlamydomonas* homologues. In the *Volvox* branch, only the extant homologue from *E. debaryana* showed ~3-fold increased maximum production of 3-PG (Fig. 3e)^43,46^. To quantify specificity more precisely, we employed an alternative assay to the maximum 3-PG formation readout, in which we used defined gas mixtures and determined the formation of glycerate and glycolate (Fig. 3f and Methods). This assay reproduced the overall trend in specificity enhancements observed with the maximum 3-PG formation assay but showed less pronounced fold-changes. This difference in absolute numbers is probably caused by the different set-ups of the two assays—specifically, the supply of inorganic carbon, which is provided in the form of bicarbonate in the maximum 3-PG formation assay and as a CO_2_/O_2_ gas mixture in the specificity assay.

In summary, our data show that ancestral EPYC1 sequences possess phase-separating capabilities down to the last common ancestors and consistently enhance carboxylation rates in condensates. In contrast, the signal for specificity-conferring properties encoded in EPYC1 sequences is rather weak and less clear.

### Substrate partitioning and mass action effects explain the CCM of CbbM–EPYC1 condensates

Having demonstrated that condensed CbbM–EPYC1 fusions confer a functional CCM in vitro, we sought to explore the underlying mechanisms. We considered two different modes of action. In the first model, the condensate’s chemical environment (for example, polarity and pH) directly affects the enzyme itself^47^ (for example, through conformational shifts or allosteric alterations), which changes the enzyme’s kinetics, similar to the influence of organic solvents on the lid behaviour and catalytic effects of esterases/lipases^48^. In the alternative model, physicochemical effects, such as substrate partitioning, explain global changes in catalytic properties of the system (Figs. 3c,d and 4a–d). In this model, differences in the chemical environment (for example, different solvation of substrates) cause differences in reactant concentrations inside condensates, resulting in multiphase reaction systems with mass action effects that affect catalytic rate and specificity.

To distinguish between these two hypotheses, we used the fact that mass action kinetic effects typically decrease with increasing substrate concentrations^49^. This reduction is due not to increased specific catalytic activity (*v*_max_) but to increased active site saturation at increasing concentrations, and thus convergence of catalytic rates (*k*_cat_ E limit). Indeed, increasing RuBP concentrations (under aerobic and anaerobic conditions; Fig. 4a,b) as well as increasing CO_2_ concentrations (under anaerobic conditions; Fig. 4c) decreased the differences in rate enhancement between phase-separated and homogenous CbbM, suggesting a mass-action-based CCM in our synthetic pyrenoids.

To independently test both hypotheses, we used a model-based approach that fit mass action kinetic models based on generalized Michaelis–Menten kinetics^49^ to our experimental data at 0.3 and 1 mM RuBP (Supplementary Figs. 11 and 12). To test the substrate partitioning hypothesis, we varied the partitioning of RuBP, CO_2_ and O_2_ into the condensates while keeping the kinetic parameters of CbbM constant for both phase-separated and homogeneous conditions. With these assumptions, our experimental data were modelled best by a differential ratio of CO_2_/O_2_ partitioning between 3.6 and 8.7 (at 0.3 and 1 mM RuBP; Supplementary Table 2), which is well in line with previously reported partitioning coefficients^50,51^.

We also tested the alternative hypothesis that condensate formation directly affects the kinetic properties of CbbM by varying *K*_m_(CO_2_) and *K*_m_(O_2_) for phase-separated conditions. Varying those parameters, the experimental data could be explained only by a 6-fold decrease in *K*_m_(CO_2_) at 0.3 mM RuBP (1.5-fold decrease at 1 mM RuBP) and a 14-fold increase in *K*_m_(O_2_) at 0.3 RuBP (2.6-fold increase at 1 mM RuBP) inside condensates. This would translate into a dramatic change in CbbM specificity of one order of magnitude (at 1 mM RuBP) and up to three orders of magnitude (at 0.3 mM RuBP) within condensates. Such a drastic change of enzymatic parameters through condensate formation, however, is unreported to date, and thus rather unlikely compared with the substrate partitioning hypothesis.

### Material properties of condensates point to solvation effects

To further elucidate the physicochemical mechanisms of the CCM in CbbM–EPYC1s, we investigated the material properties of our condensates. We reasoned that the condensates might exhibit different polarity, internal pH and/or carbonic anhydrase partitioning. We therefore used a previously developed confocal-microscopy-based polarity assay^50^ to measure polarity across synthetic pyrenoids (Fig. 4d and Supplementary Fig. 13). Plotting polarity against the different Rubisco parameters (carboxylation rate, 3-PG formation and specificity) showed a weak optimum for rate enhancements around a polarity of 0.7, and more pronounced optima for 3-PG formation and specificity at 0.65 and 0.8 polarity, respectively (Fig. 4e–g). This correlation between polarity and specificity is reminiscent of the Sabatier^52^ principle observed in heterogeneous catalysis, where adsorption differences of substrates and products on the catalyst determine catalytic performance in form of a sharp, volcano-plot-like optimum, suggesting a similar Sabatier-like behaviour of condensates (that is, differential absorption of CO_2_/HCO_3_^2−^ and/or O_2_).

We also tested whether the catalytic effects correlate with internal condensate pH (for example, resulting in a different CO_2_/CO_3_ equilibrium). To this end, we employed the fluorogenic pH-sensor dye SNARF-1 (ref. ^47^). However, we did not observe a correlation between internal condensate pH and rate enhancements or 3-PG formation (Supplementary Fig. 14). Similarly, carbonic anhydrase partitioning, which we assessed by fluorescence measurements inside and outside of condensates, also did not correlate with the kinetic data (Supplementary Fig. 15). This led us to conclude that the solvation property (that is, polarity) is probably the most relevant factor in the (proposed) substrate partitioning of CbbM–EPYC1 condensates.

Noting that substrate partitioning in condensates is particularly prone to enhancing the activity of catalytically poor enzymes (Fig. 4h), we tested a recently reported CbbM variant (CbbM(L171Y)) that showed less than 0.2% carboxylation activity compared with wild-type CbbM^53^. Importantly, CbbM(L171Y) carboxylated RuBP exclusively under anaerobic conditions (Fig. 4i) and did not show carboxylation activity under aerobic conditions (Fig. 4j). However, when targeted into a condensate by fusion to EPYC1_CR_, CbbM(L171Y)–EPYC1_CR_ also performed carboxylation under aerobic conditions, increasing carboxylation product formation by more than one order of magnitude compared with homogeneous CbbM(L171Y). Together, these data not only support the substrate partitioning hypothesis but also showcase how condensates could have promoted the (early) evolution of enzymes by increasing proto-catalytic activities.

### Structural analysis shows no differences between CbbM and condensed CbbM–EPYC1_CR_Δα

**Fig. 5:**
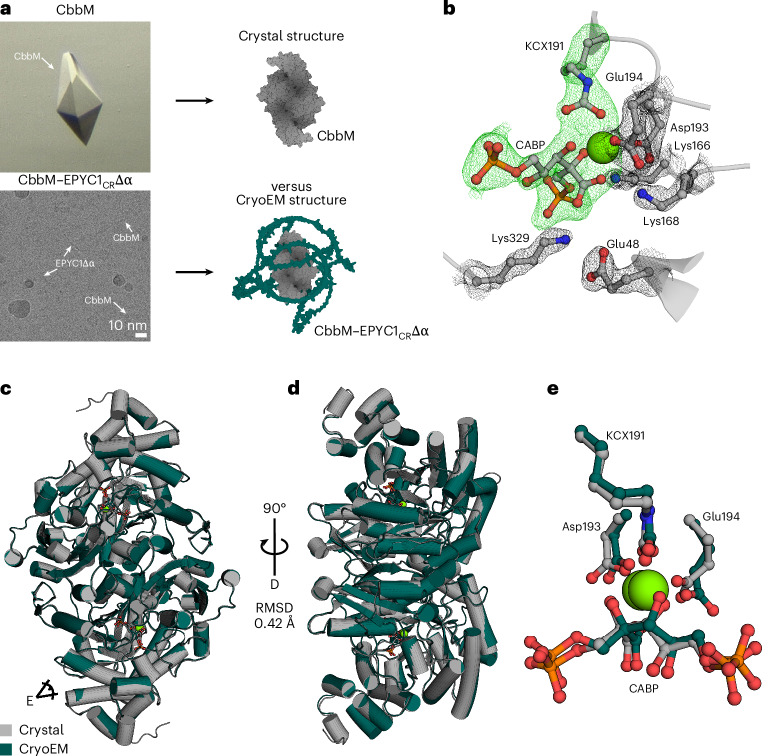
Structural analysis of crystallized and phase-separated CbbM. **a**, General workflow for structural comparison of crystallized CbbM and phase-separated CbbM–EPYC1_CR_Δα. A crystal structure of CbbM was compared with a cryoEM structure of CbbM–EPYC1_CR_Δα in the condensates. CbbM was crystallized via vapour diffusion in a closed conformer upon binding of CABP. CryoEM images of phase-separated CbbM–EPYC1_CR_Δα were recorded in condensate-forming conditions at the edges of the condensates. An image of a picked crystal and a representative micrograph out of 7,116 used micrographs are displayed. **b**, Active site of crystallized CbbM at 2.3 Å showing a simulated annealing omit map (Fo–Fc) for KCX, CABP and Mg in green at *σ* = 3 and the electron density map (2Fo–Fc) for active site residues in black at *σ* = 2 within a radius of 1.6 Å of the displayed selected atoms. **c**, Crystal structure of CbbM (grey) superimposed with the cryoEM structure of CbbM–EPYC1_CR_Δα (at 2.9 Å resolution, green). **d**,**e**, Superimposed model from **c** rotated by 90° (**d**) and zoom on the overlay of the inhibitor CABP, the carbamylated lysine 191 (KCX) and the Mg^2+^ between the crystal structure (grey) and the cryoEM structure (green) of CbbM (**e**).

Finally, we investigated whether condensate formation would cause any structural and/or allosteric effects on CbbM. We first solved the crystal structure of homogenous CbbM (without EPYC1_CR_Δα) in the presence of the substrate analogue 2-carboxyarabinitol-1,5-bisphophate (CABP) at a resolution of 2.3 Å (Fig. 5a). We then aimed at obtaining the structure of CbbM from condensates through single-particle analysis from cryogenic transmission electron microscopy (cryoEM) images of phase-separated CbbM in the presence of CABP. To that end, we used the CbbM–EPYC1_CR_Δα construct, which lacks most of the SSU-binding domains (Fig. 2) but mostly retains the catalytic properties of full-length EPYC1_CR_ (Fig. 3 and Supplementary Fig. 16).

To analyse the structure of condensed CbbM, we formed condensates on the carbon grid and collected EM images at the edges of condensates, where the density and thickness of the protein layer still allowed for single-particle analysis. This approach allowed us to solve the structure of the complete, phase-separated CbbM domain at a resolution of 2.9 Å. Critically, we exclusively detected dimeric particles and no higher-order oligomers. These data are fully in line with mass photometer data of homogenous CbbM and homogenous CbbM–EPYC1_CR_Δα (in non-condensate-forming buffer) that both showed a dimeric state of the enzyme (Supplementary Fig. 17), indicating that the CbbM domain formed a native complex in the condensate. Moreover, the phase-condensed CbbM cryoEM structure overlapped very well with the crystal structure of CbbM (Fig. 5b–e; root mean square deviation of 0.42 Å over 6,306 atoms), with almost perfect alignment of their respective active sites, indicating only minimal structural changes between condensed and homogenous CbbM. The only notable difference was a slight twist in active site residue Glu194 and the C5 phosphate group of CABP (Fig. 5e), which was probably due to a lower resolution of the condensate structure in combination with the best-fit procedure and/or radiation damage from cryoEM.

Besides dimeric CbbM particles, we also observed long, winding rods that we suspected to represent EPYC1_CR_Δα proteins. Although we were unable to reconstruct those particles at high resolution, we could obtain 2D classes of these structures. They resembled one to several EPYC1_CR_Δα proteins in a bundled shape that we were able to reconstruct to resolutions of approximately 12 Å (Supplementary Fig. 18). Taken together, these data suggest that the EPYC1_CR_Δα proteins bundled CbbM into the condensate, in which the CbbM domain retained its native conformation. In summary, our structural analysis shows no evidence that CbbM was allosterically modified in the synthetic pyrenoid. Together with the kinetic data, we argue that the observed CCM through EPYC1 is mainly caused by mass action kinetics and not by an altered structural state of the enzyme.

### The native pyrenoid matrix shows CO_2_-concentration-dependent carboxylation enhancement

**Fig. 6:**
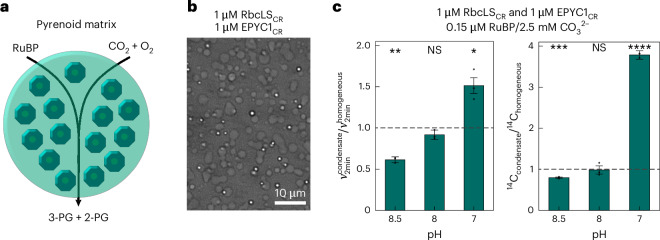
In vitro reconstitution of the pyrenoid matrix shows CO_2_-concentration-dependent carboxylation enhancement. **a**, The pyrenoid matrix reconstituted in vitro consisting of *C. reinhardtii* Rubisco (RbcLS_CR_) and EPYC1_CR_. **b**, Bright-field microscopy image showing condensates formed by 1 µM RbcLS_CR_ and 1 µM EPYC1_CR_ at pH 8.0, 1 mM NH_4_HCO_3_, 0.15 mM RuBP, 0.1 mg ml^−1^ CA, 1 mM MgCl_2_ and 50 mM Tris from one of four independent condensation tests. **c**, Initial rate ratios (left) as shown in Fig. 3 for the RbcLS_CR_ /EPYC1_CR_ system at 1 µM each, 1 mM NH_4_HCO_3_, 0.15 mM RuBP, 0.1 mg ml^−1^ CA, 1 mM MgCl_2_ and 50 mM Tris at different pH values represented by the centres of the error bars. Ratios were calculated from time-point data as in Fig. 3. Experiments at pH 7 were conducted in closed vials using sampling in contrast to having individual vials for each time point to counteract the quick degassing of ^14^CO_2_. pH 8.5 corresponds to 5.5, pH 8 to 17.4 and pH 7 to 138 µM dissolved CO_2_. Maximum ^14^C measured ratios (right) at the same conditions as on the left are represented by the centres of the error bars. The data points represent three independent replicates. Errors were propagated using empirical error propagation to the ratios. The error bars indicate standard errors. *P* values were computed using two-sided *t*-tests testing for differences from RbcLS_CR_ (*P < 0.05; **P < 0.01; ***P < 0.001; ****P < 0.0001). Left plot: *P* = 0.0015, 0.3656, 0.0190; right plot: *P* = 0.0003, 0.8917, 0.0000). Source data

While our CbbM–EPYC1 fusion approach allowed us to study the function of EPYC1s in a synthetic set-up, we wondered how these results would translate to the natural pyrenoid system. We thus purified native *C. reinhardtii* Rubisco RbcLS_CR_ as well as heterologously expressed EPYC1_CR_ (Fig. 6a). Analogous to our fusion proteins, the native system also formed condensates (Fig. 6b), which we tested for CCM activity at different pHs (Fig. 6c), substrate concentrations (Supplementary Fig. 19) and RbcLS_CR_:EPYC1_CR_ ratios (Supplementary Fig. 19). In these experiments, we observed an enhanced CCM (that is, 1.5-fold increased activity and 4-fold increased total carboxylation) at pH 7 and low substrate concentrations (that is, 50% RuBP and 20% CO_3_^2−^ compared with the fusion system; Fig. 6b). While the exact experimental conditions slightly differed from the set-up of the fusion system, these experiments confirmed that—just as for the fusion system—EPYC1_CR_-based condensation is sufficient for conferring a CCM, supporting the concept that even the most minimal pyrenoids are in principle capable of improving carbon fixation.

## Discussion

Understanding the molecular mechanisms of the CCM by pyrenoids is a fundamental challenge, which has been addressed mainly by in vivo studies in a top-down fashion. Here we used a reductionist, bottom-up approach to study pyrenoid formation in vitro from a mechanistic, evolutionary and structural point of view. Using a simple proteobacterial form II Rubisco (CbbM), we show that fusion with modern-day EPYC1 sequences is sufficient to induce phase separation of CbbM into synthetic pyrenoids, and that some of these condensates possess CO_2_/carbon-concentrating properties. The successful fusion approach with CbbM suggests that EPYC1 proteins already encode basic properties for enhanced carboxylation rates and in certain cases even more specific CO_2_ fixation. Yet, we note that our observation does not preclude the possibility that other proteins could further enhance CO_2_/carbon concentration in natural pyrenoids. While we could fully recapitulate the minimal pyrenoid CO_2_/carbon-concentrating effect in the pyrenoid matrix reconstitution system, a more complex and dynamic organization might be required for optimal function of pyrenoids in vivo.

Although modern-day pyrenoids are more complex structures, our results provide evidence that for the initial evolution of pyrenoids, a single phase-separating protein could already provide a benefit and was probably sufficient. This allows us to propose an evolutionary path from minimal pyrenoids to more complex structures, which are prevalent in green algae today. Considering the relatively high levels of CO_2_ (and low levels of O_2_) during early Earth history, it is unlikely that selection initially acted on enhanced specificity in EPYC1 sequences. This is in line with our data on ancestral (and extant) EPYC1 homologues that consistently show carboxylation rate enhancements, indicating that this feature of EPYC1 is an ancient trait. Following this logic, the evolution of enhanced specificity (which is more relevant today) would appear to be a more recent phenomenon, probably driven by declining environmental CO_2_ concentrations and increasing O_2_ concentrations.

Through structural studies and theoretical and material analysis, we propose that the CCM of EPYC1 in our synthetic pyrenoids and in the reconstituted minimal natural system is mainly based on substrate partitioning and mass action kinetic effects, rather than direct effects of the pyrenoid matrix on the structure and/or catalytic properties of Rubisco. This highlights the significance of physicochemical principles in the basic function of CCMs and opens the possibility for future applications. Our approach to use simple phase-separating IDPs to establish synthetic and minimal CO_2_/carbon-concentrating structures successfully concludes recent protein design efforts with similar goals^54^ and provides an alternative strategy for complex pyrenoid reconstruction efforts and/or transplantation approaches aimed at enhancing carbon fixation.

## Methods

### Reagents and materials

Unless otherwise stated, chemicals were obtained from Sigma-Aldrich and Carl Roth in the highest purity and quality. NaH^14^CO_3_ and K^14^CN were obtained from Hartmann Analytics and PerkinElmer. Reagents for cloning were obtained from New England Biolabs, Macherey-Nagel, Qiagen and Thermo Fisher Scientific. For first tests, commercial D-RuBP sodium salt hydrate (>90% purity, R0878, Sigma-Aldrich) was used. For detailed characterizations, enzymatically synthetized RuBP following published protocols^55^ was employed. For the determination of specificity constants, [1-^3^H] RuBP was synthetized enzymatically from D-[2-^3^H] glucose (PerkinElmer) following published protocols^56^. The Rubisco inhibitor CABP was previously synthetized in two batches using either cold or K^14^CN using published protocols^55^. The concentration of the cold CABP batch was previously determined by inhibiting a Rubisco stock containing a known active site concentration (as quantified by [^14^C]-CABP) with varying concentrations of cold CABP and used with the stated concentration. Unless otherwise stated, all data were analysed and processed using Python 3.10 with the packages matplotlib 3.8.2 (ref. ^57^), pandas 2.1.4 (ref. ^58^), scipy 1.11.4 (ref. ^59^), numpy 1.26.2 (ref. ^60^), openCV 4.9.0 (ref. ^61^), scikits-odes 2.7.0 (refs. ^62–64^), logomaker0.8 (ref. ^65^) and statsmodels 0.14.1 (ref. ^66^).

### Phylogenetics and ancestral sequence reconstruction

To infer the evolutionary relationships of EPYC1_CR_ and homologues, amino acid sequences were gathered by online BLASTP on 6 September 2022 using the amino acid sequence of EPYC1_CR_ from *C. reinhardtii* (XP_001690584.2) as the initial query. Sequences were aligned using MUSCLE 3.8.31 (ref. ^67^) followed by tree inference by IQ-TREE 2.2.0.3 (ref. ^68^) and a best-fit model of evolution as determined by Modelfinder 2.2.0.3 (ref. ^69^), with gamma-distributed among-site rate variation and fixed base frequencies. Two models of evolution were identified by IQ-TREE and tested: first, Mitochondrial Inverterbrate^70^ when considering all amino acid evolution models, and second, Jones–Taylor–Thornton^71^ when restricting to the most commonly chosen amino acid models of LG^72^, Jones–Taylor–Thornton and WAG^73^. Both approaches led to the same inferred sequences with respect to the natural variation.

Ancestral sequences and posterior probabilities of ancestral states were reconstructed at internal nodes as implemented in IQ-TREE, using the same evolutionary model as per tree inference. Gap assignment of ancestral sequences was determined using Fitch parsimony with PastML (v.1.9.34)^74^, yielding our final tree. Ancestral sequences contain states with the highest posterior probabilities at all sites selected.

The resulting evolutionary tree was rooted using timetree.org^42^ to the oldest node. Estimates of atmospheric gas concentrations were directly adapted from the website.

### Protein production and purification

Protein-coding DNA was codon-optimized and produced by Twist Biosciences or Azenta Life Sciences. Constructs were cloned to the C terminus (Gibson assembly) or the N and C termini (Golden Gate cloning using PaqCI) of the *R. rubrum* CbbM coding region in a His-bdSUMO plasmid acquired from Addgene^75^ using OneShot Top10 cells (Invitrogen). All plasmids and primers are supplied in Supplementary Data 1 and 2. The constructs were expressed in OneShot BL21-AI (Invitrogen) cells in TB media. An ON seed culture was used to inoculate 6 l of production culture. Cells were harvested by centrifugation at 5,000*g* in a Beckman Coulter Avanti JXN-26 centrifuge equipped with a JLA-8.100 rotor. The cells were resuspended in 50 mM Tris, 10 mM MgCl_2_, 500 mM NaCl, 2 mM 2-β-mercaptoethanol (2-BME) and 10 mM imidazole at pH 8.5. Cells were broken using a microfluidizer at 18,000 psi with four cycles. Subsequently, the cell debris was sedimented using ultracentrifugation at 100,000*g* (Sorvall WX+, Thermo Scientific, with T-647.5 fixed angle rotor 6 × 50 ml). The supernatant was then filtered using 0.2-nm syringe cap filters. The filtrate was subsequently applied to a 50- or 150-ml superloop (Cytiva) and from there on two stacked 5-ml HisTrap columns (Cytiva). The protein on the column was washed with 50 mM imidazole, 50 mM Tris, 500 mM NaCl and 2 mM 2-BME, pH 8.5, until the UV 280-nm signal was stable. Subsequently, 5 ml of 2 mg ml^−1^ bdSNEP1 in 50 mM imidazole, 50 mM Tris, 500 mM NaCl and 2 mM 2-BME, pH 7.5, supplemented with 2 mM dithiothreitol (DTT) was applied to the columns and incubated for five hours at 20 °C. The cleaved protein was then eluted using 10 ml of 10 mM imidazole, 50 mM Tris, 500 mM NaCl and 2 mM 2-BME, pH 8.5, and captured in the superloop. Subsequently, the protein was applied to a 16/600 200-pg Superdex SEC-column in 50 mM Tris, 500 mM NaCl and 10 mM MgCl_2_, pH 8.5, with 10 % v/v glycerol, isocratically eluted, and 2-ml fractions were collected. The fractions were analysed for protein content and impurities using SDS*–*PAGE and then concentrated to ~100 μM using 10-kDa or 30-kDa MWCO concentrators (Amicon Ultra Filters, Merck). The protein was aliquoted into 20-μl volumes, frozen in liquid nitrogen and stored at −80 °C until further use. For cryoEM structure elucidation within condensates, the purest SEC fraction (without glycerol) of CbbM–EPYC1_CR_Δα was used. For crystallization, the CbbM fraction with the highest purity from SEC using 20 mM Tris and 100 mM NaCl, pH 8.5, was concentrated to 5 mg ml^−1^. For both cryoEM and crystallography, the enzyme fractions were activated using 10 mM NaHCO_3_ in the presence of a twofold molar excess of CABP for about 30 min.

RbcLS_CR_ was natively purified from *C. reinhardtii* adapted from Wunder et al.^21^. For this, the cells were grown with 17.4 mM acetate-supplemented TAP media for several days until maximum optical density (3–3.5) was reached. Cells were harvested by centrifugation at 5,000*g* in a Beckman Coulter Avanti JXN-26 centrifuge equipped with a JLA-8.100 rotor. The cells were resuspended in 100 mM Tris, pH 8.0, 100 mM NaCl, 0.5 mM EDTA, pH 8.0, 5 mM DTT and EDTA-free protease inhibitor tablets (Roche). Cells were broken using a microfluidizer at 18,000 psi with four cycles. Subsequently, the cell debris was sedimented using ultracentrifugation at 47,000*g* (Sorvall WX+, Thermo Scientific, with T-647.5 fixed angle rotor 6 × 50 ml). The soluble supernatant was applied to a Q-HP anion exchange column (Cytiva) mounted on an Äkta Pure systems (Cytiva) equilibrated in 50 mM NaCl, 20 mM Tris-HCl, pH 8.0, and 2.5 mM DTT and fractionated using a linear gradient between 50 mM and 1 M NaCl. The correct fractions were determined via SDS–PAGE and subsequently desalted using PD10 desalting drip columns (Cytiva) in 50 mM NaCl, 20 mM Tris, pH 8.0, and 2.5 mM DTT. The sample was then applied to a 10-ml self-packed ResourceQ15 (Cytiva) and fractionated with the same gradient as before. Rubisco-containing samples were determined via SDS–PAGE, pooled and applied to a Superdex 200 Increase 10/300 (Cytiva) equilibrated in 20 mM Tris (pH 8.0), 50 mM NaCl, 1 mM DTT and 5% v/v glycerol. Rubisco-containing fractions were determined via SDS–PAGE, pooled, concentrated using 10-kDa MWCO Amicon spin filters (Merck), flash-frozen in liquid nitrogen and stored at −70 °C.

### Radiometric carboxylation assays

#### Time-curve assays

The assays were adapted from Schulz et al.^76^. Prior to each assay, protein stock concentration was measured via BCA assay (Thermo Scientific), and the condensate formation was assessed via light microscopy (Supplementary Fig. 2) under reaction conditions. Reactions were conducted with NaH^14^CO_3_ or NH_4_H^14^CO_3_ stocks with specific radioactive activities between 4 and 8 mCi mmol^−1^. The solubility of CO_2_ in water (mol l^−1^ atm^−1^) was calculated using the Henderson–Hasselbalch equation using previously published values^77^. Time-curve assays were performed aerobically in 50–100-μl volumes in 1.5-ml crimp-neck amber vials (VWR) at 25 °C and 300 rpm using a Thermomixer Pro (CellMedia). For each dataset, four time points with three replicates each for both CbbM and CbbM–IDP in the same shaker were measured. To this end, 1 μM CbbM/CbbM–IDP, 0.1 mg ml^−1^ CA and 1 mM MgCl_2_ was activated for 5 min using 5 mM ^14^CO_3_^2−^ stocks in 50 mM Tris, pH 7.5–8.0 (depending on condensate stability). Subsequently, 0.3 mM RuBP was added to start the reaction. After 2, 10, 30 and 90 min, the reactions were quenched using 10% final v/v of 50% v/v formic acid. The reactions were left to evaporate on a heating block at 95 °C ON. Residual material was resuspended in 500 μl ddH_2_O, transferred to 5-ml scintillation vials, and mixed with 4.5 ml scintillation cocktail (ROTISZINT HighCapacity, Carl Roth). The amount of acid-stable radioactivity was quantified using a Beckman LS 6000 scintillation counter. Initial rates were computed using the 2 min time point and normalized with the respective active site content. Active site content was quantified via [^14^C]-CABP binding. To this end, the same reaction mixtures with cold CO_3_^2−^ and CABP instead of RuBP were incubated in the reaction buffer supplemented with 500 mM NaCl to prevent condensate formation for 1 h. The mixtures were centrifuged for 10 min at 10,000*g*. Subsequently, 35 μl supernatant was applied to a SERAgel Pro 250 column (ISERA) on an Agilent 1260 HPLC system and isocratically eluted in the same buffer. Fractions containing the protein were collected (2 ml total volume), mixed with 12 ml scintillation cocktail and measured on the scintillation counter. Active site content was computed from the known counts/concentration of the [^14^C]-CABP stocks. For total carboxylation, the ratios of the maximally reached scintillation count in the time curves of Rubisco and Rubisco fusion were used. If necessary, multiple datasets were combined by first computing the ratios of individual datasets and then averaging the ratios to account for fluctuations in assays. Errors were propagated to the ratios and subsequently to the final ratio via empirical error propagation. For anaerobic initial rate comparisons, assay buffers, vials and stocks were degassed with CO_2_ free nitrogen for at least 3 h.

#### ^3^H specificity assays

The assays were performed exactly as reported in Schulz et al.^76^. Briefly, CbbM and CbbM–IDPs were incubated in 20-ml septum capped glass scintillation vials containing 1 ml 50 mM Tris, pH 7.5–8.0, 1 mM MgCl_2_ and 0.1 mg ml^−1^ carbonic anhydrase. Reaction mixtures were equilibrated in defined gas mixtures containing 995,009 ppm O_2_ and 4,991 ppm CO_2_ (Air Liquide) before the reactions were initiated by the addition of 1 mM [1-3H]-RuBP. After 1 h, the products were dephosphorylated using alkaline phosphatase (10 U per reaction) for another 30 min and subsequently separated on an HPX-87H column (Bio-Rad). ^3^H-glycerate and ^3^H-glycolate peaks were quantified using liquid scintillation counting. The specificity factor *S*_C/O_ was calculated as described previously^56^. Reaction buffers were adapted to condensate-forming conditions using the same buffers as for the time-curve assays.

### LC–MS/MS specificity assays

The assays were adapted from Schulz et al.^76^. Buffers and vials were degassed with CO_2_ free nitrogen and equilibrated in 995,009 ppm O_2_ and 4,991 ppm CO_2_ (Air Liquide) prior to reaction start and during the reaction. Reactions were run in 0.5-ml volumes in 1.5-ml crimp-neck amber vials (VWR) equipped with gas-tight conical rubber stoppers in a Thermomixer Pro (CellMedia) at 25 °C. For each reaction, 1 μM CbbM and CbbM–IDPs were used. The reactions were supplemented with 0.1 mg ml^−1^ CA and then activated for 5 min before the addition of 1 mM RuBP. The reactions were run in quadruplicates for 30 min and quenched using 50 μl formic acid. Subsequently, 35 μl centrifuged for 2 min at 3,000*g* and submitted for LC–MS/MS measurement.

### LC–MS/MS method

Quantitative determination of RuBP, 2-PG and 3-PG was performed using an LC–MS/MS system. The chromatographic separation was performed on an Agilent Infinity II 1290 HPLC system using a SeQuant ZIC-pHILIC column (150 mm × 2.1 mm, 5-μm particle size, peek coated, Merck) connected to a guard column of similar specificity (20 mm × 2.1 mm, 5-μm particle size, Phenomoenex) and a constant flow rate of 0.1 ml min^−1^ with mobile phases consisting of 10 mM ammonium acetate in water, pH 9, supplemented with medronic acid to a final concentration of 5 μM (A) and 10 mM ammonium acetate in 90:10 acetonitrile to water, pH 9, supplemented with medronic acid to a final concentration of 5 μM (B) at 40 °C. The injection volume was 0.5 μl. The mobile phase profile consisted of the following steps and linear gradients: 0–1 min constant at 75% B, 1–6 min from 75% to 40% B, 6 to 9 min constant at 40% B, 9–9.1 min from 40% to 75% B, 9.1 to 20 min constant at 75% B. An Agilent 6470 mass spectrometer was used in negative mode with an electrospray ionization source and the following conditions: ESI spray voltage, 2,000 V; nozzle voltage, 1,000 V; sheath gas, 300 °C at 12 l min^−1^; nebulizer pressure, 20 psi; and drying gas, 150 °C at 11 l min^−1^. Compounds were identified on the basis of their mass transition and retention time compared to standards. Chromatograms were integrated using MassHunter software (Agilent). Absolute concentrations were calculated via standard addition, spiking each sample with a mixed stock solution of the target analytes at different concentrations (0 μM, 10 μM, 25 μM, 50 μM, 100 μM and 250 μM) and analysing each sample six times. Mass transitions, collision energies, cell accelerator voltages and dwell times were optimized using chemically pure standards. The parameter settings of all targets are given in Supplementary Table 5.

### HPLC–SEC measurement for determination of protein amount in condensates

CbbM–IDP amounts in the continuous phase and in non-phase-separated conditions were measured using a HPLC–SEC–UV/Vis system. First, the condensates were formed in 50 mM Tris, pH 7.5, 0 mM NaCl and 1 mM MgCl_2_ in triplicates at 1 µM in 200 µl total volume. To measure the total amount of protein in the system, the same experiment was conducted in non-condensate-forming conditions in 50 mM Tris, pH 8.5, 500 mM NaCl and 10 mM MgCl_2_. The samples were incubated at room temperature for 30 min and subsequently sedimented at 2,000*g* for 30 min. The supernatant was then sampled using 35 µl. The chromatographic separation was performed on an Agilent Infinity II 1290 HPLC system using an AdvanceBio analytical isocratic SEC column (300 mm × 4.6 mm, 2.7-μm particle size, 500-Å pore size, Agilent) at a constant flow rate of 0.2 ml min^−1^ in 50 mM Tris, pH 8.5, 500 mM NaCl and 10 mM MgCl_2_ buffer for 35 min. The injection volume was 2.5 μl. Chromatograms were exported and integrated using Python code based on rampy 0.4.9 (ref. ^78^), scipy 1.11.4 (ref. ^79^) and numpy 1.26.2 (ref. ^60^). The integrated peaks were compared between the supernatants of phase-separated and homogeneous samples, and the relative amount of total protein inside the condensates was calculated via $1-\frac{{{\rm{Area}}}_{{\rm{condensates}}}}{{{\rm{Area}}}_{{\rm{homogeneous}}}}$. Errors were propagated using empirical error propagation.

### Crystallization and X-ray structure determination

Crystallization was performed by sitting-drop vapour diffusion at 16 °C. Purified inactive CbbM in 20 mM Tris buffer (pH 8.5) containing 100 mM NaCl was mixed with a condition comprising 1.2 M ammonium chloride, 100 mM MES (pH 6) and 20% (w/v) PEG6000 in a 1:1 ratio with a final drop volume of 1 µl. The mother liquor was supplemented with 30% (v/v) PEG200 for about 1 min, before the crystals were looped and plunge-frozen in liquid nitrogen. Purified activated CbbM was crystallized in a buffer containing 20 mM Tris (pH 8.5), 100 mM NaCl, 0.2 mM CABP and 10 mM sodium bicarbonate mixed with a condition containing 200 mM Bis-Tris propane (pH 7.3) and 20% (w/v) PEG4000 in a 1:1 ratio with a final drop volume of 1 µl. The mother liquor was supplemented with 33% (v/v) PEG200 for about 1 min, before the crystals were looped and plunge-frozen in liquid nitrogen.

X-ray diffraction data (Supplementary Table 5) were collected at the PETRA III beamline P13 of DESY (Deutsches Elektronen-Synchrotron, Hamburg). The data were processed with XDS^80^. Structures were solved by molecular replacement using Phaser of the Phenix package 1.21 (ref. ^81^) using an AlphaFold2 2.2.0 (ref. ^82^) model as a search template and refined with Phenix.Refine. Manual modelling, refinement and ligand fitting as well as water picking were performed in Coot 0.9.8.3 (ref. ^83^). Final positional and B-factor refinements were performed using Phenix.Refine. Structural models for the inactive form and activated CABP bound form were deposited to the Protein Data Bank in Europe under accession numbers 9I27 and 9I26, respectively (Supplementary Table 6). The figures were made using PyMOL 2.5 (PyMOL Molecular Graphics System, Schrödinger (www.pymol.org)).

### CryoEM sample preparation, data acquisition and single-particle reconstruction

The CbbM–EPYC1_CR_Δα stock was activated using 10 mM NH_4_HCO_3_ and a twofold excess of CABP for 30 min prior to sample preparation. Subsequently, 2.7 μl 50 mM Tris and 0 mM NaCl, pH 7.5, was pipetted onto C-Flat R2/1 300 copper mesh grids that were glow-discharged for 45 s immediately before use, and 0.3 μl of the protein stock was added to the drop, resulting in a final protein concentration of ~1 μM.

The sample was blotted for 3 s with blot force 20 at 100% relative humidity and 4 °C using a Vitrobot Mark IV (Thermo Scientific). Grids were plunge-frozen in liquid ethane cooled by liquid nitrogen, clipped into Autogrid cartridges and used for data collection immediately.

CryoEM data were acquired on a Titan Krios G3i electron microscope (Thermo Scientific), operated at an acceleration voltage of 300 kV and equipped with a BioQuantum-K3 imaging filter (Gatan). Data were measured in electron counting mode at a nominal magnification of ×130,000 (0.655 Å per pixel) with a total dose of 60 e^−^ A^−2^ (60 fractions), using the aberration-free image-shift correction in EPU (Thermo Scientific). Five images were acquired per foil hole, and the nominal defocus range for data collection was −1.2 to 2.4 μm. Foil holes were selected on the basis of capturing a sufficiently thin portion of the condensates around the too-dense parts.

Micrographs were processed in cryoSPARC 4.4.1 (Structura Biotechnology Inc.). The first 11,525 micrographs were motion- and CTF-corrected, and then low-quality micrographs were excluded, yielding 7,116 remaining micrographs. A total of 45,233 particles were manually picked since the small size of the structured domain precluded reliable automated picking in cryoSPARC. Using denoised micrographs, further template-based particle searches and several classification steps, the final structure reconstruction was done with 209,691 high-quality particles. Detailed single-particle analysis is shown in Supplementary Figs. 20 and 21.

CryoEM map fitting was initially performed in UCSF-ChimeraX 1.7 (ref. ^84^) using an AlphaFold2 2.2.0 (ref. ^82^) model as a template. The resulting model was manually refined in Coot 0.9.8.3 (ref. ^83^). Automatic refinement of the structure was performed using phenix.real_space_refine in Phenix (v.1.21)^81^. The resulting model and electron density map were deposited in the Protein Data Bank in Europe and the Electron Microscopy Data Bank with the accession codes 9I28 and EMD-52579, respectively. Model statistics are listed in Supplementary Table 6.

### Confocal microscopy for fluorescence detection of carboxylation, polarity estimation, pH estimation and carbonic anhydrase partitioning

The activity in the condensates was qualitatively observed using a fluorescence coupled assay (Supplementary Fig. 8). The precise concentrations are displayed in Supplementary Table 8. Pgmi was produced in-house, horseradish peroxidase was acquired from Sigma (P8375), enolase from Sigma (E6126), pyruvate kinase from Roche (10109045001), carbonic anhydrase from Sigma (C2624) and pyruvate oxidase from Sigma (P4591). First, all components except Rubisco and RuBP were pipetted into 384 glass-bottom plates (Cellvis, 384-well glass-bottom plate P384-1.5H-N). The protein was then added, and the mixture was checked for condensate formation. After Rubisco activation for several minutes, RuBP was added to observe the increase in fluorescence in a confocal epi-fluorescence microscope (Leica TCS SP8 X, Diode 405 nm laser/WLL combination, transmission PMT and HyD detector). Images were recorded at 1,024 × 1,024 pixels with a pixel size of 80.17 nm and a rate of 0.644 images per second for 25 minutes. Samples were excited at 2% LP at 570 nm and recorded with a 585–600 nm detection window.

The polarity of condensates was estimated using the assay reported in Küffner et al.^50^. Condensates were formed with 1 μM of CbbM–IDP fusion in 50 mM Tris, 0 mM NaCl and pH 7.5. Images were recorded at 1,024 × 1,024 pixels with a pixel size of 41.01 nm and a rate of 0.375 images per second. Samples were excited at 2% LP with 405 nm and detected in a window from 425 nm to 784 nm with a 5-nm bandwidth and 5-nm steps. Subsequently, the intensities were extracted, and a gamma distribution was fitted to the data to estimate the maximum-intensity wavelength. Polarity was inferred using a standard curve using reported literature data (Supplementary Fig. 13)^85,86^.

In analogy to the polarity estimation inside the condensates, we adapted an intra-condensate pH estimation assay from Stoffel et al.^87^. A standard curve from pH 6 to 10 using 50 mM Bis-Tris (pH 6 and 6.5) and Tris buffers with 50 µM 5-(and-6)-Carboxy SNARF-1 (Thermo Scientific) in a Tecan Infinity platereader (Tecan) at excitation of 520 nm and emission from 530 nm to 750 nm was recorded (Supplementary Fig. 14). Condensates were formed in pH 7.5, 50 mM Tris, 1 mM MgCl_2_ and 50 µM 5-(and-6)-Carboxy SNARF-1. Spectra of the condensates were recorded via lambda stacks in the confocal microscope. Images were recorded at 2% LP at 405 nm and an emission window from 518 to 706 nm using a 5-nm step size and a 5-nm detection window. Regions of interest of the condensates were extracted, averaged and compared against the standard curve to estimate the internal pH.

Carbonic anhydrase partitioning was assessed using Atto425-tagged carbonic anhydrase. To this end, we incubated 2 mg ml^−1^ carbonic anhydrase from bovine erythrocytes (Sigma-Aldrich) with a ~2-fold molar excess of Atto425-Malemide (ATTO-TEC) for 12 h on ice. Subsequently, we separated the tagged protein from unreacted Atto425 with a 10/30 Superdex 200 pg increase (Cytiva) isocratically in 50 mM Tris, 500 mM NaCl and 2 mM 2-BME, pH 8.5. The tagged protein was concentrated using 500 μl Amicon 10 kDa spin filters. Condensates were formed using 1 μM fusion protein in 50 mM Tris and 0 mM NaCl, pH 8.0. After 5–10 min, a mixture of 10% CA-ATTO425 and 90% CA with a total final concentration of 0.1 mg ml^−1^ was added to the condensates. After a couple of minutes, images were recorded at 1,024 × 1,024 pixels with a pixel size of 80.17 nm, a rate of 0.241 images per second and a frame averaging of 8. Samples were excited at 2% LP with 405 nm and recorded with a 465–505 nm detection window.

### Mass photometry size analysis

Microscope coverslips (1.5 H, 24 mm × 60 mm, Carl Roth) and CultureWell Reusable Gaskets (CW-50R-1.0, 50-3 mm diameter × 1 mm depth) were cleaned three times with MQ-H_2_O and isopropanol followed by drying under a stream of pressurized air. Gaskets were dried further at room temperature overnight. Immersion oil (Immersion Oil 518F, Zeiss) was applied to the mass photometer (MP) objective, and dry gaskets were subsequently assembled on coverslips and placed on the stage of a One^MP^ MP (Refeyn). Then, 18 μl of SEC buffer was applied to the gasket wells. After focusing, 2 μl of sample was added and rapidly mixed, and measurements were started (<15 s after sample addition). Data were acquired for 60 s at 100 frames per second using Acquire^MP^ 1.2.1 (Refeyn 1.2.1). Samples were prepared by diluting purified protein to 0.25 μM concentration in 50 mM Tris, 500 mM NaCl and 10 mM MgCl_2_, pH 8.5. MP contrast values were calibrated to molecular masses using commercial NativeMark Unstained Protein Standard (Thermo Fisher). The instrument was calibrated using a 30-fold dilution of NativeMark, which was diluted a further 10× on the instrument. Acquired images were processed using DiscoverMP 1.2.3 (Refeyn).

### Mass action and mass transfer model simulations and data fit

Differential equation systems were implemented in Python 3.13 and solved using scikits.odes based on the SUNDIALS suite^62–64^. Data were fitted using maximum likelihood estimators and globally optimized using a differential evolution algorithm from scipy.optimize^88^^,89^. The differential equation system for Rubisco was adapted from previous simulation work and is shown below^49^:

$$\frac{{\mathrm{d}}{c}_{i}^{{\mathrm{in}}}}{{\mathrm{d}}{t}}=r\left({c}_{i}^{{\mathrm{in}}}\right)+k\left({c}_{i}^{{\mathrm{out}}}-{K}_{i}{c}_{i}^{{\mathrm{out}}}\right)$$

$$\frac{{\mathrm{d}}{c}_{i}^{{\mathrm{out}}}}{{\mathrm{d}}{t}}=r\left({c}_{i}^{{\mathrm{out}}}\right)+k\left({K}_{i}{c}_{i}^{{\mathrm{out}}}-{c}_{i}^{{\mathrm{out}}}\right)$$

$$r\left({c}_{{\mathrm{RuBP}}}\right)=-r\left({c}_{{{\mathrm{CO}}}_{2}}\right)-r\left({c}_{{{\mathrm{O}}}_{2}}\right)=-E\frac{\frac{{k}_{{\mathrm{C}}}{c}_{{{\mathrm{CO}}}_{2}}{c}_{{\mathrm{RuBP}}}}{{K}_{{\mathrm{m}},{{\mathrm{CO}}}_{2}}{K}_{{\mathrm{m}},{{\mathrm{RuBP}}}}}}{1+\frac{{c}_{{{\mathrm{CO}}}_{2}}{c}_{{\mathrm{RuBP}}}}{{K}_{{\mathrm{m}},{{\mathrm{CO}}}_{2}}{K}_{{\mathrm{m}},{{\mathrm{RuBP}}}}}}-E\frac{\frac{{k}_{{\mathrm{O}}}{c}_{{{\mathrm{O}}}_{2}}{c}_{{\mathrm{RuBP}}}}{{K}_{{\mathrm{m}},{{\mathrm{O}}}_{2}}{K}_{{\mathrm{m}},{{\mathrm{RuBP}}}}}}{1+\frac{{c}_{{{\mathrm{O}}}_{2}}{c}_{{\mathrm{RuBP}}}}{{K}_{{\mathrm{m}},{{\mathrm{O}}}_{2}}{K}_{{\mathrm{m}},{{\mathrm{RuBP}}}}}}$$

$$r\left({c}_{3{\mathrm{-}}{{\mathrm{PG}}}}\right)=2r\left({c}_{{{\mathrm{CO}}}_{2}}\right)+r\left({c}_{{{\mathrm{O}}}_{2}}\right)$$

$$r\left({c}_{2{\mathrm{-}}{{\mathrm{PG}}}}\right)=r\left({c}_{{{\mathrm{O}}}_{2}}\right)$$

$$k=\frac{D}{R}$$

$${K}_{E}=\frac{\frac{1}{1-{N}_{R}}+\varPhi -1}{\varPhi }$$

where ${c}_{i}^{{\mathrm{in}}}$ and ${c}_{i}^{{\mathrm{out}}}$ denote the concentrations of compound *i* inside and outside of the condensates; *k* and *K*_*i*_ denote the mass transfer coefficient and the partition coefficient of the respective compound; *r*(*c*_*i*_) denotes the rate of the respective compound; *E* denotes the active site concentration; *k*_C_ and *k*_O_ denote the catalytic constants of carboxylation and oxygenation, respectively; and ${K}_{{\mathrm{m}},{{\mathrm{CO}}}_{2}}$, ${K}_{{\mathrm{m}},{{\mathrm{O}}}_{2}}$ and ${K}_{{\mathrm{m}},{{\mathrm{RuBP}}}}$ denote the Michaelis constant of CO_2_, O_2_ and RuBP, respectively. The diffusion coefficient (*D*) was assumed to be 10 μm^2^ s^−1^ on the basis of small-molecule diffusion across condensates^49^, and the average condensate radius (*R*) was assumed to be 10 μm at a phase volume fraction *Φ* of 1% on the basis of estimations from previous measurements^49^. The average radius was assumed to linearly scale with the phase volume fraction in a first approximation.

### Reporting summary

Further information on research design is available in the Nature Portfolio Reporting Summary linked to this article.

## Supplementary information

Supplementary Information Supplementary Figs. 1–22 and Tables 1–8.

Reporting Summary

Supplementary Video 1 Video corresponding to Fig. 2d–g.

Supplementary Data 1 Plasmid list.

Supplementary Data 2 Primer list.

## Source data

Source Data Fig. 2 Statistical source data.

Source Data Fig. 3 Statistical source data.

Source Data Fig. 4 Statistical source data.

Source Data Fig. 6 Statistical source data.

## Data Availability

Structures and electron density maps are available through the Protein Data Bank (9I26, 9I27 and 9I28). Source data are provided with this paper.
