# Supplementary material for: Bottom-up reconstruction of synthetic pyrenoids provides insights into the mechanisms and evolution of carbon concentration by EPYC1 proteins
Source: Nat Plants. 2026 Aug 14;12(8):1622–36. doi: 10.1038/s41477-026-02349-x (PMC13481261; doi:10.1038/s41477-026-02349-x)
Supplement: Supplementary file 1 [file 41477_2026_2349_MOESM1_ESM.pdf]

nature plants

Supplementary information

https://doi.org/10.1038/s41477-026-02349-x

Bottom-up reconstruction of synthetic pyrenoids provides insights into the mechanisms and evolution of carbon concentration by EPYC1 proteins

In the format provided by the authors and unedited

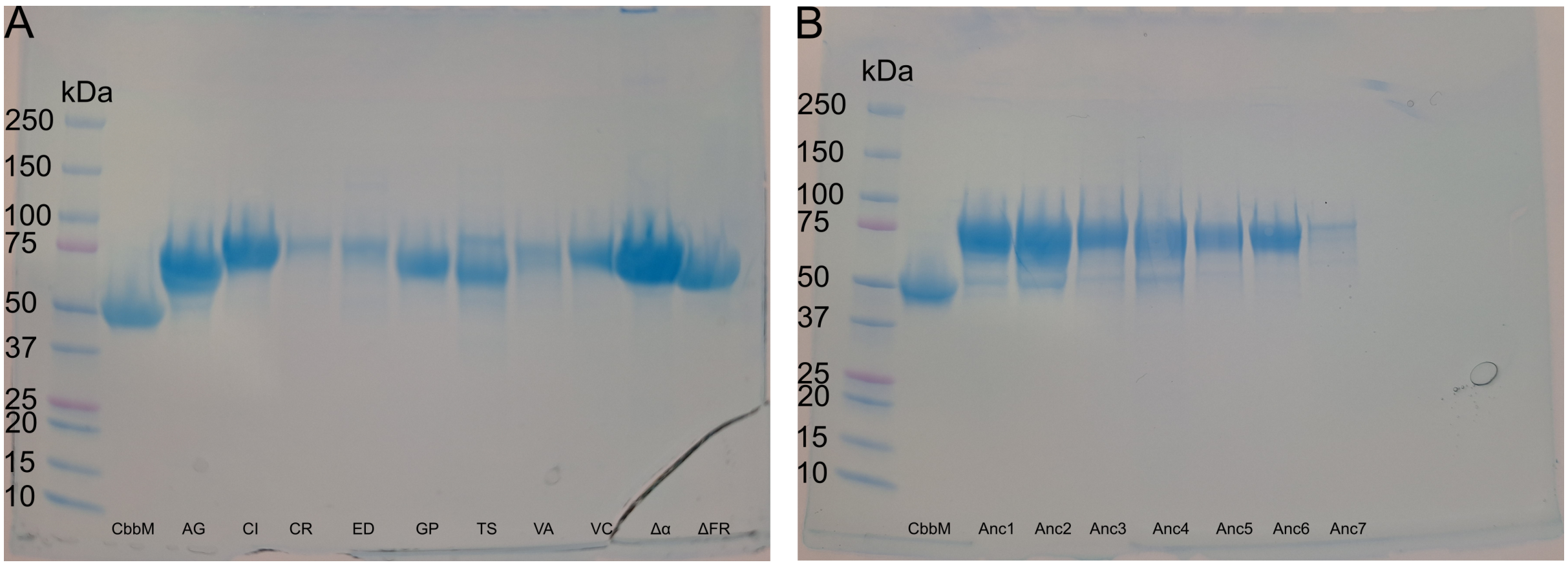

Supplementary Figure 1 SDS-PAGE gels of investigated proteins. (A) SDS-PAGE gel of CbbM and CbbM-IDP fusion proteins from the EPYC1$_{CR}$ homologs and deletion mutants. All homologs are Δctp (chloroplast targeting protein) to assure correct localization to the cytosol for recombinant purification. (B) SDS-PAGE gel of CbbM and CbbM-IDP fusion of EPYC1 ancestors.

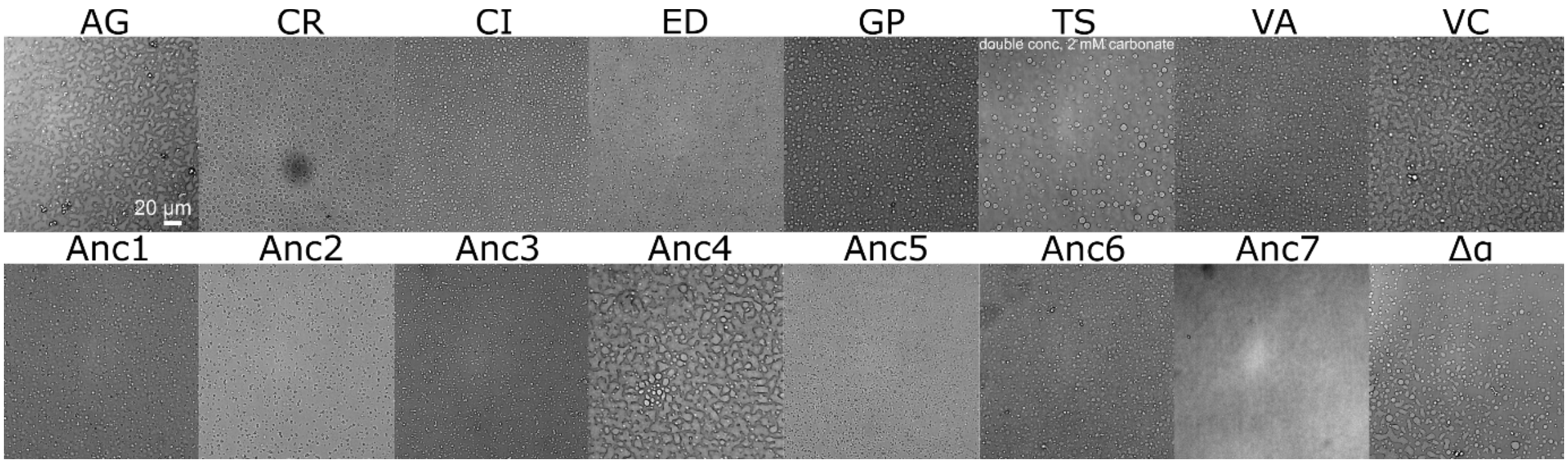

Supplementary Figure 2 Microscopy images of CbbM-IDP fusions under reaction conditions. Microscopy images of CbbM-IDP fusion proteins at 1 µM concentration, 0.1 mg/mL CA, 50 mM Tris, pH 7.5-8.0, 5 mM $NaHCO_3$ (Δα, CR, CI) or NH_4CO_3 (all ancestors, ED, GP ,TS, VA, VC). Each condensation test was repeated three times.

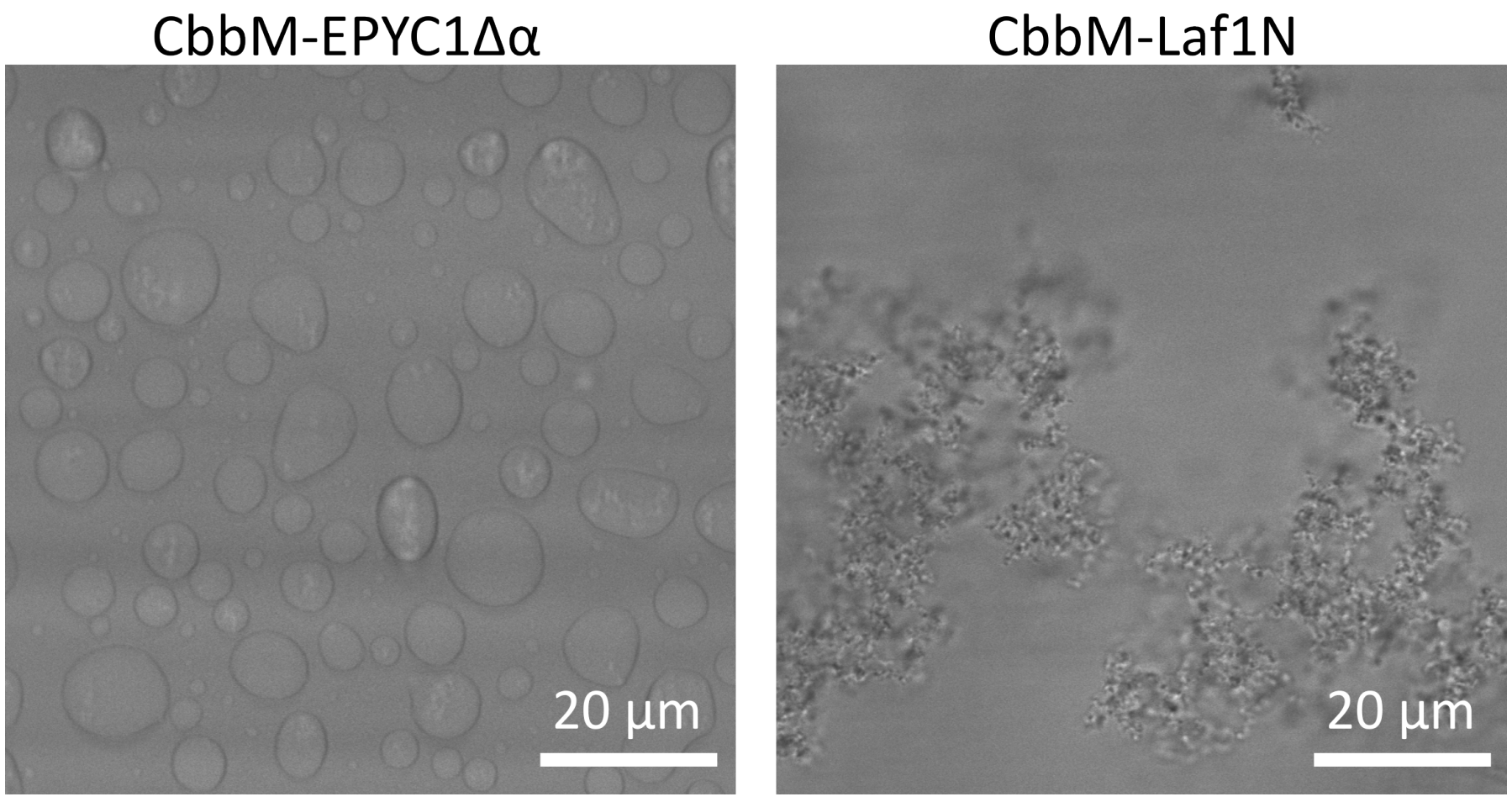

Supplementary Figure 3 Microscopy images of CbbM-EPYC1$_{CR}$Δα and CbbM-Laf1. Transmission microscopy images of condensates (CbbM-EPYC1$_{CR}$Δα) and aggregates (CbbM-Laf1N) at 1 µM protein concentration in 50 mM Tris, 0 mM NaCl, pH 8.0. CbbM-EPYC1$_{CR}$Δα forms condensates, while CbbM-Laf1N shows solidified aggregates. Each condensation screen was repeated four times.

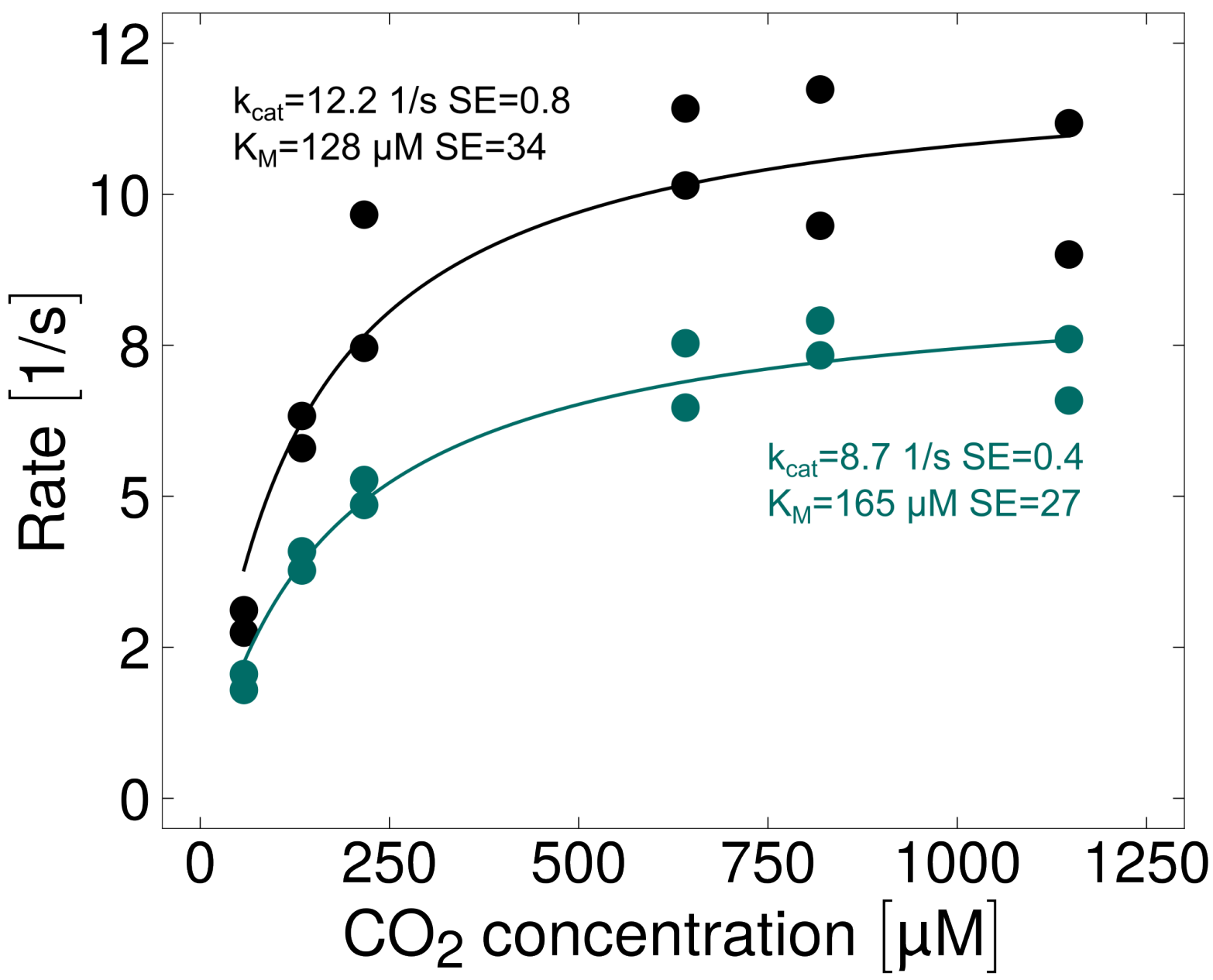

Supplementary Figure 4 Michaelis Menten kinetics of CbbM and U2AF65N-CbbM-U2AF65N. Michaelis Menten kinetics of CbbM (black dots) and U2AF65N-CbbM-U2AF65N (teal dots) are displayed. Assays were conducted at pH 8.0 in 50 mM EPPS, 10 mM $MgCl_2$, 1 mM EDTA and varying $^{14}CO_3^{2-}$ concentrations for 120 s each. Active site content was determined via [^{14}C]-CABP inhibitor binding and subsequent separation on an in house packed Sephadex G50 fine Tricon 5/100 column. Each concentration was measured in independent duplicates.

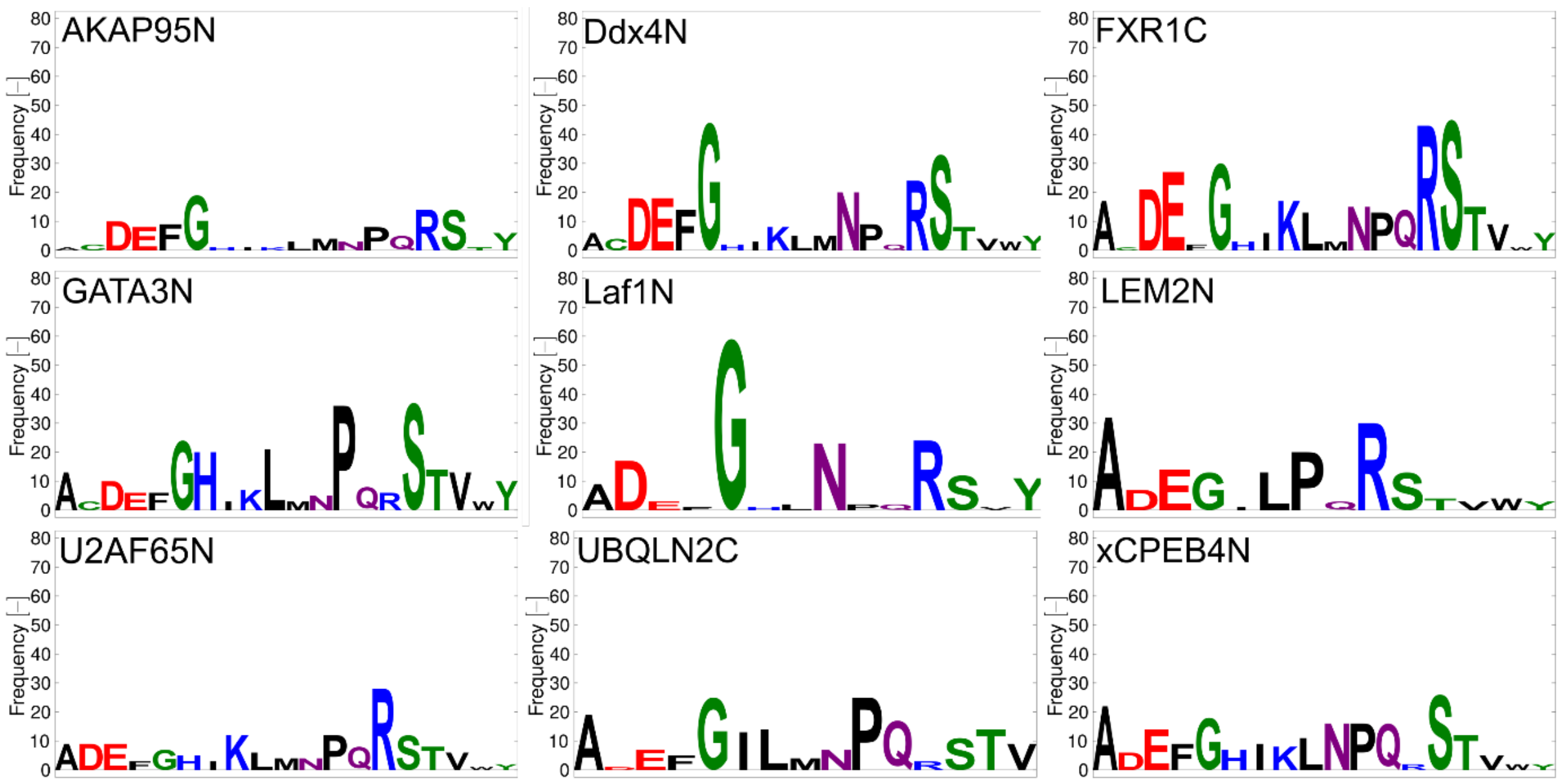

Supplementary Figure 5 Logo plots indicating amino acid composition in different non-EPYC1 related IDPs. Sequences were extracted from literature indicating clear LLPS behaviour of the respective IDPs[3,9,30–37]. Plots were created with logomaker package in python[89].

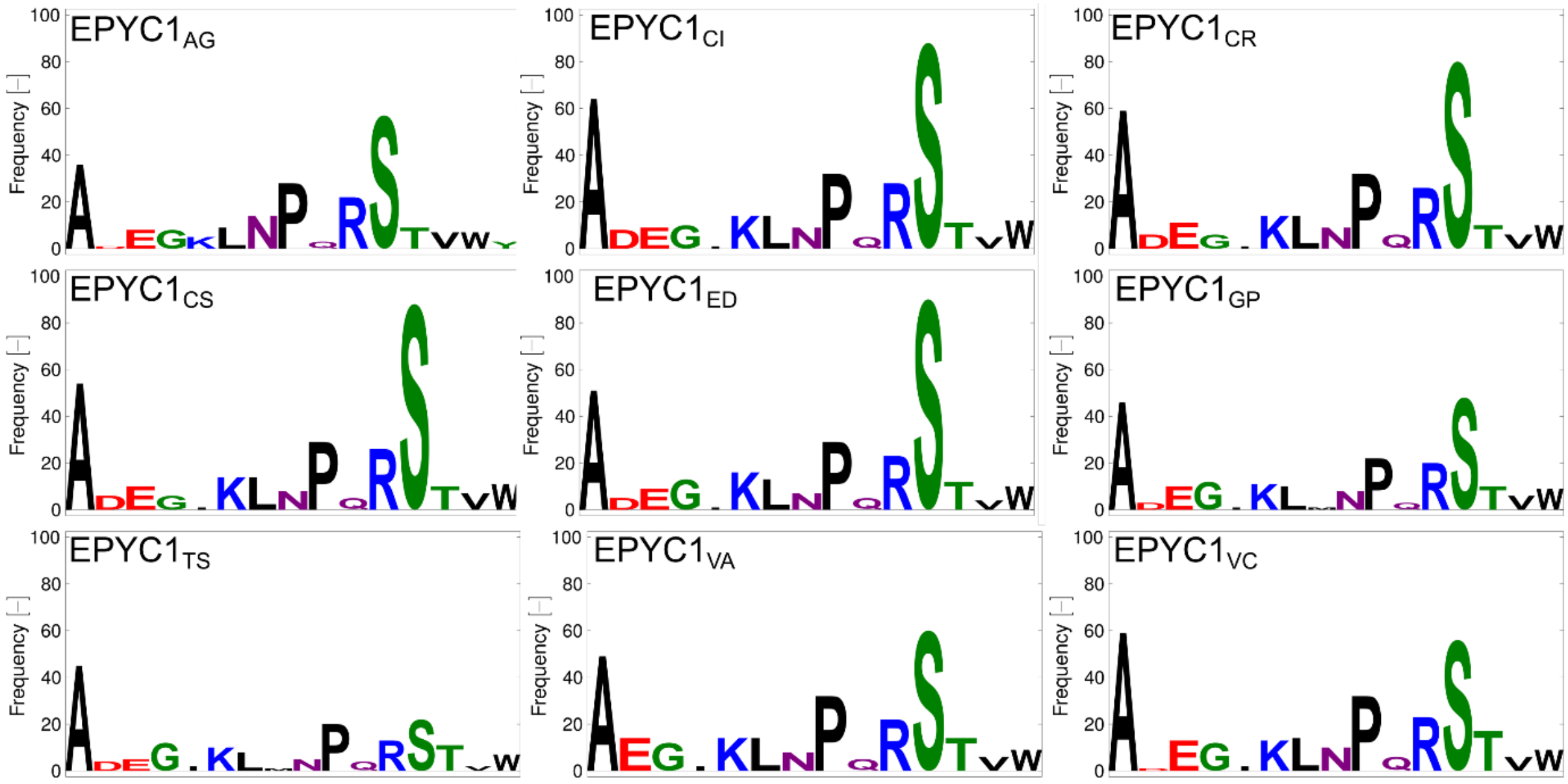

Supplementary Figure 6 Logo plots indicating amino acid composition in different EPYC1 homologues of the EPYC1$_{CR}$ from *C. reinhardtii.* Sequences were blasted using NCBI protein BLAST at the 06.09.2022. Plots were created with logomaker package in python[89].

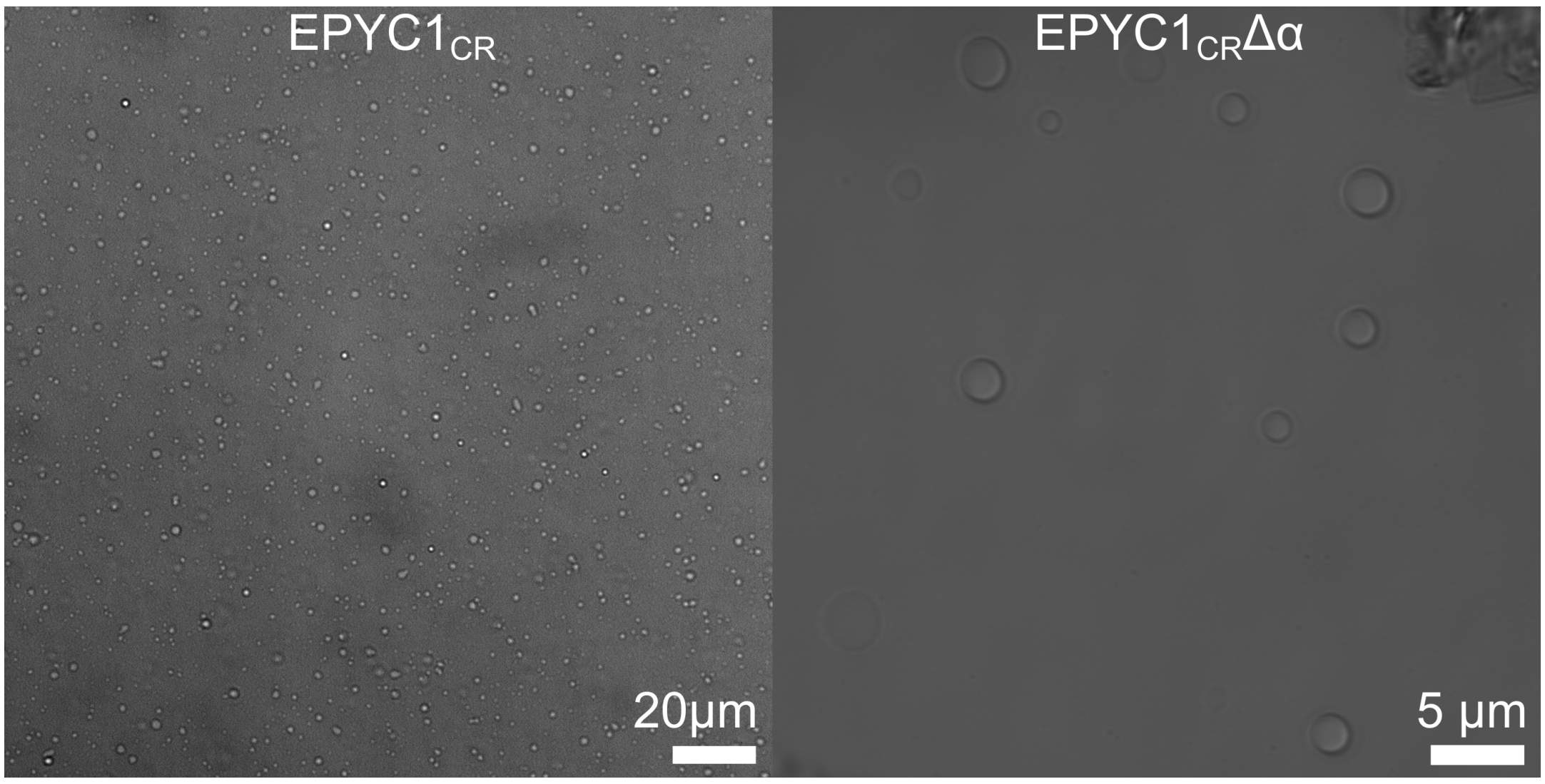

Supplementary Figure 7 EPYC1$_{CR}$ and EPYC1$_{CR}$Δα undergoing homotypical LLPS. On the left a representative bright-field image of EPYC1$_{CR}$ undergoing homotypical LLPS at pH 8.0, 50 mM Tris, 1 mM $MgCl_2$, and 40 µM protein concentration is shown from two condensation screens. On the right a representative transmission PMT image of EPYC1$_{CR}$Δα undergoing homotypical LLPS at pH 8.0, 50 mM Tris, 1 mM $MgCl_2$, and 10 µM protein concentration is shown from three condensation screens.

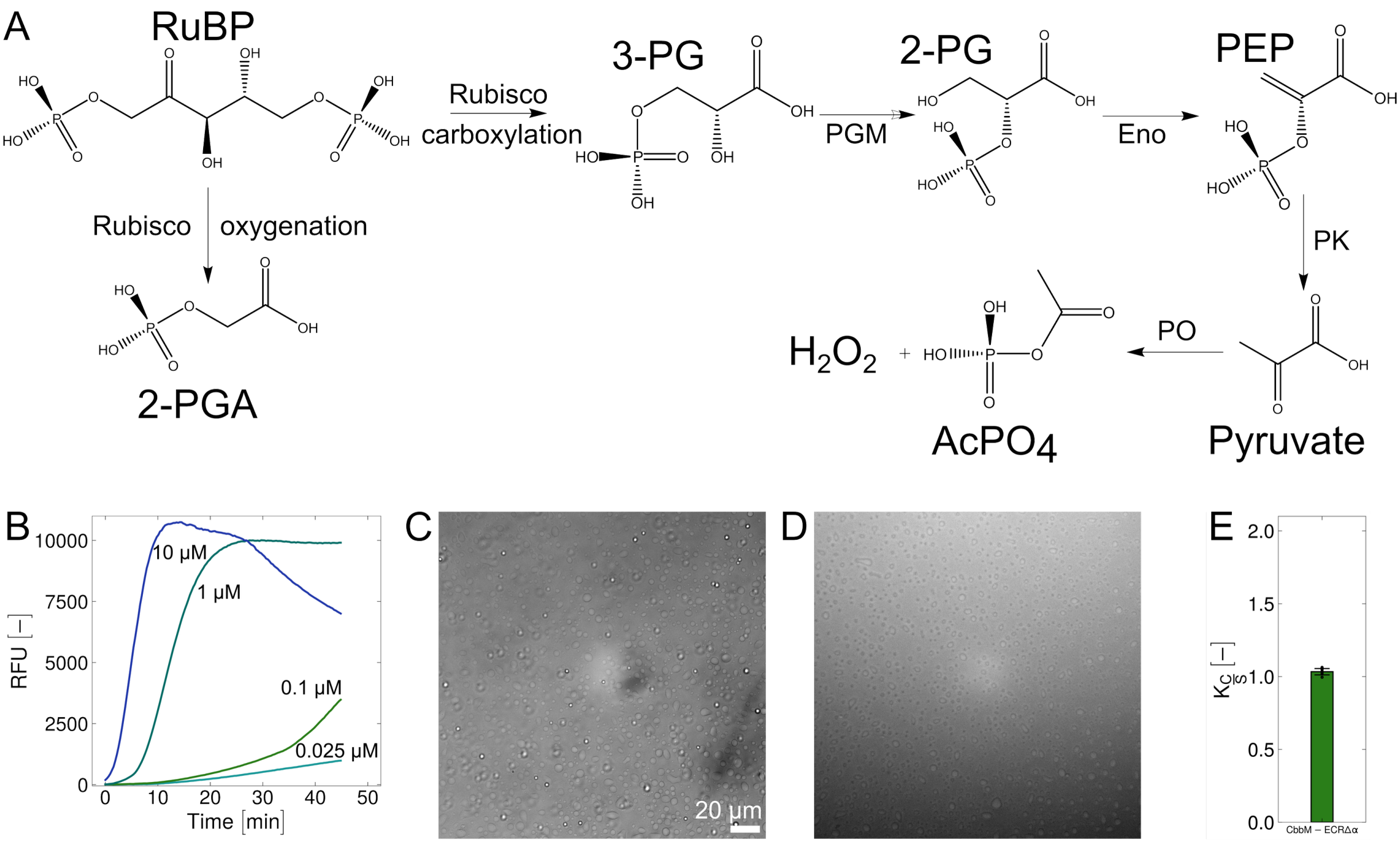

Supplementary Figure 8 Fluorescence Rubisco carboxylation activity assay. (A) RuBP can be carboxylated yielding 3-PG or oxygenated yielding 2-PGA by Rubisco. Phosphoglycerate mutase shifts the phosphate group from position 3 to 2 on 3-PG. Using an enolase the glycerate can be rearranged yielding phosphoenol pyruvate, which subsequently gets dephosphorylated via a pyruvate kinase. Pyruvate is finally oxidized to acetyl phosphate and hydrogen peroxide catalyzed by pyruvate oxidase. Hydrogen peroxide evolution is detected using horseradish peroxidase and Amplex Red. (B) LoD plot of the fluorescence assay with varying CbbM concentrations recorded in a platereader at 530 nm excitation and 590 nm emission showing sensitivity from 0.025 to 10 µM CbbM. (C) Bright-field image of 1µM CbbM-EPYC1$_{CR}$Δα in reaction buffer with 25 µM resorufin standard added. (D) Fluorescence image corresponding to C using 540-560 nm excitation and 570-620 emission at 5% laser power. (E) Partitioning of resorufin into the condensates formed by CbbM-EPYC1$_{CR}$Δα showing equal distribution across condensates and supernatant. Error bar indicates standard deviation from three images each from three independent replicates.

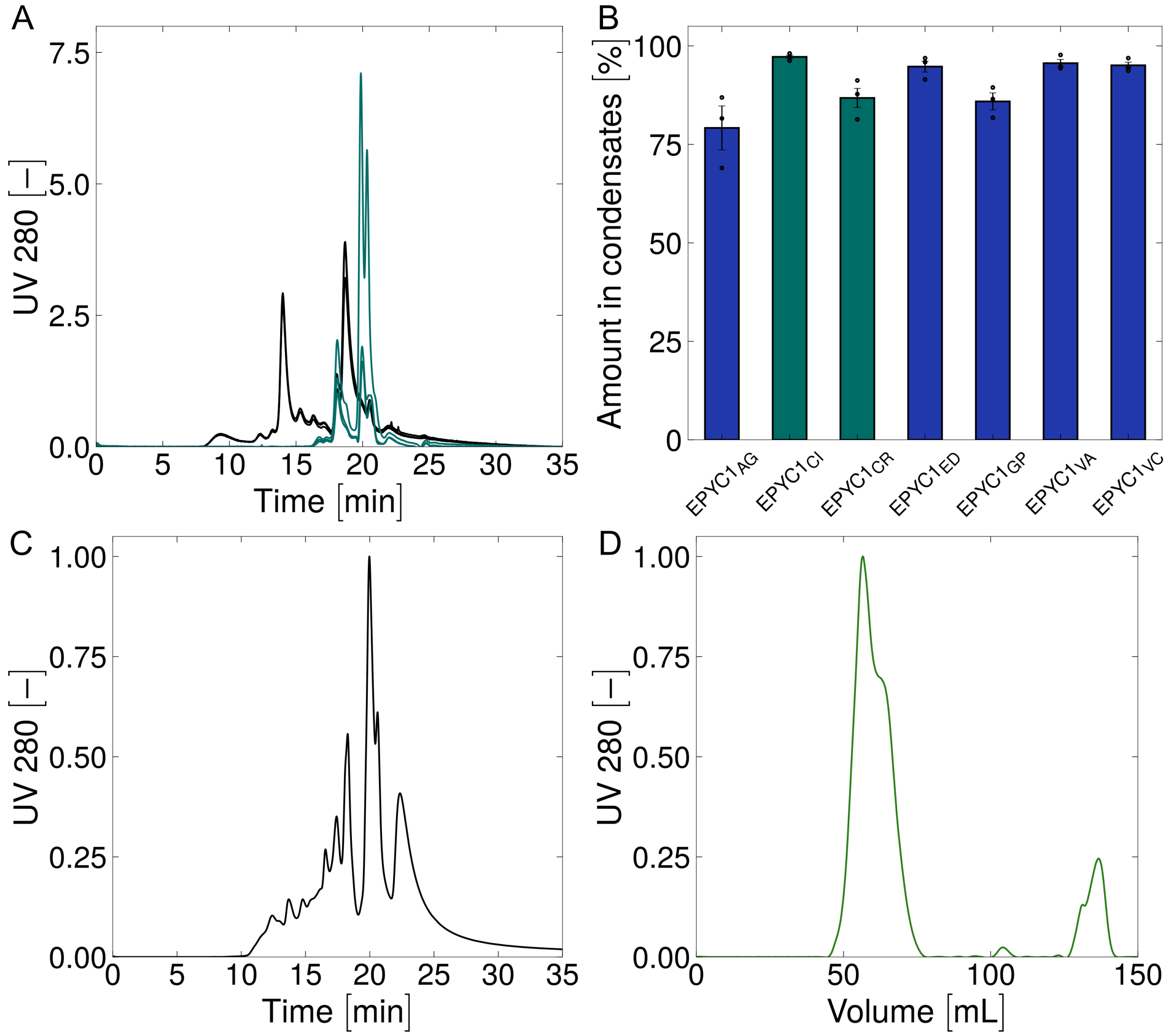

Supplementary Figure 9 Sedimentation SEC assay to determine protein amount in condensates. (A) Representative chromatograms of CbbM-EPYC1$_{CI}$ in no condensate forming conditions (pH 8.5, 50 mM Tris, 500 mM NaCl) (black) and in the supernatant of condensate forming conditions (pH 7.5, 50 mM Tris, 0 mM NaCl, 1mM $MgCl_2$) (green) in triplicates. (B) Relative amount of protein in the condensates for all extant CbbM-EPYC1 fusion proteins calculated as described in material and methods. Integration bounds for the chromatograms were choosen until the peak for the expected CbbM monomer ~ 18-19 min. Specifically, integration bounds of 8-17.75 min, 8-17.25 min, 8-18.75 min, 8-17.5 min, 8-17.75 min, 8-17.75 min, and 8-17.75 min were used. For each protein three independent replicates were used. Error bars denote standard deviation. (C) Chromatogram of RbcLS from *S. elongatus* showing multiple states ranging from the SSU/LSU monomers to full assemblies in the analytical SEC explaining the polydispersity in this analytical method. (D) Preparative SEC chromatogram using a SD 200 16/600 column for the purification of CbbM-EPYC1$_{CI}$ corresponding to proteins in (A) and to the SDS Page (Supplementary Figure 1). Exclusively the first peak was collected without the shoulder upon investigation of the SDS-PAGE gel of the individual fractions.

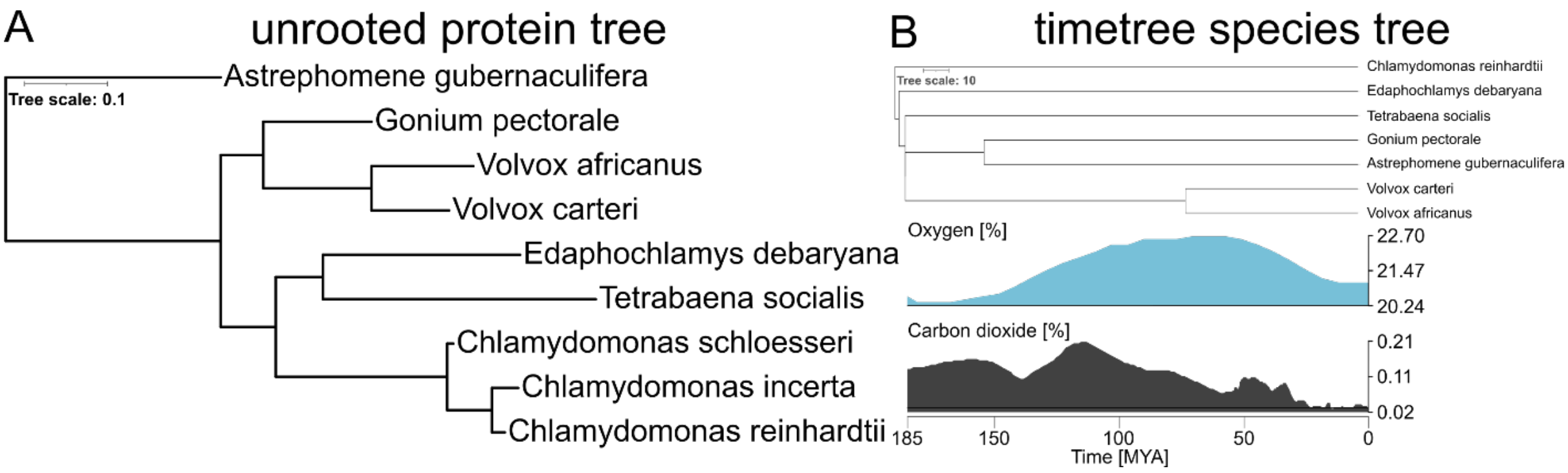

Supplementary Figure 10 Protein and species tree of the EPYC homologues and respective species. (A) The IQ-TREE result from ASR relating the EPYC homologues to each other is shown[68]. (B)The species tree of the EPYC homologues containing *Chlamydomonadales* species from timetree.org is shown[43]. The species tree was

used to root the protein tree to the oldest evolutionary node. Additionally, age, oxygen and carbon dioxide data in relation to the species tree is depicted.

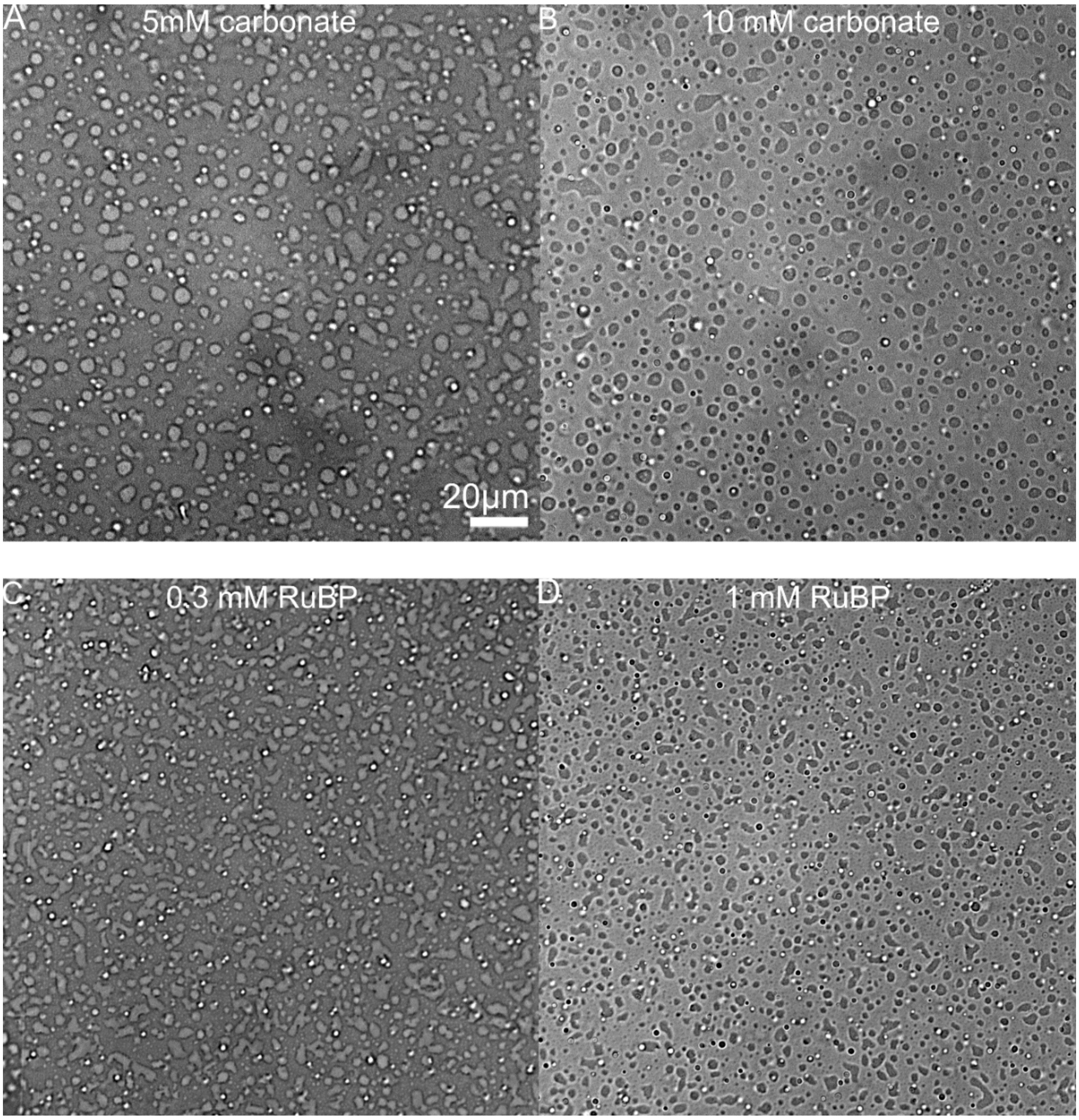

Supplementary Figure 11 CbbM-EPYC1$_{CR}$ condensates under different reaction conditions (A) Brightfield microscopy images of the condensates formed by CbbM-EPYC1$_{CR}$ using 1 µM protein in 50 mM Tris, pH 7.5, 0.1 mg/mL CA, 5 mM $NaHCO_3$, 0.3 mM RuBP, 10 mM $NaHCO_3$ (B), 0.3 mM RuBP (C; different experiment to A) and 1 mM RuBP (D). Images are representative from two independent replicates.

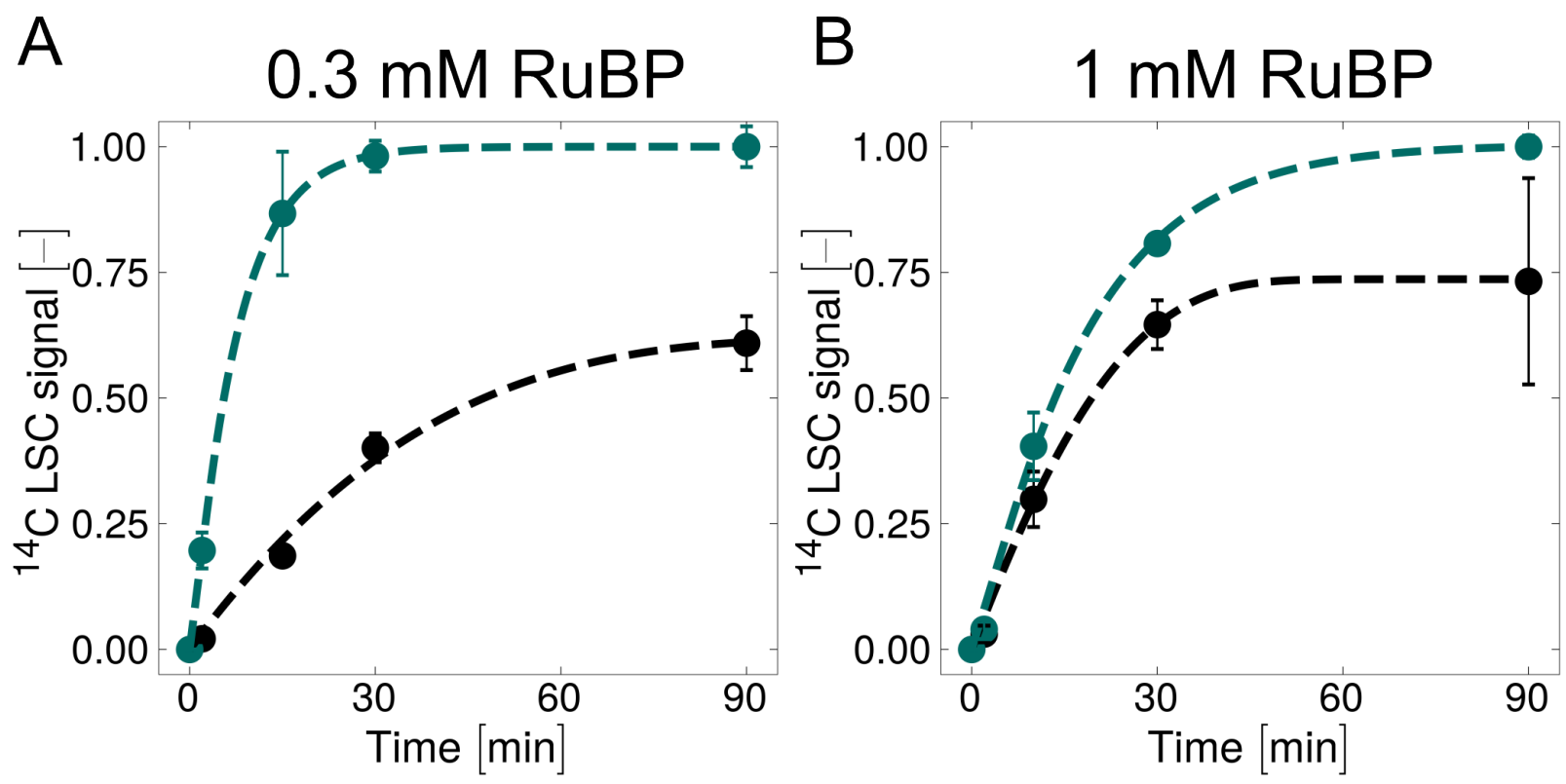

Supplementary Figure 12 Mass action kinetic fitting using generalized Michaelis-Menten kinetics and mass transfer equations. (A) Data fitting of mass action-mass transfer models to the time curve data in aerobic conditions at 0.3 mM RuBP from condensates formed by CbbM-EPYC1$_{CR}$ (teal) and from homogeneous CbbM (black). (B) Data fitting as in A to data obtained with 1 mM RuBP. Error bars denote standard errors from three independent replicates.

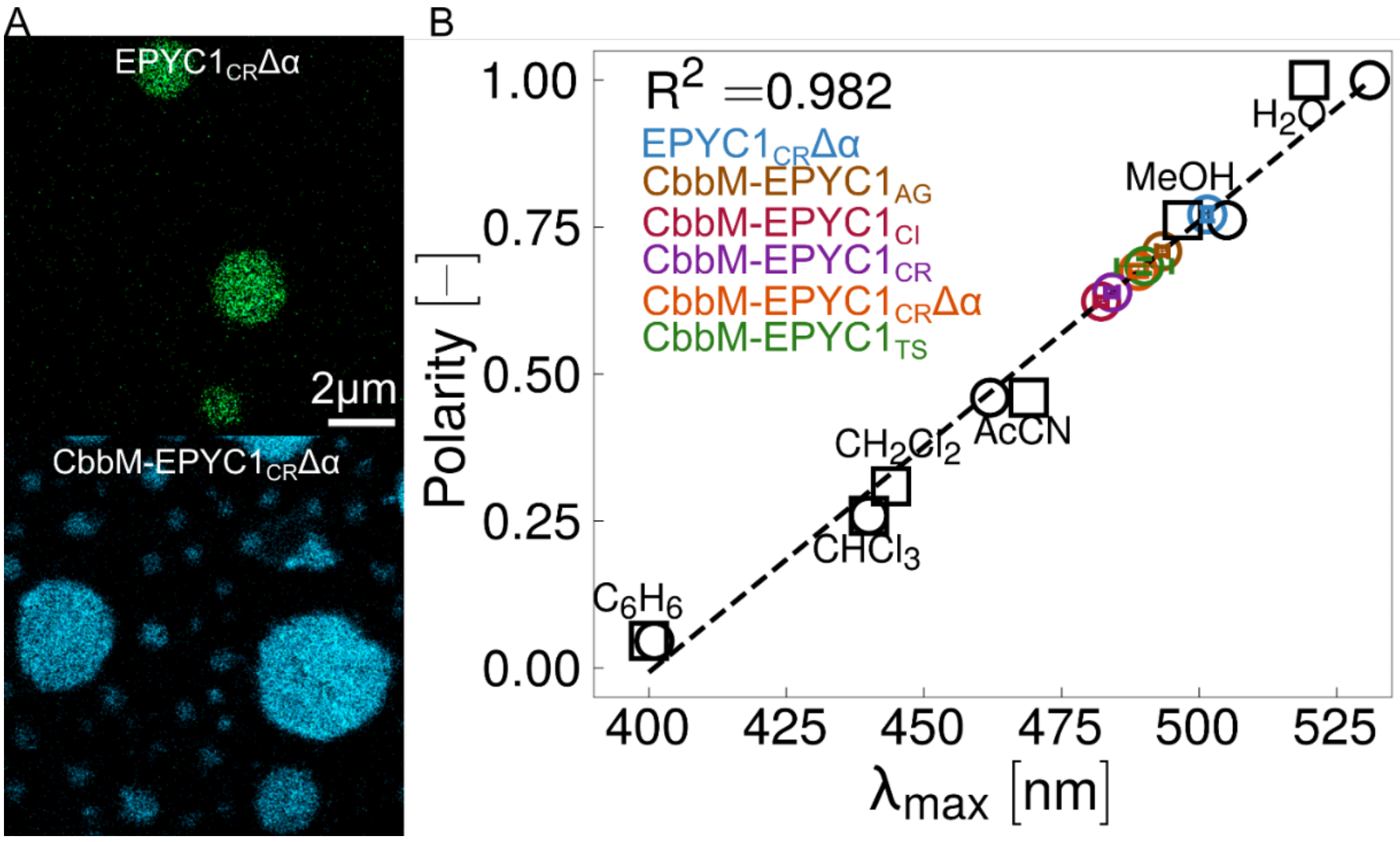

Supplementary Figure 13 PRODAN polarity estimation of condensates formed by EPYC1 variants. (A) Representative fluorescence confocal microscope images of the condensates formed by $EPYC1_{CR}\Delta\alpha$ and by $CbbM\text{-}EPYC1_{CR}\Delta\alpha$ indicating the different maximum fluorescence emission color. Condensates were formed in 50 mM Tris, pH 8.0 using 1 µM protein (10 µM for $EPYC1_{CR}\Delta\alpha$) and ~1mM of PRODAN. The test was repeated three independent times. (B) Plot showing the standard curve using literature data[84,85] on emission maxima in different solvents and the inferred apparent polarities of the condensates formed by selected EPYC1 homologues. Error bars denote standard errors.

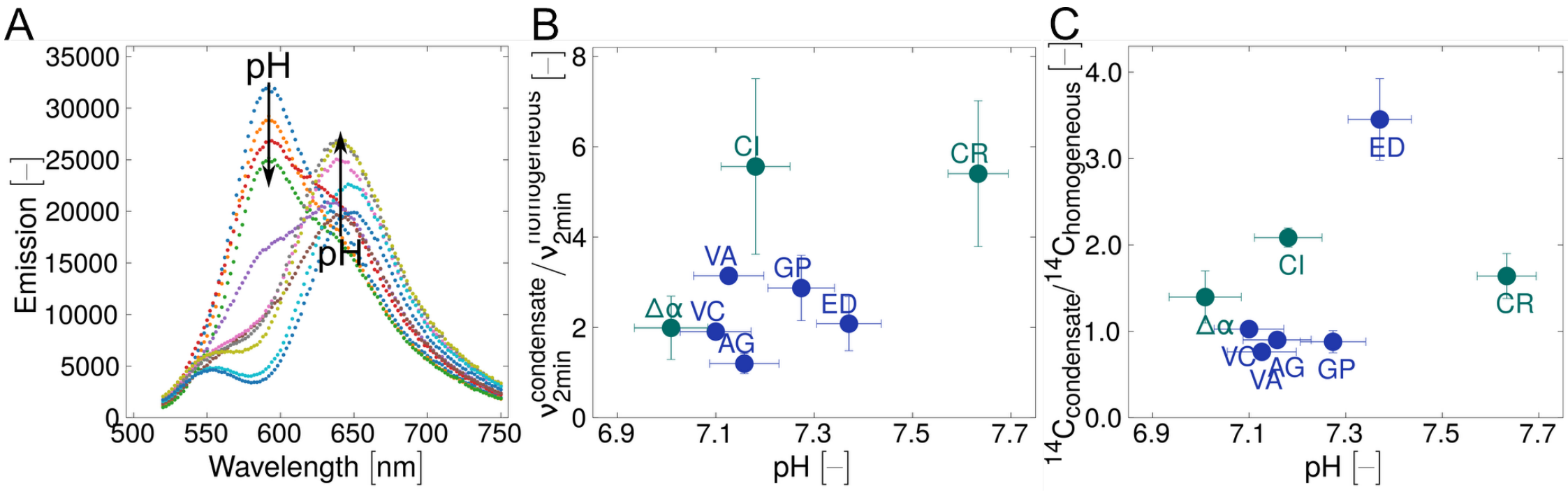

Supplementary Figure 14 SNARF-1 pH estimation assay in condensates formed by fusion proteins. (A) Platereader scan of emission spectrum at 500 nm excitation indicating the shift in at pH 6, 6.5, 7, 7.5, 8, 8.5, 9, 9.5, and 10. Each scane at each pH was performed one times and a standard curve was created from this data. (B) Initial rate ratios of CbbM-EPYC1 fusions versus internal pH of condensates inferred from the ratio of the emission maximum at 590 nm and 640 nm. Error bars denote standard errors. (C) Total carboxylation ratios of CbbM-EPYC1 fusions versus internal pH of condensates. Error bars denote standard errors.

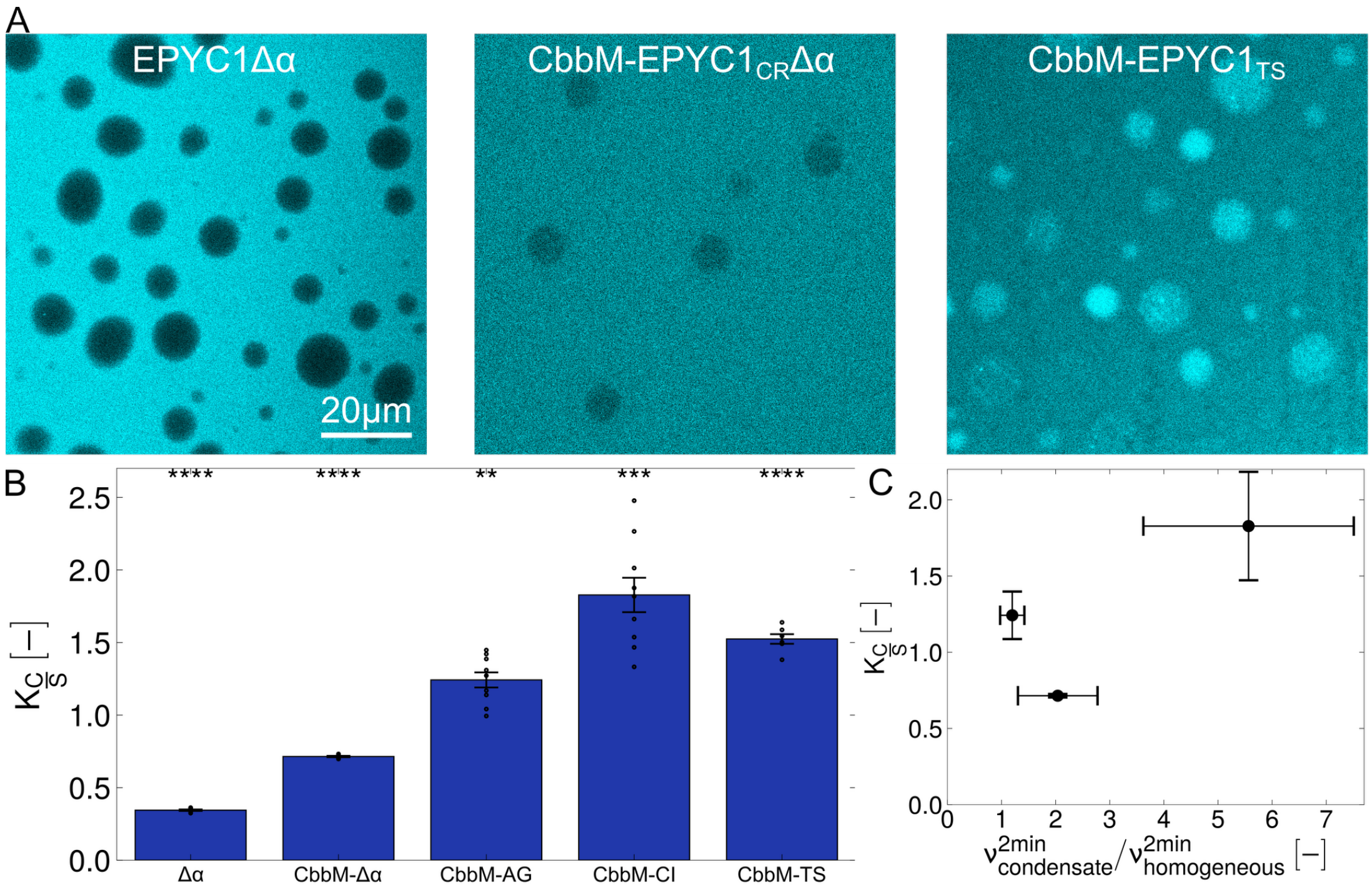

Supplementary Figure 15 Carbonic anhydrase partitioning into condensates. Condensates were formed in pH 7.5-pH 8.0, 50 mM Tris, 0 mM NaCl, ~0.1 mg/ml CA with 10% (stock v/v) ATTO-425 tagged carbonic anhydrase. At least two biological replicates, with each at least four images were recorded. (A) Representative images of different EPYC1 and CbbM-EPYC1 fusions are shown. (B) Fluorescence intensity ratios as a proxy for CA partitioning ($K_{C/S}$) were calculated by measuring intensities inside and outside the condensates in an equally distributed manner representing the mean of error bars. Error bars denote standard errors calculated from 6, 6, 9, 9, and 6 independent replicates. P-values were calculated against a population mean of 1 indicating no partitioning in either direction using a two-sided t-test ($p<0.05$=*, <0.01=**, <0.001=***, <0.0001=****, $p = 0.00000, 0.00000, 0.00230, 0.00017, 0.0003$). (C) Partitioning data from B versus initial rate data from **Figure 2**. Error bars denote standard errors.

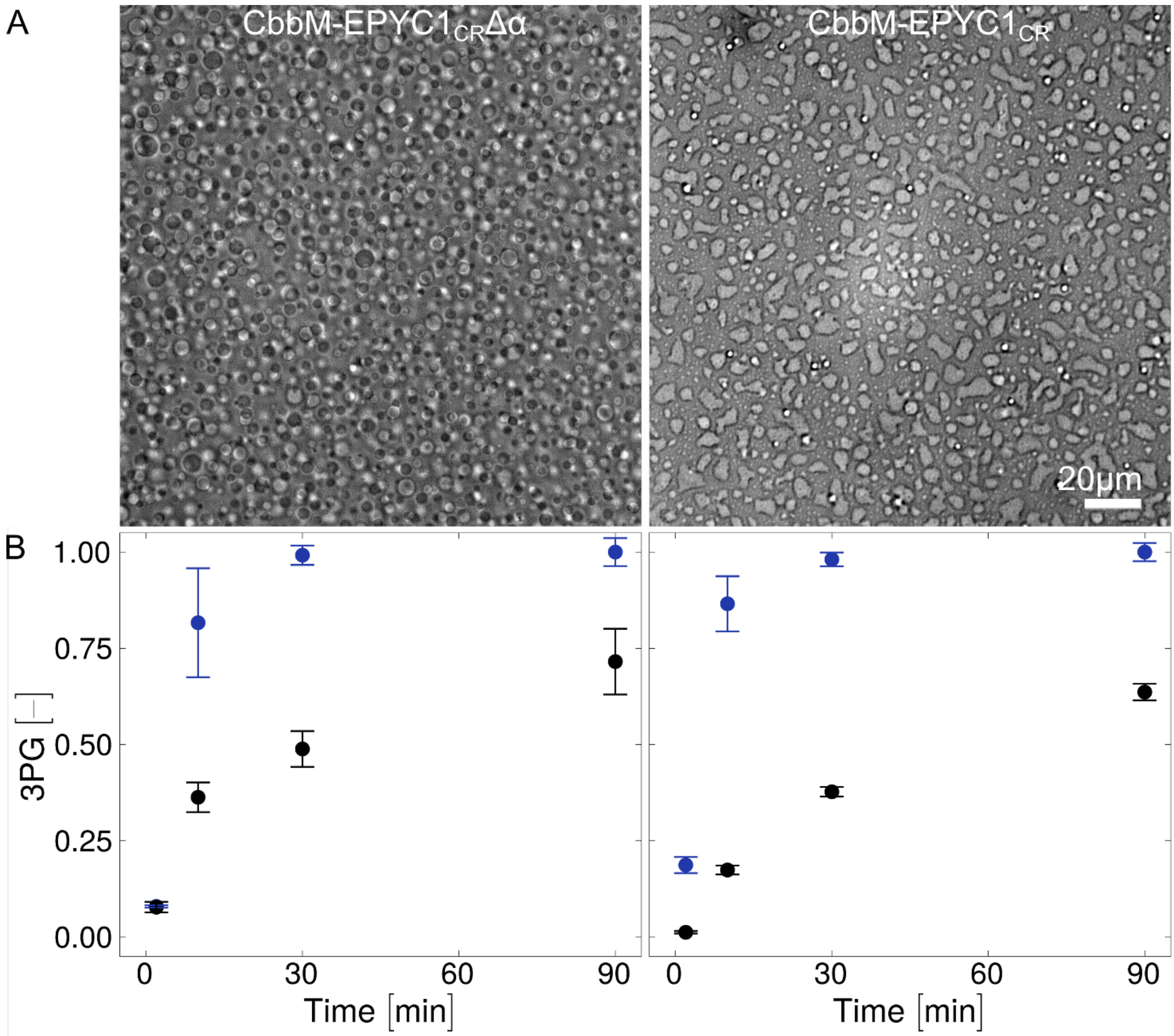

Supplementary Figure 16 Comparison of CbbM-EPYC1$_{CR}$Δα and CbbM-EPYC1$_{CR}$. (A) Brightfield microscopy images of the condensates formed by the α-helical deletion mutant of EPYC1$_{CR}$Δα (Left) and the WT EPYC1$_{CR}$ (right) using 1 μM protein in 50 mM Tris, pH 8.0, 0.1 mg/mL CA, 5 mM $NaHCO_3$, 0.3 mM RuBP from three independent condensation tests. (B) Corresponding radiometric measurements of carboxylation under aerobic conditions using 0.3 mM RuBP, 5 mM CO_3^{2-} , 0.1 mg/mL CA at 25°C. Error bars denote standard errors.

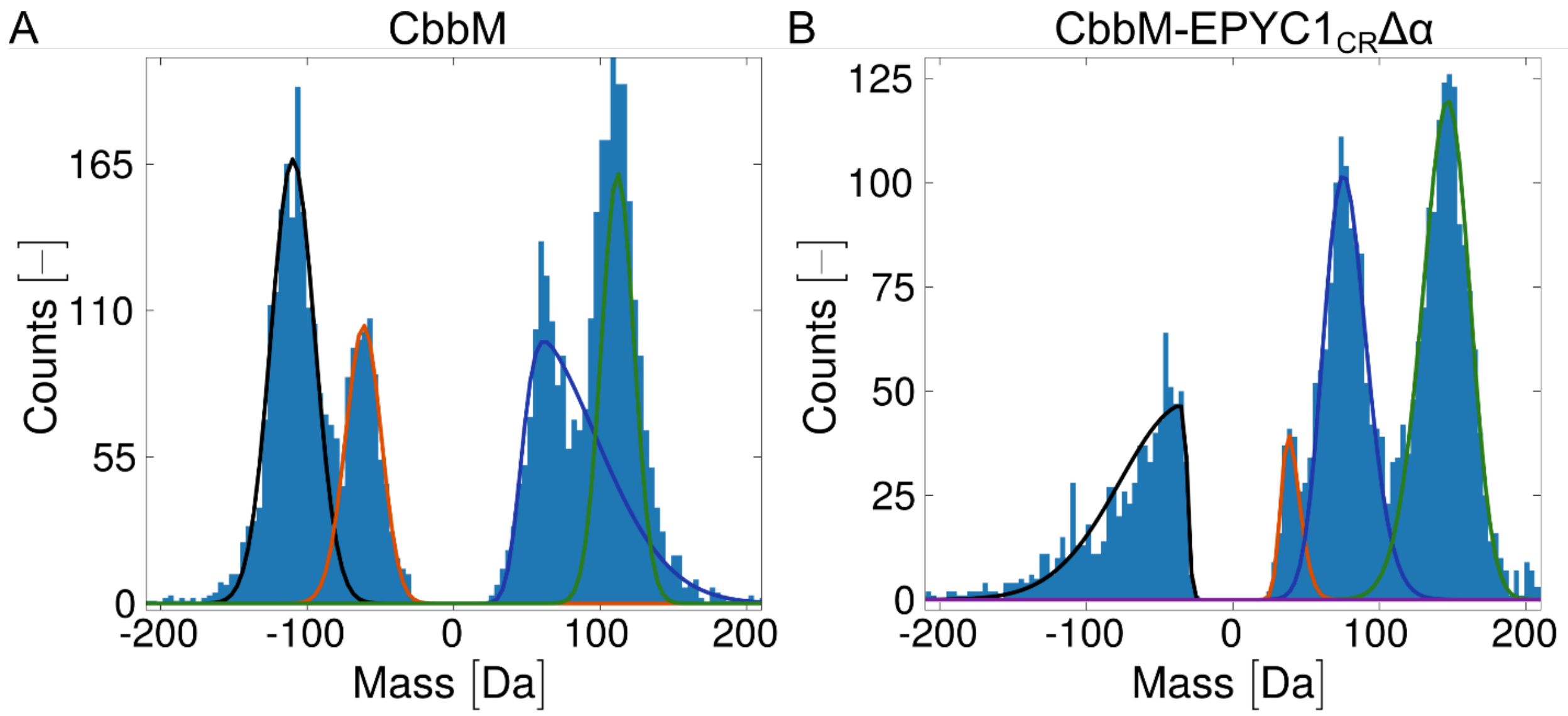

Supplementary Figure 17 Mass photometry data of solvated CbbM and CbbM-EPYC1$_{CR}$Δα. (A) Mass photometry of 25 nM CbbM in 50 mM Tris, pH 8.0, 500 mM NaCl. CbbM is visible as dimer (~100 kDa) and as

monomer (~50 kDa). Data is shown from a single replicated. (B) Mass photometry of 25 nM CbbM -EPYC1$_{CR}$Δα in 50 mM Tris, pH 8.0, 500 mM NaCl. CbbM -EPYC1$_{CR}$Δα is visible as dimer (~150 kDa) and monomer (~75 kDa). Data is shown from a single replicated. The CbbM-EPYC1$_{CR}$Δα has very low detachment events (negative range), indicating the stickiness of CbbM -EPYC1$_{CR}$Δα on the surface of the cover-slide in contrast to CbbM.

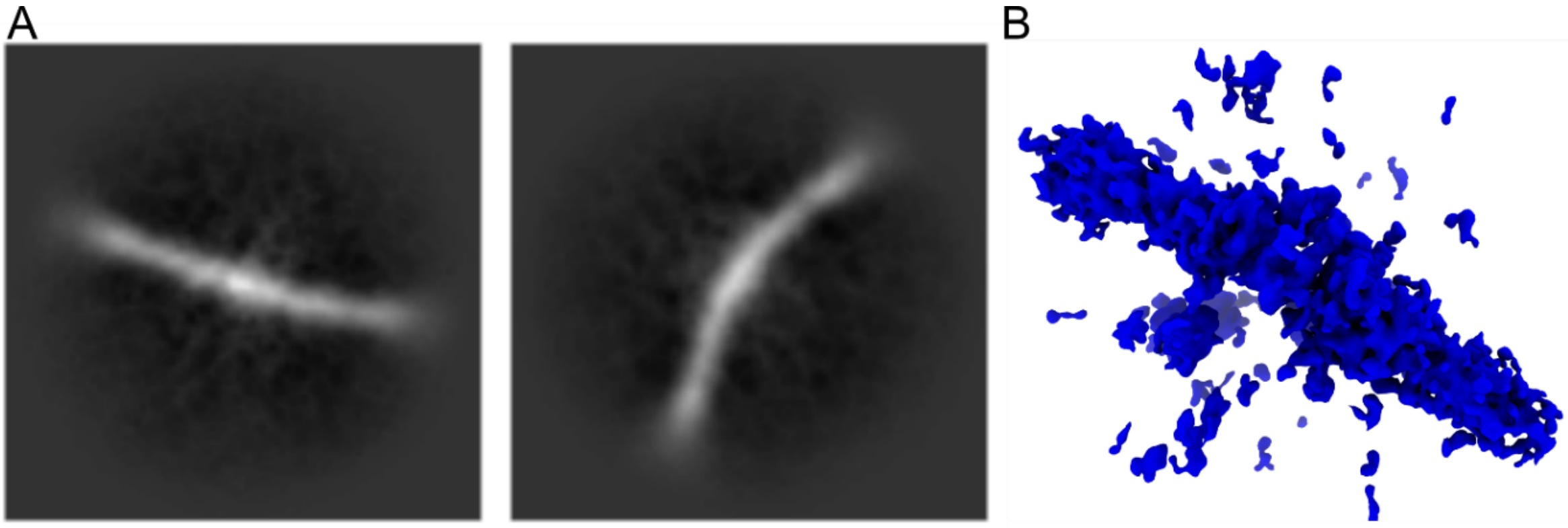

Supplementary Figure 18 Fibrillary structures emerging in 2D classification of CbbM-EPYC1$_{CR}$Δα at the edges of the condensates. (A) Representative 2D-classes emerging after 2D classification of 340804 particles showing fibrillary elongated structures suggesting EPYC1$_{CR}$Δα IDPs or bundles of them. (B) *Ab initio* volume reconstruction from 73528 particles showing elongated volumes in the size range of several IDPs bundled up. The length head-to-tail of the fibrillary structure is ~85 Å.

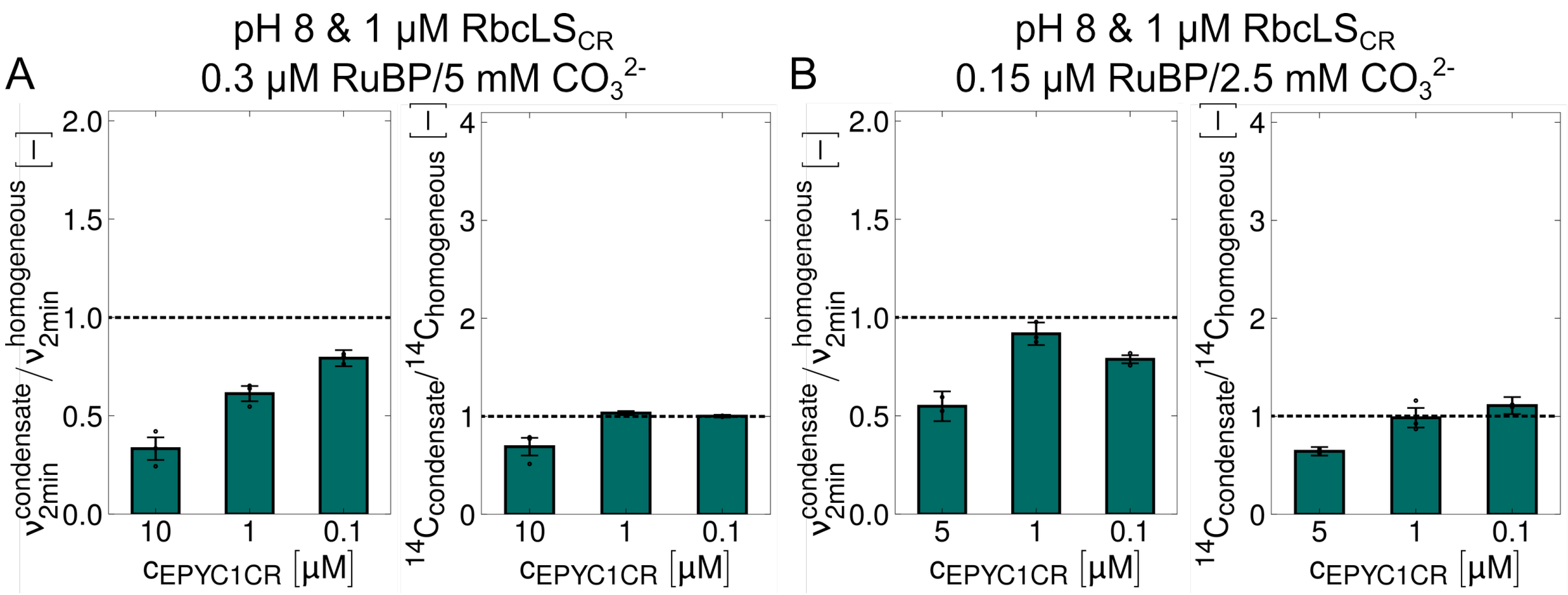

Supplementary Figure 19 Variation of RbcLS$_{CR}$:EPYC1$_{CR}$ and substrate concentration in *in vitro* reconstituted pyrenoid matrix. (A) Initial rate ratios (left) as shown in Figure 3 for the RbcLS$_{CR}$ /EPYC1$_{CR}$ system at pH 8.0, 1 μM rbcLS$_{CR}$, 5 mM NH_4HCO_3, 0.3 mM RuBP, 0.1 mg/mL CA, 1 mM $MgCl_2$, 50 mM Tris at different EPYC1$_{CR}$ concentrations representing the center of error bars. Ratios were calculated from time curve data as in Figure 3 from three independent replicates. Errors were propagated using empirical error propagation to the ratios. Error bars indicate standard errors. Maximum ^{14}C measured ratios (right) at the same conditions as (left). (B) Initial rate ratios (left) for the RbcLS$_{CR}$ /EPYC1$_{CR}$ system at pH 8.0, 1 μM RbcLS$_{CR}$, 1 mM NH_4HCO_3, 0.15 mM RuBP, 0.1 mg/mL CA, 1 mM $MgCl_2$, 50 mM Tris at different EPYC1$_{CR}$ concentrations representing the center of error bars. Ratios were calculated from time point data as in figure 3 from 3 biological replicates. Maximum ^{14}C measured ratios (right) at the same conditions as (left).

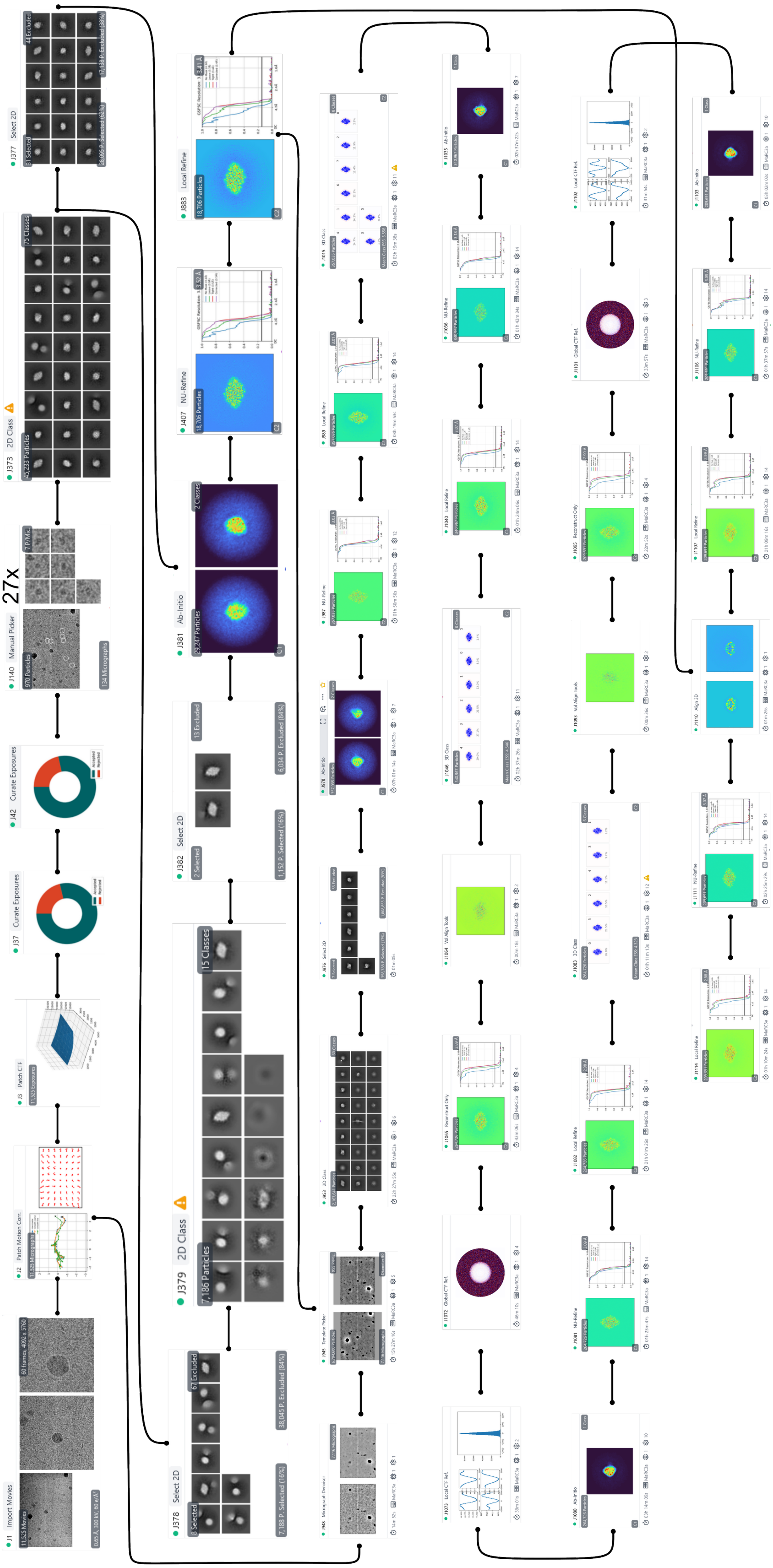
J377 Select 2D
J373 2D Class
45,233 Particles
75 Classes
27x
J140 Manual Picker
J42 Curate Exposures
J37 Curate Exposures
J3 Patch CTF
J2 Patch Motion Corr.
J1 Import Movies
J883 Local Refine
18,706 Particles
J407 NU-Refine
J381 Ab-Initio
2 Classes
J382 Select 2D
13 Excluded
2 Selected
J379 2D Class
7,186 Particles
15 Classes
J378 Select 2D
8 Selected
67 Excluded

142

Supplementary Figure 20 Cryogenic electron transmission microscopy single particle reconstruction workflow inside the condensates. Flowchart depicting the CryoEM SPA workflow in cryoSPARC.

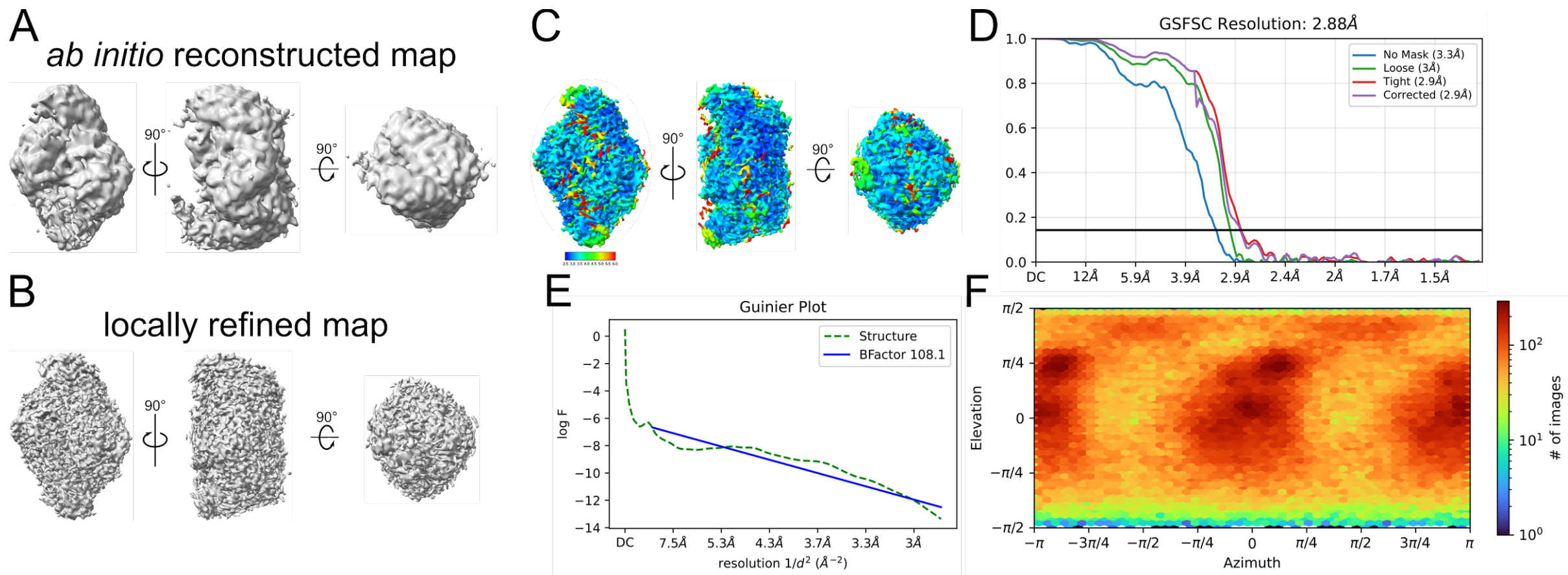

Supplementary Figure 21 Cryogenic electron transmission microscopy single particle reconstruction output. (A) Density map of the *ab initio* reconstructed volume with 209691 particles. (B) Final density map of CbbM from the CbbM-EPYC1$_{CR}$Δα inside the condensates after non-uniform refinement, local refinement, and anisotropic sharpening in Phenix is shown. (C) Local estimated resolution map of CbbM from B is illustrated. (D) The GSFCS plot from the final local refinement is presented indicating a final global resolution of 2.88 Å. (E) Guinier plot of the local refinement is shown indicating a B-factor of 108.1. (F) Viewing direction distribution plot of the local refinement is depicted indicating an overall uniform particle view with hot spots on the front view.

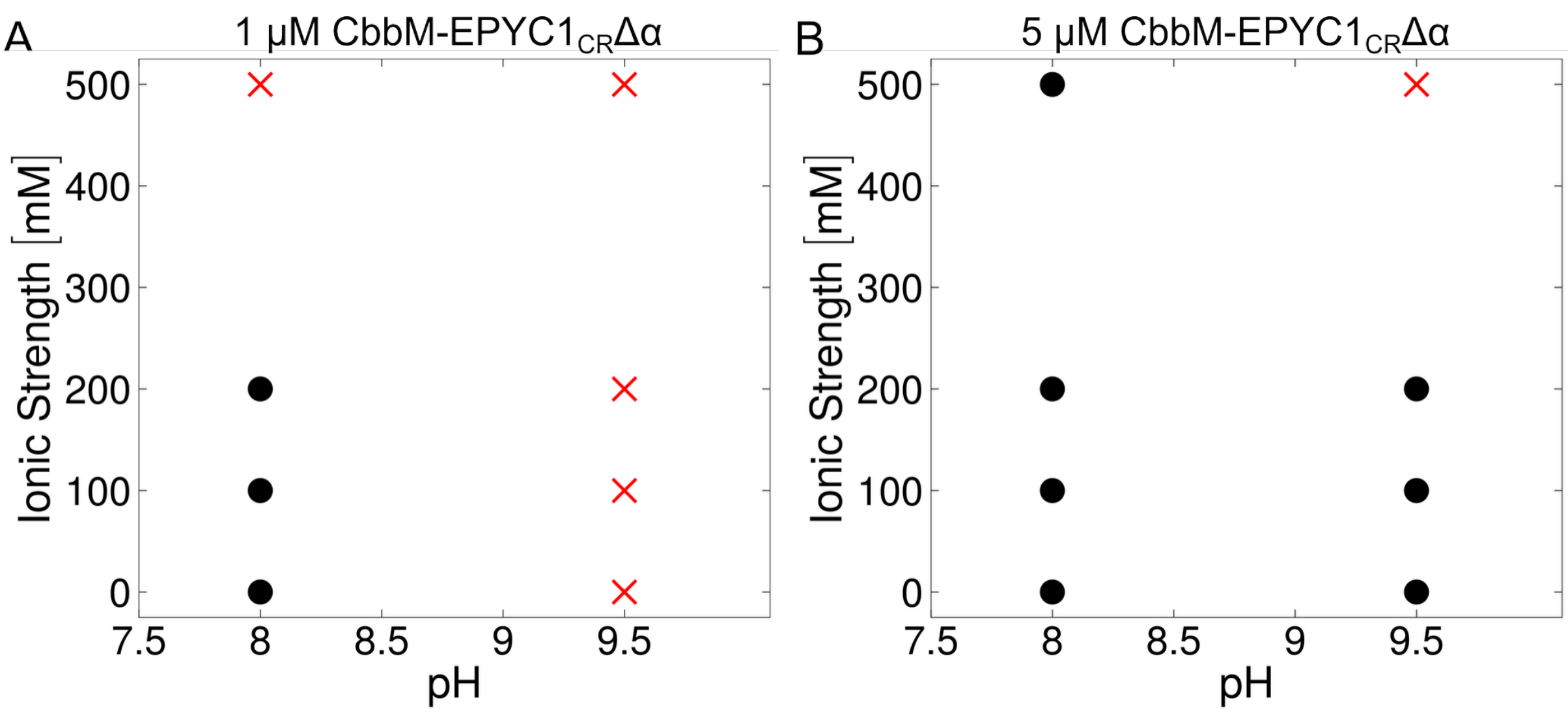

Supplementary Figure 22 Phase diagrams of CbbM-EPYC1$_{CR}$Δα. (A) Phase diagram at 50 mM Tris, 1 mM $MgCl_2$ with various salt concentrations and pH values at 1 µM protein concentration as determined by bright-field microscopy. (B) Analogous experimental setup to A with 5 µM protein. Individual points are representative from two independent replicates.

Supplementary Table 1 Constructs cloned, and tested for production, condensate formation, and catalysis. The globular Rubisco (CbbM) is marked green, while all IDPs are marked grey. N or C suffixes indicate the terminal origin from the original protein. 05 indicates the first half of the respective IDP. Δctp indicates the deletion of the chloroplast targeting peptide from the original sequence.

CbbM	DQSSRYVNLALKEEDLIAGGEHVLCAYIMKPKAGYGYVATAAHFAAESSTGTNVEVCTT<br>DDFTRGVDALVYEVDEARELTKIAYPVALFDRNITDGKAMIASFLTLTMGNNQGMGDVE<br>YAKMHDFYVPEAYRALFDGPSVNISALWKVLGRPEVDGGLVVGTIIKPKLGLRPKPFAE<br>ACHAFWLGGDFIKNDEPQGNQPFAPLRDTIALVADAMRRAQDETGEAKLFSANITADD<br>PFEIIARGEYVLETFGENASHVALLVDGYVAGAAAITTARRRFPDNFLHYHRAGHGAVTS<br>PQSKRGYTAFVHCKMARLQGASGIHTGTMGFGKMEGESSDRAIAYMLTQDEAQGPFY<br>RQSWGGMKACTPIISGGMNALRMPGFFENLGNANVILTAGGGAFGHIDGPVAGARSLR<br>QAWQAWRDGVPVLDYAREHKELARAFESFPGDADQIYPGWRKALGVEDTRSALPA
CbbM$_{L171Y}$	DQSSRYVNLALKEEDLIAGGEHVLCAYIMKPKAGYGYVATAAHFAAESSTGTNVEVCTT<br>DDFTRGVDALVYEVDEARELTKIAYPVALFDRNITDGKAMIASFLTLTMGNNQGMGDVE<br>YAKMHDFYVPEAYRALFDGPSVNISALWKVLGRPEVDGGLVVGTIIKPKLGYRPKPFAE<br>ACHAFWLGGDFIKNDEPQGNQPFAPLRDTIALVADAMRRAQDETGEAKLFSANITADD<br>PFEIIARGEYVLETFGENASHVALLVDGYVAGAAAITTARRRFPDNFLHYHRAGHGAVTS<br>PQSKRGYTAFVHCKMARLQGASGIHTGTMGFGKMEGESSDRAIAYMLTQDEAQGPFY<br>RQSWGGMKACTPIISGGMNALRMPGFFENLGNANVILTAGGGAFGHIDGPVAGARSLR<br>QAWQAWRDGVPVLDYAREHKELARAFESFPGDADQIYPGWRKALGVEDTRSALPA
CbbM -Laf1N	DQSSRYVNLALKEEDLIAGGEHVLCAYIMKPKAGYGYVATAAHFAAESSTGTNVEVCTT<br>DDFTRGVDALVYEVDEARELTKIAYPVALFDRNITDGKAMIASFLTLTMGNNQGMGDVE<br>YAKMHDFYVPEAYRALFDGPSVNISALWKVLGRPEVDGGLVVGTIIKPKLGLRPKPFAE<br>ACHAFWLGGDFIKNDEPQGNQPFAPLRDTIALVADAMRRAQDETGEAKLFSANITADD<br>PFEIIARGEYVLETFGENASHVALLVDGYVAGAAAITTARRRFPDNFLHYHRAGHGAVTS<br>PQSKRGYTAFVHCKMARLQGASGIHTGTMGFGKMEGESSDRAIAYMLTQDEAQGPFY<br>RQSWGGMKACTPIISGGMNALRMPGFFENLGNANVILTAGGGAFGHIDGPVAGARSLR<br>QAWQAWRDGVPVLDYAREHKELARAFESFPGDADQIYPGWRKALGVEDTRSALPAES<br>NQSNNGGSGNAALNRGGRYVPPHLRGGDGGAAAAASAGGDDRRGGAGGGGYRRG<br>GGNSGGGGGGGYDRGYNDNRDDRDNRGGSGGYGRDRNYEDRGYNGGGGGGGNR<br>GYNNNRGGGGGGYNRQDRGDGGSSNFSRGGYNNRDEGSDNRGSGRSYNNDRRDN<br>GGDG
Laf1N- CbbM - Laf1N	ESNQSNNGGSGNAALNRGGRYVPPHLRGGDGGAAAAASAGGDDRRGGAGGGGYRR<br>GGGNSGGGGGGGYDRGYNDNRDDRDNRGGSGGYGRDRNYEDRGYNGGGGGGGN<br>RGYNNNRGGGGGGYNRQDRGDGGSSNFSRGGYNNRDEGSDNRGSGRSYNNDRRD<br>NGGDGDQSSRYVNLALKEEDLIAGGEHVLCAYIMKPKAGYGYVATAAHFAAESSTGTN<br>VEVCTTDDFTRGVDALVYEVDEARELTKIAYPVALFDRNITDGKAMIASFLTLTMGNNQG<br>MGDVEYAKMHDFYVPEAYRALFDGPSVNISALWKVLGRPEVDGGLVVGTIIKPKLGLRP<br>KPFAEACHAFWLGGDFIKNDEPQGNQPFAPLRDTIALVADAMRRAQDETGEAKLFSANI<br>TADDPFEIIARGEYVLETFGENASHVALLVDGYVAGAAAITTARRRFPDNFLHYHRAGH<br>GAVTSPQSKRGYTAFVHCKMARLQGASGIHTGTMGFGKMEGESSDRAIAYMLTQDEA<br>QGPFYRQSWGGMKACTPIISGGMNALRMPGFFENLGNANVILTAGGGAFGHIDGPVA<br>GARSLRQAWQAWRDGVPVLDYAREHKELARAFESFPGDADQIYPGWRKALGVEDTR<br>SALPAESNQSNNGGSGNAALNRGGRYVPPHLRGGDGGAAAAASAGGDDRRGGAGG<br>GGYRRGGGNSGGGGGGGYDRGYNDNRDDRDNRGGSGGYGRDRNYEDRGYNGGG<br>GGGGNRGYNNNRGGGGGGYNRQDRGDGGSSNFSRGGYNNRDEGSDNRGSGRSY<br>NNDRRDNGGDG
CbbM - Laf1N05	DQSSRYVNLALKEEDLIAGGEHVLCAYIMKPKAGYGYVATAAHFAAESSTGTNVEVCTT<br>DDFTRGVDALVYEVDEARELTKIAYPVALFDRNITDGKAMIASFLTLTMGNNQGMGDVE<br>YAKMHDFYVPEAYRALFDGPSVNISALWKVLGRPEVDGGLVVGTIIKPKLGLRPKPFAE<br>ACHAFWLGGDFIKNDEPQGNQPFAPLRDTIALVADAMRRAQDETGEAKLFSANITADD<br>PFEIIARGEYVLETFGENASHVALLVDGYVAGAAAITTARRRFPDNFLHYHRAGHGAVTS<br>PQSKRGYTAFVHCKMARLQGASGIHTGTMGFGKMEGESSDRAIAYMLTQDEAQGPFY<br>RQSWGGMKACTPIISGGMNALRMPGFFENLGNANVILTAGGGAFGHIDGPVAGARSLR<br>QAWQAWRDGVPVLDYAREHKELARAFESFPGDADQIYPGWRKALGVEDTRSALPAES<br>NQSNNGGSGNAALNRGGRYVPPHLRGGDGGAAAAASAGGDDRRGGAGGGGYRRG<br>GGNSGGGGGGGYDRGYNDNRDDRDNRGGSGGYGRDRNYEDRGYNGGGGGGGNR<br>G
Laf1N05- CbbM - Laf1N05	GSGGYGRDRNYEDRGYNGGGGGGGNRGYNNNRGGGGGGYNRQDRGDGGSSNFS<br>RGGYNNRDEGSDNRGSGRSYNNDRRDNGGDGDQSSRYVNLALKEEDLIAGGEHVLC<br>AYIMKPKAGYGYVATAAHFAAESSTGTNVEVCTTDDFTRGVDALVYEVDEARELTKIAY<br>PVALFDRNITDGKAMIASFLTLTMGNNQGMGDVEYAKMHDFYVPEAYRALFDGPSVNIS<br>ALWKVLGRPEVDGGLVVGTIIKPKLGLRPKPFAEACHAFWLGGDFIKNDEPQGNQPFA<br>PLRDTIALVADAMRRAQDETGEAKLFSANITADDPFEIIARGEYVLETFGENASHVALLV<br>DGYVAGAAAITTARRRFPDNFLHYHRAGHGAVTSPQSKRGYTAFVHCKMARLQGASGI<br>HTGTMGFGKMEGESSDRAIAYMLTQDEAQGPFYRQSWGGMKACTPIISGGMNALRMP<br>GFFENLGNANVILTAGGGAFGHIDGPVAGARSLRQAWQAWRDGVPVLDYAREHKELA

	RAFESFPGDADQIYPGWRKALGVEDTRSALPAESNQSNNGGSGNAALNRGGRYVPPH<br>LRGGDGGAAAAASAGGDDRRGGAGGGGYRRGGGNSGGGGGGGYDRGYNDNRDD<br>RDNRG
CbbM -Ddx4N	DQSSRYVNLALKEEDLIAGGEHVLCAYIMKPKAGYGYVATAAHFAAESSTGTNVEVCTT<br>DDFTRGVDALVYEVDEARELTKIAYPVALFDRNITDGKAMIASFLTLTMGNNQGMGDVE<br>YAKMHDFYVPEAYRALFDGPSVNISALWKVLGRPEVDGGLVVGTIIKPKLGLRPKPFAE<br>ACHAFWLGGDFIKNDEPQGNQPFAPLRDTIALVADAMRRAQDETGEAKLFSANITADD<br>PFEIIARGEYVLETFGENASHVALLVDGYVAGAAAITTARRRFPDNFLHYHRAGHGAVTS<br>PQSKRGYTAFVHCKMARLQGASGIHTGTMGFGKMEGESSDRAIAYMLTQDEAQGPFY<br>RQSWGGMKACTPIISGGMNALRMPGFFENLGNANVILTAGGGAFGHIDGPVAGARSLR<br>QAWQAWRDGVPVLDYAREHKELARAFESFPGDADQIYPGWRKALGVEDTRSALPAG<br>DEDWEAEINPHMSSYVPIFEKDRYSGENGDNFNRTPASSSEMDDGPSRRDHFMKSGF<br>ASGRNFGNRDAGECNKRDNTSTMGGFGVGKSFGNRGFSNSRFEDGDSSGFWRESS<br>NDCEDNPTRNRGFSKRGGYRDGNNSEASGPYRRGGRGSFRGCRGGFGLGSPNNDL<br>DPDECMQRTGGLFGSRRPVLSGTGNGDTSQSRSGSGSERGGYKGLNEEVITGSGKN<br>SWKSEAEGGES
Ddx4N- CbbM - Ddx4N	GDEDWEAEINPHMSSYVPIFEKDRYSGENGDNFNRTPASSSEMDDGPSRRDHFMKSG<br>FASGRNFGNRDAGECNKRDNTSTMGGFGVGKSFGNRGFSNSRFEDGDSSGFWRES<br>SNDCEDNPTRNRGFSKRGGYRDGNNSEASGPYRRGGRGSFRGCRGGFGLGSPNND<br>LDPDECMQRTGGLFGSRRPVLSGTGNGDTSQSRSGSGSERGGYKGLNEEVITGSGK<br>NSWKSEAEGGESDQSSRYVNLALKEEDLIAGGEHVLCAYIMKPKAGYGYVATAAHFAA<br>ESSTGTNVEVCTTDDFTRGVDALVYEVDEARELTKIAYPVALFDRNITDGKAMIASFLTL<br>TMGNNQGMGDVEYAKMHDFYVPEAYRALFDGPSVNISALWKVLGRPEVDGGLVVGTII<br>KPKLGLRPKPFAEACHAFWLGGDFIKNDEPQGNQPFAPLRDTIALVADAMRRAQDETG<br>EAKLFSANITADDPFEIIARGEYVLETFGENASHVALLVDGYVAGAAAITTARRRFPDNFL<br>HYHRAGHGAVTSPQSKRGYTAFVHCKMARLQGASGIHTGTMGFGKMEGESSDRAIAY<br>MLTQDEAQGPFYRQSWGGMKACTPIISGGMNALRMPGFFENLGNANVILTAGGGAFG<br>HIDGPVAGARSLRQAWQAWRDGVPVLDYAREHKELARAFESFPGDADQIYPGWRKAL<br>GVEDTRSALPAGDEDWEAEINPHMSSYVPIFEKDRYSGENGDNFNRTPASSSEMDDG<br>PSRRDHFMKSGFASGRNFGNRDAGECNKRDNTSTMGGFGVGKSFGNRGFSNSRFED<br>GDSSGFWRESSNDCEDNPTRNRGFSKRGGYRDGNNSEASGPYRRGGRGSFRGCRG<br>GFGLGSPNNDLDPDECMQRTGGLFGSRRPVLSGTGNGDTSQSRSGSGSERGGYKGL<br>NEEVITGSGKNSWKSEAEGGES
CbbM -UBQLN2C	DQSSRYVNLALKEEDLIAGGEHVLCAYIMKPKAGYGYVATAAHFAAESSTGTNVEVCTT<br>DDFTRGVDALVYEVDEARELTKIAYPVALFDRNITDGKAMIASFLTLTMGNNQGMGDVEY<br>AKMHDFYVPEAYRALFDGPSVNISALWKVLGRPEVDGGLVVGTIIKPKLGLRPKPFAEAC<br>HAFWLGGDFIKNDEPQGNQPFAPLRDTIALVADAMRRAQDETGEAKLFSANITADDPFEI<br>IARGEYVLETFGENASHVALLVDGYVAGAAAITTARRRFPDNFLHYHRAGHGAVTSPQS<br>KRGYTAFVHCKMARLQGASGIHTGTMGFGKMEGESSDRAIAYMLTQDEAQGPFYRQS<br>WGGMKACTPIISGGMNALRMPGFFENLGNANVILTAGGGAFGHIDGPVAGARSLRQAW<br>QAWRDGVPVLDYAREHKELARAFESFPGDADQIYPGWRKALGVEDTRSALPAAMQAL<br>MQIQQGLQTLATEAPGLIPSFTPGVGVGVLGTAIGPVGPVTPIGPIGPIVPFTPIGPIGPIG<br>PTGPAAPPGSTGSGGPTGPTVSSAAPSETTSPTSESGPNQQFIQQMVQALAGANAPQ<br>LPNPEVRFQQQLEQLNAMGFLNREANLQALIATGGDINAAIERLLGSQPS
CbbM - UBQLN2C05	DQSSRYVNLALKEEDLIAGGEHVLCAYIMKPKAGYGYVATAAHFAAESSTGTNVEVCTT<br>DDFTRGVDALVYEVDEARELTKIAYPVALFDRNITDGKAMIASFLTLTMGNNQGMGDVEY<br>AKMHDFYVPEAYRALFDGPSVNISALWKVLGRPEVDGGLVVGTIIKPKLGLRPKPFAEAC<br>HAFWLGGDFIKNDEPQGNQPFAPLRDTIALVADAMRRAQDETGEAKLFSANITADDPFEI<br>IARGEYVLETFGENASHVALLVDGYVAGAAAITTARRRFPDNFLHYHRAGHGAVTSPQS<br>KRGYTAFVHCKMARLQGASGIHTGTMGFGKMEGESSDRAIAYMLTQDEAQGPFYRQS<br>WGGMKACTPIISGGMNALRMPGFFENLGNANVILTAGGGAFGHIDGPVAGARSLRQAW<br>QAWRDGVPVLDYAREHKELARAFESFPGDADQIYPGWRKALGVEDTRSALPAAMQAL<br>MQIQQGLQTLATEAPGLIPSFTPGVGVGVLGTAIGPVGPVTPIGPIGPIVPFTPIGPIGPIG<br>PTGPAAPPGSTGSGGPTGP
UBQLN2C-CbbM -UBQLN2C	AMQALMQIQQGLQTLATEAPGLIPSFTPGVGVGVLGTAIGPVGPVTPIGPIGPIVPFTPIG<br>PIGPIGPTGPAAPPGSTGSGGPTGPTVSSAAPSETTSPTSESGPNQQFIQQMVQALAG<br>ANAPQLPNPEVRFQQQLEQLNAMGFLNREANLQALIATGGDINAAIERLLGSQPSDQSS<br>RYVNLALKEEDLIAGGEHVLCAYIMKPKAGYGYVATAAHFAAESSTGTNVEVCTTDDFTR<br>GVDALVYEVDEARELTKIAYPVALFDRNITDGKAMIASFLTLTMGNNQGMGDVEYAKMH<br>DFYVPEAYRALFDGPSVNISALWKVLGRPEVDGGLVVGTIIKPKLGLRPKPFAEACHAFW<br>LGGDFIKNDEPQGNQPFAPLRDTIALVADAMRRAQDETGEAKLFSANITADDPFEIIARG<br>EYVLETFGENASHVALLVDGYVAGAAAITTARRRFPDNFLHYHRAGHGAVTSPQSKRGY<br>TAFVHCKMARLQGASGIHTGTMGFGKMEGESSDRAIAYMLTQDEAQGPFYRQSWGG<br>MKACTPIISGGMNALRMPGFFENLGNANVILTAGGGAFGHIDGPVAGARSLRQAWQAW<br>RDGVPVLDYAREHKELARAFESFPGDADQIYPGWRKALGVEDTRSALPAAMQALMQIQ<br>QGLQTLATEAPGLIPSFTPGVGVGVLGTAIGPVGPVTPIGPIGPIVPFTPIGPIGPIGPTGP

	AAPPGSTGSGGPTGPTVSSAAPSETTSPTSESGPNQQFIQQMVQALAGANAPQLPNP<br>EVRFQQQLEQLNAMGFLNREANLQALIATGGDINAAIERLLGSQPS
CbbM-GATA3N	DQSSRYVNLALKEEDLIAGGEHVLCAYIMKPKAGYGYVATAAHFAAESSTGTNVEVCTT<br>DDFTRGVDALVYEVDEARELTKIAYPVALFDRNITDGKAMIASFLTLTMGNNQGMGDVEY<br>AKMHDFYVPEAYRALFDGPSVNISALWKVLGRPEVDGGLVVGTIIKPKLGLRPKPFAEAC<br>HAFWLGGDFIKNDEPQGNQPFAPLRDTIALVADAMRRAQDETGEAKLFSANITADDPFEI<br>IARGEYVLETFGENASHVALLVDGYVAGAAAITTARRRFPDNFLHYHRAGHGAVTSPQS<br>KRGYTAFVHCKMARLQGASGIHTGTMGFGKMEGESSDRAIAYMLTQDEAQGPFYRQS<br>WGGMKACTPIISGGMNALRMPGFFENLGNANVILTAGGGAFGHIDGPVAGARSLRQAW<br>QAWRDGVPVLDYAREHKELARAFESFPGDADQIYPGWRKALGVEDTRSALPAVTADQP<br>RWVSHHHPAVLNGQHPDTHHPGLSHSYMDAAQYPLPEEVDVLFNIDGQGNHVPPYYG<br>NSVRATVQRYPPTHHGSQVCRPPLLHGSLPWLDGGKALGSHHTASPWNLSPFSKTSIH<br>HGSPGPLSVYPPASSSSLSGGHASPHLFTFPPTPPKDVSPDPSLSTPGSAGSARQDEK<br>ECLKYQVPLPDSMKLESSHSRGSMTALGGASSSTHHPITTYPPYVPEYSSGLFPPSSLL<br>GGSPTGFGCK
CbbM-GATA3N05	DQSSRYVNLALKEEDLIAGGEHVLCAYIMKPKAGYGYVATAAHFAAESSTGTNVEVCTT<br>DDFTRGVDALVYEVDEARELTKIAYPVALFDRNITDGKAMIASFLTLTMGNNQGMGDVE<br>YAKMHDFYVPEAYRALFDGPSVNISALWKVLGRPEVDGGLVVGTIIKPKLGLRPKPFAE<br>ACHAFWLGGDFIKNDEPQGNQPFAPLRDTIALVADAMRRAQDETGEAKLFSANITADD<br>PFEIIARGEYVLETFGENASHVALLVDGYVAGAAAITTARRRFPDNFLHYHRAGHGAVTS<br>PQSKRGYTAFVHCKMARLQGASGIHTGTMGFGKMEGESSDRAIAYMLTQDEAQGPFY<br>RQSWGGMKACTPIISGGMNALRMPGFFENLGNANVILTAGGGAFGHIDGPVAGARSLR<br>QAWQAWRDGVPVLDYAREHKELARAFESFPGDADQIYPGWRKALGVEDTRSALPAVT<br>ADQPRWVSHHHPAVLNGQHPDTHHPGLSHSYMDAAQYPLPEEVDVLFNIDGQGNHVP<br>PYYGNSVRATVQRYPPTHHGSQVCRPPLLHGSLPWLDGGKALGSHHTASPWNLSPFS<br>KTSIHHGS
GATA3N-CbbM-GATA3N	VTADQPRWVSHHHPAVLNGQHPDTHHPGLSHSYMDAAQYPLPEEVDVLFNIDGQGNH<br>VPPYYGNSVRATVQRYPPTHHGSQVCRPPLLHGSLPWLDGGKALGSHHTASPWNLSP<br>FSKTSIHHGSPGPLSVYPPASSSSLSGGHASPHLFTFPPTPPKDVSPDPSLSTPGSAGS<br>ARQDEKECLKYQVPLPDSMKLESSHSRGSMTALGGASSSTHHPITTYPPYVPEYSSGL<br>FPPSSLLGGSPTGFGCKDQSSRYVNLALKEEDLIAGGEHVLCAYIMKPKAGYGYVATAA<br>HFAAESSTGTNVEVCTTDDFTRGVDALVYEVDEARELTKIAYPVALFDRNITDGKAMIAS<br>FLTLTMGNNQGMGDVEYAKMHDFYVPEAYRALFDGPSVNISALWKVLGRPEVDGGLVV<br>GTIIKPKLGLRPKPFAEACHAFWLGGDFIKNDEPQGNQPFAPLRDTIALVADAMRRAQDE<br>TGEAKLFSANITADDPFEIIARGEYVLETFGENASHVALLVDGYVAGAAAITTARRRFPDN<br>FLHYHRAGHGAVTSPQSKRGYTAFVHCKMARLQGASGIHTGTMGFGKMEGESSDRAI<br>AYMLTQDEAQGPFYRQSWGGMKACTPIISGGMNALRMPGFFENLGNANVILTAGGGAF<br>GHIDGPVAGARSLRQAWQAWRDGVPVLDYAREHKELARAFESFPGDADQIYPGWRKA<br>LGVEDTRSALPAVTADQPRWVSHHHPAVLNGQHPDTHHPGLSHSYMDAAQYPLPEEV<br>DVLFNIDGQGNHVPPYYGNSVRATVQRYPPTHHGSQVCRPPLLHGSLPWLDGGKALG<br>SHHTASPWNLSPFSKTSIHHGSPGPLSVYPPASSSSLSGGHASPHLFTFPPTPPKDVSP<br>DPSLSTPGSAGSARQDEKECLKYQVPLPDSMKLESSHSRGSMTALGGASSSTHHPITT<br>YPPYVPEYSSGLFPPSSLLGGSPTGFGCK
CbbM-AKAP95N	DQSSRYVNLALKEEDLIAGGEHVLCAYIMKPKAGYGYVATAAHFAAESSTGTNVEVCTT<br>DDFTRGVDALVYEVDEARELTKIAYPVALFDRNITDGKAMIASFLTLTMGNNQGMGDVEY<br>AKMHDFYVPEAYRALFDGPSVNISALWKVLGRPEVDGGLVVGTIIKPKLGLRPKPFAEAC<br>HAFWLGGDFIKNDEPQGNQPFAPLRDTIALVADAMRRAQDETGEAKLFSANITADDPFEI<br>IARGEYVLETFGENASHVALLVDGYVAGAAAITTARRRFPDNFLHYHRAGHGAVTSPQS<br>KRGYTAFVHCKMARLQGASGIHTGTMGFGKMEGESSDRAIAYMLTQDEAQGPFYRQS<br>WGGMKACTPIISGGMNALRMPGFFENLGNANVILTAGGGAFGHIDGPVAGARSLRQAW<br>QAWRDGVPVLDYAREHKELARAFESFPGDADQIYPGWRKALGVEDTRSALPADMMSK<br>EGGRGGSGGGGEGIQDRESSFRFQPFESYDSRPCLPEHNPYRPSYSYDYEFDLGSDR<br>NGSFGGQYSECRDPARERGSLDGFMRGRGQGRFQDRSNPGTFMRSDPF
CbbM-AKAP95N05	DQSSRYVNLALKEEDLIAGGEHVLCAYIMKPKAGYGYVATAAHFAAESSTGTNVEVCTT<br>DDFTRGVDALVYEVDEARELTKIAYPVALFDRNITDGKAMIASFLTLTMGNNQGMGDVEY<br>AKMHDFYVPEAYRALFDGPSVNISALWKVLGRPEVDGGLVVGTIIKPKLGLRPKPFAEAC<br>HAFWLGGDFIKNDEPQGNQPFAPLRDTIALVADAMRRAQDETGEAKLFSANITADDPFEI<br>IARGEYVLETFGENASHVALLVDGYVAGAAAITTARRRFPDNFLHYHRAGHGAVTSPQS<br>KRGYTAFVHCKMARLQGASGIHTGTMGFGKMEGESSDRAIAYMLTQDEAQGPFYRQS<br>WGGMKACTPIISGGMNALRMPGFFENLGNANVILTAGGGAFGHIDGPVAGARSLRQAW<br>QAWRDGVPVLDYAREHKELARAFESFPGDADQIYPGWRKALGVEDTRSALPADMMSK<br>EGGRGGSGGGGEGIQDRESSFRFQPFESYDSRPCLPEHNPYRPSYSYDYE
AKAP95N-CbbM-AKAP95N	DMMSKEGGRGGSGGGGEGIQDRESSFRFQPFESYDSRPCLPEHNPYRPSYSYDYEF<br>DLGSDRNGSFGGQYSECRDPARERGSLDGFMRGRGQGRFQDRSNPGTFMRSDPFD<br>QSSRYVNLALKEEDLIAGGEHVLCAYIMKPKAGYGYVATAAHFAAESSTGTNVEVCTTD

	DFTRGVDALVYEVDEARELTKIAYPVALFDRNITDGKAMIASFLTLTMGNNQGMGDVEYAKMHDFYVPEAYRALFDGPSVNISALWKVLGRPEVDGGLVVGTIIKPKLGLRPKPFAEACHAFWLGGDFIKNDEPQGNQPFAPLRDTIALVADAMRRAQDETGEAKLFSANITADDPFEIIARGEYVLETFGENASHVALLVDGYVAGAAAITTARRRFPDNFLHYHRAGHGAVTSPQSKRGYTAFVHCKMARLQGASGIHTGTMGFGKMEGESSDRAIAYMLTQDEAQGPFYRQSWGGMKACTPIISGGMNALRMPGFFENLGNANVILTAGGGAFGHIDGPVAGARSLRQAWQAWRDGVPVLDYAREHKELARAFESFPGDADQIYPGWRKALGVEDTRSALPADMMSKEGGRGGSGGGGEGIQDRESSFRFQPFESYDSRPCLPEHNPYRPSYSYDYEFDLGSDRNGSFGGQYSECRDPARERGSLDGFMRGRGQGRFQDRSNPGTFMRSDPF
CbbM-FXR1C	DQSSRYVNLALKEEDLIAGGEHVLCAYIMKPKAGYGYVATAAHFAAESSTGTNVEVCTTDDFTRGVDALVYEVDEARELTKIAYPVALFDRNITDGKAMIASFLTLTMGNNQGMGDVEYAKMHDFYVPEAYRALFDGPSVNISALWKVLGRPEVDGGLVVGTIIKPKLGLRPKPFAEACHAFWLGGDFIKNDEPQGNQPFAPLRDTIALVADAMRRAQDETGEAKLFSANITADDPFEIIARGEYVLETFGENASHVALLVDGYVAGAAAITTARRRFPDNFLHYHRAGHGAVTSPQSKRGYTAFVHCKMARLQGASGIHTGTMGFGKMEGESSDRAIAYMLTQDEAQGPFYRQSWGGMKACTPIISGGMNALRMPGFFENLGNANVILTAGGGAFGHIDGPVAGARSLRQAWQAWRDGVPVLDYAREHKELARAFESFPGDADQIYPGWRKALGVEDTRSALPAQLRQIGMGFRPSSSRGTEKEKGYATDESTASSVRGSRSYSGRGRGRRGPNYTSGYGTNSELSNPSETESERKEELSDWSLAGEDERESRQQRDSRRRPGGRGRSGSAGRGRGGSRGGKSSISSVLKDPDSNPYSLLDNTESDQTADTDASESHHNTNRRRRSRRRRTDEDSSLMDGMTESDNASVNENGLDDSEQKPQRRNRSRRRRFRGQAEDRQPVTVADYISRAESQSRQRNLPKEPLAKGKKEKVKDVIEEHGPSEKVINGPRAASADKALKPQTTERNKASCQDGSKQEAILNGVS
FXR1C-CbbM-FXR1C	QLRQIGMGFRPSSSRGTEKEKGYATDESTASSVRGSRSYSGRGRGRRGPNYTSGYGTNSELSNPSETESERKEELSDWSLAGEDERESRQQRDSRRRPGGRGRSGSAGRGRGGSRGGKSSISSVLKDPDSNPYSLLDNTESDQTADTDASESHHNTNRRRRSRRRRTDEDSSLMDGMTESDNASVNENGLDDSEQKPQRRNRSRRRRFRGQAEDRQPVTVADYISRAESQSRQRNLPKEPLAKGKKEKVKDVIEEHGPSEKVINGPRAASADKALKPQTTERNKASCQDGSKQEAILNGVSDQSSRYVNLALKEEDLIAGGEHVLCAYIMKPKAGYGYVATAAHFAAESSTGTNVEVCTTDDFTRGVDALVYEVDEARELTKIAYPVALFDRNITDGKAMIASFLTLTMGNNQGMGDVEYAKMHDFYVPEAYRALFDGPSVNISALWKVLGRPEVDGGLVVGTIIKPKLGLRPKPFAEACHAFWLGGDFIKNDEPQGNQPFAPLRDTIALVADAMRRAQDETGEAKLFSANITADDPFEIIARGEYVLETFGENASHVALLVDGYVAGAAAITTARRRFPDNFLHYHRAGHGAVTSPQSKRGYTAFVHCKMARLQGASGIHTGTMGFGKMEGESSDRAIAYMLTQDEAQGPFYRQSWGGMKACTPIISGGMNALRMPGFFENLGNANVILTAGGGAFGHIDGPVAGARSLRQAWQAWRDGVPVLDYAREHKELARAFESFPGDADQIYPGWRKALGVEDTRSALPAQLRQIGMGFRPSSSRGTEKEKGYATDESTASSVRGSRSYSGRGRGRRGPNYTSGYGTNSELSNPSETESERKEELSDWSLAGEDERESRQQRDSRRRPGGRGRSGSAGRGRGGSRGGKSSISSVLKDPDSNPYSLLDNTESDQTADTDASESHHNTNRRRRSRRRRTDEDSSLMDGMTESDNASVNENGLDDSEQKPQRRNRSRRRRFRGQAEDRQPVTVADYISRAESQSRQRNLPKEPLAKGKKEKVKDVIEEHGPSEKVINGPRAASADKALKPQTTERNKASCQDGSKQEAILNGVS
CbbM-LEM2N	DQSSRYVNLALKEEDLIAGGEHVLCAYIMKPKAGYGYVATAAHFAAESSTGTNVEVCTTDDFTRGVDALVYEVDEARELTKIAYPVALFDRNITDGKAMIASFLTLTMGNNQGMGDVEYAKMHDFYVPEAYRALFDGPSVNISALWKVLGRPEVDGGLVVGTIIKPKLGLRPKPFAEACHAFWLGGDFIKNDEPQGNQPFAPLRDTIALVADAMRRAQDETGEAKLFSANITADDPFEIIARGEYVLETFGENASHVALLVDGYVAGAAAITTARRRFPDNFLHYHRAGHGAVTSPQSKRGYTAFVHCKMARLQGASGIHTGTMGFGKMEGESSDRAIAYMLTQDEAQGPFYRQSWGGMKACTPIISGGMNALRMPGFFENLGNANVILTAGGGAFGHIDGPVAGARSLRQAWQAWRDGVPVLDYAREHKELARAFESFPGDADQIYPGWRKALGVEDTRSALPARLRDEERLREEARPRGEERLREEARLREDAPLRARPAAASPRAEPWLSQPASGSAYATPGAYGDIRPSAASWVGSRGLAYPARPAQLRRRASVRGSSEEDEDARTPDRATQGPGLAARRWWAASPAPARLPSSLLGPDPRPGLRATRAGPAGAARARPEV
LEM2N-CbbM-LEM2N	RLRDEERLREEARPRGEERLREEARLREDAPLRARPAAASPRAEPWLSQPASGSAYATPGAYGDIRPSAASWVGSRGLAYPARPAQLRRRASVRGSSEEDEDARTPDRATQGPGLAARRWWAASPAPARLPSSLLGPDPRPGLRATRAGPAGAARARPEVDQSSRYVNLALKEEDLIAGGEHVLCAYIMKPKAGYGYVATAAHFAAESSTGTNVEVCTTDDFTRGVDALVYEVDEARELTKIAYPVALFDRNITDGKAMIASFLTLTMGNNQGMGDVEYAKMHDFYVPEAYRALFDGPSVNISALWKVLGRPEVDGGLVVGTIIKPKLGLRPKPFAEACHAFWLGGDFIKNDEPQGNQPFAPLRDTIALVADAMRRAQDETGEAKLFSANITADDPFEIIARGEYVLETFGENASHVALLVDGYVAGAAAITTARRRFPDNFLHYHRAGHGAVTSPQSKRGYTAFVHCKMARLQGASGIHTGTMGFGKMEGESSDRAIAYMLTQDEAQGPFYRQSWGGMKACTPIISGGMNALRMPGFFENLGNANVILTAGGGAFGHIDGPVAGARSLRQAWQAWRDGVPVLDYAREHKELARAFESFPGDADQIYPGWRKALGVEDTRSALPARLRDEERLREEARPRGEERLREEARLREDAPLRARPAAASPRAEPWLSQPASGSAYATPGAYGDIRPSAASWVGSRGLAYPARPAQLRRRASVRGSSEEDEDARTPDRATQGPGLAARRWWAASPAPARLPSSLLGPDPRPGLRATRAGPAGAARARPEV

CbbM-xCPEB4N	DQSSRYVNLALKEEDLIAGGEHVLCAYIMKPKAGYGYVATAAHFAAESSTGTNVEVCTT<br>DDFTRGVDALVYEVDEARELTKIAYPVALFDRNITDGKAMIASFLTLTMGNNQGMGDVEY<br>AKMHDFYVPEAYRALFDGPSVNISALWKVLGRPEVDGGLVVGTIIKPKLGLRPKPFAEAC<br>HAFWLGGDFIKNDEPQGNQPFAPLRDTIALVADAMRRAQDETGEAKLFSANITADDPFEI<br>IARGEYVLETFGENASHVALLVDGYVAGAAAITTARRRFPDNFLHYHRAGHGAVTSPQS<br>KRGYTAFVHCKMARLQGASGIHTGTMGFGKMEGESSDRAIAYMLTQDEAQGPFYRQS<br>WGGMKACTPIISGGMNALRMPGFFENLGNANVILTAGGGAFGHIDGPVAGARSLRQAW<br>QAWRDGVPVLDYAREHKELARAFESFPGDADQIYPGWRKALGVEDTRSALPAGDYGF<br>GVLVQSNTGNKSAFPVRFHPHLQPPHHHQNATPSPAAFINNNTAANGSSAGSAWLFPA<br>PATHNIQDEILGSEKAKSQQQEQQDPLEKQQLSPSPGQEAGILPETEKAKSEENQGDN<br>SSENGNGKEKIRIESPVLTGFDYQEATGLGTSTQPLTSSASSLTGFSNWSAAIAPSSSTII<br>NEDASFFHQGGVPAASANN
CbbM-<br>xCPEB4N05	DQSSRYVNLALKEEDLIAGGEHVLCAYIMKPKAGYGYVATAAHFAAESSTGTNVEVCTT<br>DDFTRGVDALVYEVDEARELTKIAYPVALFDRNITDGKAMIASFLTLTMGNNQGMGDVEY<br>AKMHDFYVPEAYRALFDGPSVNISALWKVLGRPEVDGGLVVGTIIKPKLGLRPKPFAEAC<br>HAFWLGGDFIKNDEPQGNQPFAPLRDTIALVADAMRRAQDETGEAKLFSANITADDPFEI<br>IARGEYVLETFGENASHVALLVDGYVAGAAAITTARRRFPDNFLHYHRAGHGAVTSPQS<br>KRGYTAFVHCKMARLQGASGIHTGTMGFGKMEGESSDRAIAYMLTQDEAQGPFYRQS<br>WGGMKACTPIISGGMNALRMPGFFENLGNANVILTAGGGAFGHIDGPVAGARSLRQAW<br>QAWRDGVPVLDYAREHKELARAFESFPGDADQIYPGWRKALGVEDTRSALPAGDYGF<br>GVLVQSNTGNKSAFPVRFHPHLQPPHHHQNATPSPAAFINNNTAANGSSAGSAWLFPA<br>PATHNIQDEILGSEKAKSQQQEQQDPLEKQQLSPSPGQ
xCPEB4N-CbbM-<br>xCPEB4N	GDYGFGVLVQSNTGNKSAFPVRFHPHLQPPHHHQNATPSPAAFINNNTAANGSSAGSA<br>WLFPAPATHNIQDEILGSEKAKSQQQEQQDPLEKQQLSPSPGQEAGILPETEKAKSEEN<br>QGDNSSENGNGKEKIRIESPVLTGFDYQEATGLGTSTQPLTSSASSLTGFSNWSAAIAP<br>SSSTIINEDASFFHQGGVPAASANNDQSSRYVNLALKEEDLIAGGEHVLCAYIMKPKAGY<br>GYVATAAHFAAESSTGTNVEVCTTDDFTRGVDALVYEVDEARELTKIAYPVALFDRNITD<br>GKAMIASFLTLTMGNNQGMGDVEYAKMHDFYVPEAYRALFDGPSVNISALWKVLGRPE<br>VDGGLVVGTIIKPKLGLRPKPFAEACHAFWLGGDFIKNDEPQGNQPFAPLRDTIALVADA<br>MRRAQDETGEAKLFSANITADDPFEIIARGEYVLETFGENASHVALLVDGYVAGAAAITTA<br>RRRFPDNFLHYHRAGHGAVTSPQSKRGYTAFVHCKMARLQGASGIHTGTMGFGKMEG<br>ESSDRAIAYMLTQDEAQGPFYRQSWGGMKACTPIISGGMNALRMPGFFENLGNANVIL<br>TAGGGAFGHIDGPVAGARSLRQAWQAWRDGVPVLDYAREHKELARAFESFPGDADQI<br>YPGWRKALGVEDTRSALPAGDYGFGVLVQSNTGNKSAFPVRFHPHLQPPHHHQNATP<br>SPAAFINNNTAANGSSAGSAWLFPAPATHNIQDEILGSEKAKSQQQEQQDPLEKQQLSP<br>SPGQEAGILPETEKAKSEENQGDNSSENGNGKEKIRIESPVLTGFDYQEATGLGTSTQP<br>LTSSASSLTGFSNWSAAIAPSSSTIINEDASFFHQGGVPAASANN
CbbM-U2AF65N	DQSSRYVNLALKEEDLIAGGEHVLCAYIMKPKAGYGYVATAAHFAAESSTGTNVEVCTT<br>DDFTRGVDALVYEVDEARELTKIAYPVALFDRNITDGKAMIASFLTLTMGNNQGMGDVEY<br>AKMHDFYVPEAYRALFDGPSVNISALWKVLGRPEVDGGLVVGTIIKPKLGLRPKPFAEAC<br>HAFWLGGDFIKNDEPQGNQPFAPLRDTIALVADAMRRAQDETGEAKLFSANITADDPFEI<br>IARGEYVLETFGENASHVALLVDGYVAGAAAITTARRRFPDNFLHYHRAGHGAVTSPQS<br>KRGYTAFVHCKMARLQGASGIHTGTMGFGKMEGESSDRAIAYMLTQDEAQGPFYRQS<br>WGGMKACTPIISGGMNALRMPGFFENLGNANVILTAGGGAFGHIDGPVAGARSLRQAW<br>QAWRDGVPVLDYAREHKELARAFESFPGDADQIYPGWRKALGVEDTRSALPASDFDEF<br>ERQLNENKQERDKENRHRKRSHSRSRSRDRKRRSRSRDRRNRDQRSASRDRRRRS<br>KPLTRGAKEEHGGLIRSPRHEKKKKVRKYWDVPPPGFEHITPMQYKAMQAAGQIPATAL<br>LPTMTPDGLAVTPTPVPVVGSQMTRQAR
CbbM-<br>U2AF65N05	DQSSRYVNLALKEEDLIAGGEHVLCAYIMKPKAGYGYVATAAHFAAESSTGTNVEVCTT<br>DDFTRGVDALVYEVDEARELTKIAYPVALFDRNITDGKAMIASFLTLTMGNNQGMGDVEY<br>AKMHDFYVPEAYRALFDGPSVNISALWKVLGRPEVDGGLVVGTIIKPKLGLRPKPFAEAC<br>HAFWLGGDFIKNDEPQGNQPFAPLRDTIALVADAMRRAQDETGEAKLFSANITADDPFEI<br>IARGEYVLETFGENASHVALLVDGYVAGAAAITTARRRFPDNFLHYHRAGHGAVTSPQS<br>KRGYTAFVHCKMARLQGASGIHTGTMGFGKMEGESSDRAIAYMLTQDEAQGPFYRQS<br>WGGMKACTPIISGGMNALRMPGFFENLGNANVILTAGGGAFGHIDGPVAGARSLRQAW<br>QAWRDGVPVLDYAREHKELARAFESFPGDADQIYPGWRKALGVEDTRSALPSDFDEF<br>ERQLNENKQERDKENRHRKRSHSRSRSRDRKRRSRSRDRRNRDQRSASRDRRRRS<br>KPLTRGAKEEHGG
U2AF65N-CbbM-<br>U2AF65N	SDFDEFERQLNENKQERDKENRHRKRSHSRSRSRDRKRRSRSRDRRNRDQRSASRD<br>RRRRSKPLTRGAKEEHGGLIRSPRHEKKKKVRKYWDVPPPGFEHITPMQYKAMQAAG<br>QIPATALLPTMTPDGLAVTPTPVPVVGSQMTRQARDQSSRYVNLALKEEDLIAGGEHVL<br>CAYIMKPKAGYGYVATAAHFAAESSTGTNVEVCTTDDFTRGVDALVYEVDEARELTKIAY<br>PVALFDRNITDGKAMIASFLTLTMGNNQGMGDVEYAKMHDFYVPEAYRALFDGPSVNIS<br>ALWKVLGRPEVDGGLVVGTIIKPKLGLRPKPFAEACHAFWLGGDFIKNDEPQGNQPFAP<br>LRDTIALVADAMRRAQDETGEAKLFSANITADDPFEIIARGEYVLETFGENASHVALLVDG<br>YVAGAAAITTARRRFPDNFLHYHRAGHGAVTSPQSKRGYTAFVHCKMARLQGASGIHT<br>GTMGFGKMEGESSDRAIAYMLTQDEAQGPFYRQSWGGMKACTPIISGGMNALRMPGF<br>FENLGNANVILTAGGGAFGHIDGPVAGARSLRQAWQAWRDGVPVLDYAREHKELARAF<br>ESFPGDADQIYPGWRKALGVEDTRSALPASDFDEFERQLNENKQERDKENRHRKRSH

	SRSRSRDRKRRSRSRDRRNRDQRSASRDRRRRSKPLTRGAKEEHGGLIRSPRHEKKK<br>KVRKYWDVPPPGFEHITPMQYKAMQAAGQIPATALLPTMTPDGLAVTPTPVPVVGSQM<br>TRQAR
CbbM-EPYC1AGΔctp	DQSSRYVNLALKEEDLIAGGEHVLCAYIMKPKAGYGYVATAAHFAAESSTGTNVEVCTT<br>DDFTRGVDALVYEVDEARELTKIAYPVALFDRNITDGKAMIASFLTLTMGNNQGMGDVEY<br>AKMHDFYVPEAYRALFDGPSVNISALWKVLGRPEVDGGLVVGTIIKPKLGLRPKPFAEAC<br>HAFWLGGDFIKNDEPQGNQPFAPLRDTIALVADAMRRAQDETGEAKLFSANITADDPFEI<br>IARGEYVLETFGENASHVALLVDGYVAGAAAITTARRRFPDNFLHYHRAGHGAVTSPQS<br>KRGYTAFVHCKMARLQGASGIHTGTMGFGKMEGESSDRAIAYMLTQDEAQGPFYRQS<br>WGGMKACTPIISGGMNALRMPGFFENLGNANVILTAGGGAFGHIDGPVAGARSLRQAW<br>QAWRDGVPVLDYAREHKELARAFESFPGDADQIYPGWRKALGVEDTRSALPAARGSW<br>RDSSSVTTKVPSRSSSSANRVSPTRSVLPANWRQELESLRNGNGSAPSAPTPAAPARS<br>SSANWRTESSSSAPKPASSAPARPSSSPRRTVTPNRSSLPPNWKQELESLRNGNGNG<br>SSSSYSSYSAPAPARSSSASWRTEAAPAAPAAAARPSSSPRKAVVPARSSLPPNWKQE<br>LESLRNGNGSSSSSASYAAPAAAPARSSSANWRTSPAPS
CbbM-EPYC1CIΔctp	DQSSRYVNLALKEEDLIAGGEHVLCAYIMKPKAGYGYVATAAHFAAESSTGTNVEVCTT<br>DDFTRGVDALVYEVDEARELTKIAYPVALFDRNITDGKAMIASFLTLTMGNNQGMGDVEY<br>AKMHDFYVPEAYRALFDGPSVNISALWKVLGRPEVDGGLVVGTIIKPKLGLRPKPFAEAC<br>HAFWLGGDFIKNDEPQGNQPFAPLRDTIALVADAMRRAQDETGEAKLFSANITADDPFEI<br>IARGEYVLETFGENASHVALLVDGYVAGAAAITTARRRFPDNFLHYHRAGHGAVTSPQS<br>KRGYTAFVHCKMARLQGASGIHTGTMGFGKMEGESSDRAIAYMLTQDEAQGPFYRQS<br>WGGMKACTPIISGGMNALRMPGFFENLGNANVILTAGGGAFGHIDGPVAGARSLRQAW<br>QAWRDGVPVLDYAREHKELARAFESFPGDADQIYPGWRKALGVEDTRSALPAARGSW<br>RDSSSTATVQASRASSATNRASPTRSVLPANWRQELETLRNGNGSSAASSAPAPARSS<br>SASWRDAAPASSAPARSSSASKKAVTPSRSALPSNWKQELESLRSSSPAPASSGSAPA<br>ARSSSASWRDAAPASSAPARSSSASKKAVTPSRSALPSNWKQELESLRSSSPAPASSG<br>SAPARSSSASWRDAAPASSAPARSSSASKKAVTPSRSALPSNWKQELESLRSNSPAPA<br>SSGSAPARSSSASWRDAPASSGSAPARSSSASWRDAPASSSSAADKAGTNPWTGKSK<br>PEIKRTPLPADWRKGL
CbbM-EPYC1CRΔctp	DQSSRYVNLALKEEDLIAGGEHVLCAYIMKPKAGYGYVATAAHFAAESSTGTNVEVCTT<br>DDFTRGVDALVYEVDEARELTKIAYPVALFDRNITDGKAMIASFLTLTMGNNQGMGDVEY<br>AKMHDFYVPEAYRALFDGPSVNISALWKVLGRPEVDGGLVVGTIIKPKLGLRPKPFAEAC<br>HAFWLGGDFIKNDEPQGNQPFAPLRDTIALVADAMRRAQDETGEAKLFSANITADDPFEI<br>IARGEYVLETFGENASHVALLVDGYVAGAAAITTARRRFPDNFLHYHRAGHGAVTSPQS<br>KRGYTAFVHCKMARLQGASGIHTGTMGFGKMEGESSDRAIAYMLTQDEAQGPFYRQS<br>WGGMKACTPIISGGMNALRMPGFFENLGNANVILTAGGGAFGHIDGPVAGARSLRQAW<br>QAWRDGVPVLDYAREHKELARAFESFPGDADQIYPGWRKALGVEDTRSALPAARGSW<br>RESSTATVQASRASSATNRVSPTRESVLPANWRQELESLRNGNGSSSAASSAPAPARS<br>SSASWRDAAPASSAPARSSASKKAVTPSRSALPSNWKQELESLRSSSPAPASSAPAPA<br>RSSSASQRDAAPASSAPARSSSSKKAVTPSRSALPSNWKQELESLRSSSPAPASSAPA<br>PARSSSASWRDAAPASSAPARSSSASKKAVTPSRSALPSNWKQELESLRSNSPAPASS<br>APAPARSSSASWRDAPASSSSSSADKAGTNPWTGKSKPEIKRTALPADWRKGL
CbbM$_{L171Y}$-EPYC1CRΔctp	DQSSRYVNLALKEEDLIAGGEHVLCAYIMKPKAGYGYVATAAHFAAESSTGTNVEVCTT<br>DDFTRGVDALVYEVDEARELTKIAYPVALFDRNITDGKAMIASFLTLTMGNNQGMGDVEY<br>AKMHDFYVPEAYRALFDGPSVNISALWKVLGRPEVDGGLVVGTIIKPKLGYRPKPFAEA<br>CHAFWLGGDFIKNDEPQGNQPFAPLRDTIALVADAMRRAQDETGEAKLFSANITADDPF<br>EIIARGEYVLETFGENASHVALLVDGYVAGAAAITTARRRFPDNFLHYHRAGHGAVTSPQ<br>SKRGYTAFVHCKMARLQGASGIHTGTMGFGKMEGESSDRAIAYMLTQDEAQGPFYRQ<br>SWGGMKACTPIISGGMNALRMPGFFENLGNANVILTAGGGAFGHIDGPVAGARSLRQA<br>WQAWRDGVPVLDYAREHKELARAFESFPGDADQIYPGWRKALGVEDTRSALPAARGS<br>WRESSTATVQASRASSATNRVSPTRESVLPANWRQELESLRNGNGSSSAASSAPAPAR<br>SSSASWRDAAPASSAPARSSASKKAVTPSRSALPSNWKQELESLRSSSPAPASSAPAP<br>ARSSSASQRDAAPASSAPARSSSSKKAVTPSRSALPSNWKQELESLRSSSPAPASSAP<br>APARSSSASWRDAAPASSAPARSSSASKKAVTPSRSALPSNWKQELESLRSNSPAPAS<br>SAPAPARSSSASWRDAPASSSSSSADKAGTNPWTGKSKPEIKRTALPADWRKGL
CbbM-EPYC1EDΔctp	DQSSRYVNLALKEEDLIAGGEHVLCAYIMKPKAGYGYVATAAHFAAESSTGTNVEVCTT<br>DDFTRGVDALVYEVDEARELTKIAYPVALFDRNITDGKAMIASFLTLTMGNNQGMGDVEY<br>AKMHDFYVPEAYRALFDGPSVNISALWKVLGRPEVDGGLVVGTIIKPKLGLRPKPFAEAC<br>HAFWLGGDFIKNDEPQGNQPFAPLRDTIALVADAMRRAQDETGEAKLFSANITADDPFEI<br>IARGEYVLETFGENASHVALLVDGYVAGAAAITTARRRFPDNFLHYHRAGHGAVTSPQS<br>KRGYTAFVHCKMARLQGASGIHTGTMGFGKMEGESSDRAIAYMLTQDEAQGPFYRQS<br>WGGMKACTPIISGGMNALRMPGFFENLGNANVILTAGGGAFGHIDGPVAGARSLRQAW<br>QAWRDGVPVLDYAREHKELARAFESFPGDADQIYPGWRKALGVEDTRSALPAARGSW<br>RDAPATSAPARPTSAGRASPTRSVLPANWKSELEKLRSSSGNGNGAASAPAPAASARP<br>TSSPKKTATPSRSSLPANWKQELESLRSSSATGSASSASPSPARSASSSWRDAPSSSS<br>SVAPARSGSAKKPVTPSRSSLPSNWKQELESLRSQSGGSSSSSSAAAAPARSASASW<br>RDAPASSSSSSAAPARSSSAKKPVTPSRSSLPSNWKQELESLRSQSGGSSSSSSAAAA<br>PARSSSASWRDAPASSSSSSSSASKAGSNPWTGKSKPEIKRTTLPADWRKSA

CbbM-<br>EPYC1GPΔctp	DQSSRYVNLALKEEDLIAGGEHVLCAYIMKPKAGYGYVATAAHFAAESSTGTNVEVCTT<br>DDFTRGVDALVYEVDEARELTKIAYPVALFDRNITDGKAMIASFLTLTMGNNQGMGDVEY<br>AKMHDFYVPEAYRALFDGPSVNISALWKVLGRPEVDGGLVVGTIIKPKLGLRPKPFAEAC<br>HAFWLGGDFIKNDEPQGNQPFAPLRDTIALVADAMRRAQDETGEAKLFSANITADDPFEI<br>IARGEYVLETFGENASHVALLVDGYVAGAAAITTARRRFPDNFLHYHRAGHGAVTSPQS<br>KRGYTAFVHCKMARLQGASGIHTGTMGFGKMEGESSDRAIAYMLTQDEAQGPFYRQS<br>WGGMKACTPIISGGMNALRMPGFFENLGNANVILTAGGGAFGHIDGPVAGARSLRQAW<br>QAWRDGVPVLDYAREHKELARAFESFPGDADQIYPGWRKALGVEDTRSALPAARGSW<br>RESSTVTATPAGRSSSAANRVSPTRSVLPANWRQELESLRNGNGNGSSAAAAPAPAPA<br>RSASASWRDAPAAAAPARPSSSPKKAVTPSRSSLPANWKQELEALRGGSSSSSASWR<br>TESAPAAAPARSGSKKAVTPSRSSLPANWKQELESMRSASPAPSSAPAAPARSSSASW<br>RSESGSSSSSAAADKAGTNPWTGKAKVEIKRTALPADWRKGL
CbbM-<br>EPYC1TSΔctp	DQSSRYVNLALKEEDLIAGGEHVLCAYIMKPKAGYGYVATAAHFAAESSTGTNVEVCTT<br>DDFTRGVDALVYEVDEARELTKIAYPVALFDRNITDGKAMIASFLTLTMGNNQGMGDVEY<br>AKMHDFYVPEAYRALFDGPSVNISALWKVLGRPEVDGGLVVGTIIKPKLGLRPKPFAEAC<br>HAFWLGGDFIKNDEPQGNQPFAPLRDTIALVADAMRRAQDETGEAKLFSANITADDPFEI<br>IARGEYVLETFGENASHVALLVDGYVAGAAAITTARRRFPDNFLHYHRAGHGAVTSPQS<br>KRGYTAFVHCKMARLQGASGIHTGTMGFGKMEGESSDRAIAYMLTQDEAQGPFYRQS<br>WGGMKACTPIISGGMNALRMPGFFENLGNANVILTAGGGAFGHIDGPVAGARSLRQAW<br>QAWRDGVPVLDYAREHKELARAFESFPGDADQIYPGWRKALGVEDTRSALPAAKGSW<br>RDAPTVTAQPGRAASSAKPTSPTRSVLPANWRQELESLRGGNGNGAAAAPAAAAPRA<br>QSAGWRDAPASAPAASAPMKKTATPARTALPANWKQELESLRSSSTGGASAAPAAAPA<br>RASSASWRDAPAAAPASKSSSPAPAGTNPWTGKSKIEIKRTALPADWRKGL
CbbM-<br>EPYC1VAΔctp	DQSSRYVNLALKEEDLIAGGEHVLCAYIMKPKAGYGYVATAAHFAAESSTGTNVEVCTT<br>DDFTRGVDALVYEVDEARELTKIAYPVALFDRNITDGKAMIASFLTLTMGNNQGMGDVEY<br>AKMHDFYVPEAYRALFDGPSVNISALWKVLGRPEVDGGLVVGTIIKPKLGLRPKPFAEAC<br>HAFWLGGDFIKNDEPQGNQPFAPLRDTIALVADAMRRAQDETGEAKLFSANITADDPFEI<br>IARGEYVLETFGENASHVALLVDGYVAGAAAITTARRRFPDNFLHYHRAGHGAVTSPQS<br>KRGYTAFVHCKMARLQGASGIHTGTMGFGKMEGESSDRAIAYMLTQDEAQGPFYRQS<br>WGGMKACTPIISGGMNALRMPGFFENLGNANVILTAGGGAFGHIDGPVAGARSLRQAW<br>QAWRDGVPVLDYAREHKELARAFESFPGDADQIYPGWRKALGVEDTRSALPAARGSW<br>RESSTVTAQPAGRSSSASARVSPTRSVLPANWRQELESLRNGNGSSSAAAPAPAPAPA<br>RSASASWRTEPAPAAASAPSRSPKKAVTPTRSSLPANWKQELESLRGGSSSSSSAPAP<br>AAASAPSRSPKKAVTPTRSSLPANWKQELESLRGGSSSSSSAPAPAAASAPSRSPKKA<br>VTPTRSSLPANWKQELESLRGSSSPAPAEAAPARSSSASWRTESPAPAKESSASKAGT<br>NPWTGKSKIEIKRTALPSNWRSGL
CbbM-<br>EPYC1VCΔctp	DQSSRYVNLALKEEDLIAGGEHVLCAYIMKPKAGYGYVATAAHFAAESSTGTNVEVCTT<br>DDFTRGVDALVYEVDEARELTKIAYPVALFDRNITDGKAMIASFLTLTMGNNQGMGDVEY<br>AKMHDFYVPEAYRALFDGPSVNISALWKVLGRPEVDGGLVVGTIIKPKLGLRPKPFAEAC<br>HAFWLGGDFIKNDEPQGNQPFAPLRDTIALVADAMRRAQDETGEAKLFSANITADDPFEI<br>IARGEYVLETFGENASHVALLVDGYVAGAAAITTARRRFPDNFLHYHRAGHGAVTSPQS<br>KRGYTAFVHCKMARLQGASGIHTGTMGFGKMEGESSDRAIAYMLTQDEAQGPFYRQS<br>WGGMKACTPIISGGMNALRMPGFFENLGNANVILTAGGGAFGHIDGPVAGARSLRQAW<br>QAWRDGVPVLDYAREHKELARAFESFPGDADQIYPGWRKALGVEDTRSALPAARGSW<br>RESATVTAQPAGRASSSNRVSPTRSVLPANWRQELESLRNGNGNGAAAAPAPAPAPAR<br>SSSASWRSESSAAPAAASTPSRSTKKPVTPTRTSLPANWKQELESLRGSSSSSPAAAA<br>PAPARSSSSPKKAVTPTRSSLPANWKQELESLRGGSSSAASAPAAAAAPAAASAPSRSP<br>KKAVTPTRSSLPANWKQELESLRGGSSSSSSAPAPAAAPAPARSSSASWRTESPAPAN<br>ESSSAAAKAGTNPWTGKAKIEIKRTTLPADWRRQL
CbbM-<br>EPYC1ΔαΔctp	DQSSRYVNLALKEEDLIAGGEHVLCAYIMKPKAGYGYVATAAHFAAESSTGTNVEVCTT<br>DDFTRGVDALVYEVDEARELTKIAYPVALFDRNITDGKAMIASFLTLTMGNNQGMGDVEY<br>AKMHDFYVPEAYRALFDGPSVNISALWKVLGRPEVDGGLVVGTIIKPKLGLRPKPFAEAC<br>HAFWLGGDFIKNDEPQGNQPFAPLRDTIALVADAMRRAQDETGEAKLFSANITADDPFEI<br>IARGEYVLETFGENASHVALLVDGYVAGAAAITTARRRFPDNFLHYHRAGHGAVTSPQS<br>KRGYTAFVHCKMARLQGASGIHTGTMGFGKMEGESSDRAIAYMLTQDEAQGPFYRQS<br>WGGMKACTPIISGGMNALRMPGFFENLGNANVILTAGGGAFGHIDGPVAGARSLRQAW<br>QAWRDGVPVLDYAREHKELARAFESFPGDADQIYPGWRKALGVEDTRSALPAARGSW<br>RESSTATVQASRASSATNRVSPTRESVLPANGNGSSSAASSAPAPARSSSASWRDAAP<br>ASSAPARSSASKKAVTPSRSALPSNSSPAPASSAPAPARSSSASQRDAAPASSAPARSS<br>SSKKAVTPSRSALPSNSSPAPASSAPAPARSSSASWRDAAPASSAPARSSSASKKAVTP<br>SRSALPSNWKQELESLRSNSPAPASSAPAPARSSSASWRDAPASSSSSSADKAGTNP<br>WTGKSKPEIKRTALPAD
CbbM-<br>EPYC1ΔFRΔctp	DQSSRYVNLALKEEDLIAGGEHVLCAYIMKPKAGYGYVATAAHFAAESSTGTNVEVCTT<br>DDFTRGVDALVYEVDEARELTKIAYPVALFDRNITDGKAMIASFLTLTMGNNQGMGDVEY<br>AKMHDFYVPEAYRALFDGPSVNISALWKVLGRPEVDGGLVVGTIIKPKLGLRPKPFAEAC<br>HAFWLGGDFIKNDEPQGNQPFAPLRDTIALVADAMRRAQDETGEAKLFSANITADDPFEI<br>IARGEYVLETFGENASHVALLVDGYVAGAAAITTARRRFPDNFLHYHRAGHGAVTSPQS<br>KRGYTAFVHCKMARLQGASGIHTGTMGFGKMEGESSDRAIAYMLTQDEAQGPFYRQS<br>WGGMKACTPIISGGMNALRMPGFFENLGNANVILTAGGGAFGHIDGPVAGARSLRQAW

	QAWRDGVPVLDYAREHKELARAFESFPGDADQIYPGWRKALGVEDTRSALPAARGSW<br>RESSTATVQASRASSAGNGSSSAASSAPAPARSSSASWRDAAPASSAPASSPAPASSA<br>PAPARSSSASQRDAAPASSAPASSPAPASSAPAPARSSSASWRDAAPASSAPANSPAPA<br>SSAPAPARSSSASWRDAPASSSSSSADKAGTNPWTGKSKPEIKRTALPADWRKGL
CbbM-<br>EPYC1Anc1Δctp	DQSSRYVNLALKEEDLIAGGEHVLCAYIMKPKAGYGYVATAAHFAAESSTGTNVEVCTT<br>DDFTRGVDALVYEVDEARELTKIAYPVALFDRNITDGKAMIASFLTLTMGNNQGMGDVEY<br>AKMHDFYVPEAYRALFDGPSVNISALWKVLGRPEVDGGLVVGTIIKPKLGLRPKPFAEAC<br>HAFWLGGDFIKNDEPQGNQPFAPLRDTIALVADAMRRAQDETGEAKLFSANITADDPFEI<br>IARGEYVLETFGENASHVALLVDGYVAGAAAITTARRRFPDNFLHYHRAGHGAVTSPQS<br>KRGYTAFVHCKMARLQGASGIHTGTMGFGKMEGESSDRAIAYMLTQDEAQGPFYRQS<br>WGGMKACTPIISGGMNALRMPGFFENLGNANVILTAGGGAFGHIDGPVAGARSLRQAW<br>QAWRDGVPVLDYAREHKELARAFESFPGDADQIYPGWRKALGVEDTRSALPAARGSW<br>RESSTATVQASRSASAANRVSPTRSVLPANWRQELESLRNGNGSSSSASSAPAPARSS<br>SASWRDAPASSAPARSSSASKKAVTPSRSSLPANWKQELESLRSSSPAPASSSAAPAR<br>SSSASWRDAPASSAPARSSSASKKPVTPSRSSLPSNWKQELESLRSSSPAPASSSAAP<br>ARSSSASWRDAPASSAPARSSSASKKAVTPSRSSLPSNWKQELESLRSSSPAPASSSA<br>APARSSSASWRDAPASSSSSSADKAGTNPWTGKSKVEIKRTTLP
CbbM-<br>EPYC1Anc2Δctp	DQSSRYVNLALKEEDLIAGGEHVLCAYIMKPKAGYGYVATAAHFAAESSTGTNVEVCTT<br>DDFTRGVDALVYEVDEARELTKIAYPVALFDRNITDGKAMIASFLTLTMGNNQGMGDVEY<br>AKMHDFYVPEAYRALFDGPSVNISALWKVLGRPEVDGGLVVGTIIKPKLGLRPKPFAEAC<br>HAFWLGGDFIKNDEPQGNQPFAPLRDTIALVADAMRRAQDETGEAKLFSANITADDPFEI<br>IARGEYVLETFGENASHVALLVDGYVAGAAAITTARRRFPDNFLHYHRAGHGAVTSPQS<br>KRGYTAFVHCKMARLQGASGIHTGTMGFGKMEGESSDRAIAYMLTQDEAQGPFYRQS<br>WGGMKACTPIISGGMNALRMPGFFENLGNANVILTAGGGAFGHIDGPVAGARSLRQAW<br>QAWRDGVPVLDYAREHKELARAFESFPGDADQIYPGWRKALGVEDTRSALPAARGSW<br>RDSSTVTATQPGRSSSAANRVSPTRSVLPANWRQELESLRNGNGNGSSAASAPAPAR<br>SSSASWRDAPAPAAPARPSSSPKKTVTPSRSSLPANWKQELESLRSSSSGSASSSAPS<br>ARSSSSSWRDPPASSAPARSRSASKKPVTPSRSSLPSNWKQELESLRGGSPAPSSSA<br>AAPARSASASWRDPAASSAPARSSSASKKAVTPSRSSLPSNWKQELESLRSSSPAPAS<br>AAAAPARSSSASWRDAPASSSSSSADKAGTNPWTGKSKIEIKRTTLP
CbbM-<br>EPYC1Anc3Δctp	DQSSRYVNLALKEEDLIAGGEHVLCAYIMKPKAGYGYVATAAHFAAESSTGTNVEVCTT<br>DDFTRGVDALVYEVDEARELTKIAYPVALFDRNITDGKAMIASFLTLTMGNNQGMGDVEY<br>AKMHDFYVPEAYRALFDGPSVNISALWKVLGRPEVDGGLVVGTIIKPKLGLRPKPFAEAC<br>HAFWLGGDFIKNDEPQGNQPFAPLRDTIALVADAMRRAQDETGEAKLFSANITADDPFEI<br>IARGEYVLETFGENASHVALLVDGYVAGAAAITTARRRFPDNFLHYHRAGHGAVTSPQS<br>KRGYTAFVHCKMARLQGASGIHTGTMGFGKMEGESSDRAIAYMLTQDEAQGPFYRQS<br>WGGMKACTPIISGGMNALRMPGFFENLGNANVILTAGGGAFGHIDGPVAGARSLRQAW<br>QAWRDGVPVLDYAREHKELARAFESFPGDADQIYPGWRKALGVEDTRSALPAARGSW<br>RESSTATVQASRASSATNRVSPTRSVLPANWRQELESLRNGNGSSSAASSAPAPARSS<br>SASWRDAAPASSAPARSSSASKKAVTPSRSALPSNWKQELESLRSSSPAPASSASAPA<br>RSSSASWRDAAPASSAPARSSSASKKAVTPSRSALPSNWKQELESLRSSSPAPASSAS<br>APARSSSASWRDAAPASSAPARSSSASKKAVTPSRSALPSNWKQELESLRSNSPAPAS<br>SASAPARSSSASWRDAPASSSSSSADKAGTNPWTGKSKPEIKRTALP
CbbM-<br>EPYC1Anc4Δctp	DQSSRYVNLALKEEDLIAGGEHVLCAYIMKPKAGYGYVATAAHFAAESSTGTNVEVCTT<br>DDFTRGVDALVYEVDEARELTKIAYPVALFDRNITDGKAMIASFLTLTMGNNQGMGDVEY<br>AKMHDFYVPEAYRALFDGPSVNISALWKVLGRPEVDGGLVVGTIIKPKLGLRPKPFAEAC<br>HAFWLGGDFIKNDEPQGNQPFAPLRDTIALVADAMRRAQDETGEAKLFSANITADDPFEI<br>IARGEYVLETFGENASHVALLVDGYVAGAAAITTARRRFPDNFLHYHRAGHGAVTSPQS<br>KRGYTAFVHCKMARLQGASGIHTGTMGFGKMEGESSDRAIAYMLTQDEAQGPFYRQS<br>WGGMKACTPIISGGMNALRMPGFFENLGNANVILTAGGGAFGHIDGPVAGARSLRQAW<br>QAWRDGVPVLDYAREHKELARAFESFPGDADQIYPGWRKALGVEDTRSALPAARGSW<br>RDAPTVTAQPGRSSSAANRVSPTRSVLPANWRQELESLRNGNGNGSSAASAPAPARS<br>SSASWRDAPAAPARPSSSPKKTATPSRSSLPANWKQELESLRSSSTGSASSAAPSARS<br>SSSSWRDPSSSSSVAPARSGSKKPVTPSRSSLPSNWKQELESLRGGSSSSSSAAAAP<br>ARSASASWRDPAASSAPARSSSSKKAVTPSRSSLPSNWKQELESLRSSSPAPASAAAA<br>PARSSSASWRDAPASSSSSSASKAGTNPWTGKSKIEIKRTTLP
CbbM-<br>EPYC1Anc5Δctp	DQSSRYVNLALKEEDLIAGGEHVLCAYIMKPKAGYGYVATAAHFAAESSTGTNVEVCTT<br>DDFTRGVDALVYEVDEARELTKIAYPVALFDRNITDGKAMIASFLTLTMGNNQGMGDVEY<br>AKMHDFYVPEAYRALFDGPSVNISALWKVLGRPEVDGGLVVGTIIKPKLGLRPKPFAEAC<br>HAFWLGGDFIKNDEPQGNQPFAPLRDTIALVADAMRRAQDETGEAKLFSANITADDPFEI<br>IARGEYVLETFGENASHVALLVDGYVAGAAAITTARRRFPDNFLHYHRAGHGAVTSPQS<br>KRGYTAFVHCKMARLQGASGIHTGTMGFGKMEGESSDRAIAYMLTQDEAQGPFYRQS<br>WGGMKACTPIISGGMNALRMPGFFENLGNANVILTAGGGAFGHIDGPVAGARSLRQAW<br>QAWRDGVPVLDYAREHKELARAFESFPGDADQIYPGWRKALGVEDTRSALPAARGSW<br>RDSSTVTATPAGRSSSAANRVSPTRSVLPANWRQELESLRNGNGNGSSAASAPAPARS<br>SSASWRDAPAPAAPARPSSSPKKAVTPSRSSLPANWKQELESLRSGSSGSSSSSAPAA<br>RSSSSKKPVTPSRSSLPANWKQELESLRGGSSSSASAPARSASASWRDPAAASAPARS<br>SSGSKKAVTPSRSSLPANWKQELESLRSSSPAPASAAAAPARSSSASWRDSPASSSSS<br>SADKAGTNPWTGKSKIEIKRTTLP

CbbM-EPYC1Anc6Δctp	DQSSRYVNLALKEEDLIAGGEHVLCAYIMKPKAGYGYVATAAHFAAESSTGTNVEVCTT<br>DDFTRGVDALVYEVDEARELTKIAYPVALFDRNITDGKAMIASFLTLTMGNNQGMGDVEY<br>AKMHDFYVPEAYRALFDGPSVNISALWKVLGRPEVDGGLVVGTIIKPKLGLRPKPFAEAC<br>HAFWLGGDFIKNDEPQGNQPFAPLRDTIALVADAMRRAQDETGEAKLFSANITADDPFEI<br>IARGEYVLETFGENASHVALLVDGYVAGAAAITTARRRFPDNFLHYHRAGHGAVTSPQS<br>KRGYTAFVHCKMARLQGASGIHTGTMGFGKMEGESSDRAIAYMLTQDEAQGPFYRQS<br>WGGMKACTPIISGGMNALRMPGFFENLGNANVILTAGGGAFGHIDGPVAGARSLRQAW<br>QAWRDGVPVLDYAREHKELARAFESFPGDADQIYPGWRKALGVEDTRSALPAARGSW<br>RESSTVTATPAGRSSSAANRVSPTRSVLPANWRQELESLRNGNGNGSSAASAPAPAPA<br>RSASASWRDAPAPAAPARPSSSPKKAVTPSRSSLPANWKQELESLRGGSSSSSSSAAP<br>AARSSSSKKPVTPTRSSLPANWKQELESLRGGSSSSASAPAPAAASAPARSGSKKAVT<br>PSRSSLPANWKQELESLRSSSPAPASAPAAPARSSSASWRSESPASSSSSSADKAGTN<br>PWTGKAKIEIKRTTLP
CbbM-EPYC1Anc7Δctp	DQSSRYVNLALKEEDLIAGGEHVLCAYIMKPKAGYGYVATAAHFAAESSTGTNVEVCTT<br>DDFTRGVDALVYEVDEARELTKIAYPVALFDRNITDGKAMIASFLTLTMGNNQGMGDVEY<br>AKMHDFYVPEAYRALFDGPSVNISALWKVLGRPEVDGGLVVGTIIKPKLGLRPKPFAEAC<br>HAFWLGGDFIKNDEPQGNQPFAPLRDTIALVADAMRRAQDETGEAKLFSANITADDPFEI<br>IARGEYVLETFGENASHVALLVDGYVAGAAAITTARRRFPDNFLHYHRAGHGAVTSPQS<br>KRGYTAFVHCKMARLQGASGIHTGTMGFGKMEGESSDRAIAYMLTQDEAQGPFYRQS<br>WGGMKACTPIISGGMNALRMPGFFENLGNANVILTAGGGAFGHIDGPVAGARSLRQAW<br>QAWRDGVPVLDYAREHKELARAFESFPGDADQIYPGWRKALGVEDTRSALPAARGSW<br>RESSTVTAQPAGRSSSASNRVSPTRSVLPANWRQELESLRNGNGNGSAAAPAPAPAPA<br>RSSSASWRESPAPAAASTPSRSPKKAVTPTRSSLPANWKQELESLRGGSSSSSSAAAP<br>APARSSSSKKAVTPTRSSLPANWKQELESLRGGSSSSASAPAPAAASAPSRSPKKAVTP<br>TRSSLPANWKQELESLRGSSSPAPASAPAPARSSSASWRTESPAPANESSASKAGTNP<br>WTGKAKIEIKRTTLP
EPYC1CRΔctp	ARGSWRESSTATVQASRASSATNRVSPTRESVLPANWRQELESLRNGNGSSSAASSA<br>PAPARSSSASWRDAAPASSAPARSSASKKAVTPSRSALPSNWKQELESLRSSSPAPAS<br>SAPAPARSSSASQRDAAPASSAPARSSSSKKAVTPSRSALPSNWKQELESLRSSSPAPA<br>SSAPAPARSSSASWRDAAPASSAPARSSSASKKAVTPSRSALPSNWKQELESLRSNSP<br>APASSAPAPARSSSASWRDAPASSSSSSADKAGTNPWTGKSKPEIKRTALPADWRKGL
EPYC1ΔαΔctp	ARGSWRESSTATVQASRASSATNRVSPTRESVLPANGNGSSSAASSAPAPARSSSAS<br>WRDAAPASSAPARSSASKKAVTPSRSALPSNSSPAPASSAPAPARSSSASQRDAAPAS<br>SAPARSSSSKKAVTPSRSALPSNSSPAPASSAPAPARSSSASWRDAAPASSAPARSSSA<br>SKKAVTPSRSALPSNWKQELESLRSNSPAPASSAPAPARSSSASWRDAPASSSSSSAD<br>KAGTNPWTGKSKPEIKRTALPAD

Supplementary Table 2 Parameter estimates from mass action-mass transfer model fit to observed data. Models were fitted to 0.3 mM and 1 mM RuBP aerobic time curve data. For both situations (varying partitioning indicated by the partition coefficient K(x) or the Michaelis Menten constants K_M(x)), substrate concentrations, active site content and phase volume were varied as well to attain good model fit. For K_M variation only the K_Ms inside the condensates were considered. Enzyme partitioning was assumed at 91%, condensate radius at average 10 µm, diffusion coefficient of substrates at 1e-11 µm^2/s, as measured for other small molecules previously[50].

	Mass action-mass transfer partitioning			Catalytic parameters inside the condensates		
	$K(CO_2)$	$K(O_2)$	K(RuBP)	$K_M(CO_2)$	$K_M(O_2)$	K_M(RuBP)
0.3 mM RuBP	170.7	47.4	1.2	17.7	4031.9	780.9
1 mM RuBP	96.5	11.3	0.4	66.5	767.5	806.8

Supplementary Table 3 Percentage Identity of ancestors inferred via mtInv versus JJT. The percentage identity of sequences inferred with two different models to test for the ASR robustness is shown.

Protein	Percentage Identity [%]
Anc1	100
Anc2	99.03
Anc3	99.04
Anc4	99.34
Anc5	97.91
Anc6	98.56
Anc7	98.56

Supplementary Table 4 Percentage Identity of natural variants and ancestors with respect to the EPYC1$_{CR}$ from *C. reinhardtii*.

Protein	Percentage Identity [%]
Chlamydomonas reinhardtii	100
Chlamydomonas schloesseri	82.82
Chlamydomonas incerta	83.01
Volvox africanus	57.82
Volvox carteri	56.00
Edaphochlamys debaryana	55.05
Gonium pectoral	56.28
Astrephomene gubernaculifera	58.82
Tetrabaena socialis	56.98
Anc1	84.10
Anc2	80.70
Anc3	97.54
Anc4	76.49
Anc5	68.31
Anc6	58.19
Anc7	57.38

Supplementary Table 5 Parameter settings for the Agilent 6470 mass spectrometer.

Name	Precurser Ion	Product Ion	Collision energy [V]	Fragmentor Voltage [V]	Cell Accelorator Voltage [V]	Dwell time [msec]	Polarity
RuBP	309.09	97	17	140	5	90	Negative
RuBP	309.09	79	59	140	5	90	Negative
Ru5P	229	97	8	140	5	90	Negative
Ru5P	229	79	48	140	5	90	Negative
3-PG	185.06	85.06	0	140	5	90	Negative
3-PG	185.06	97	13	140	5	90	Negative
3-PG	185.06	79	32	140	5	90	Negative
2-PG	155	97	19	140	5	90	Negative
2-PG	155	79	33	140	5	90	Negative

Supplementary Table 6 X-ray data collection and structure refinement statistics.

Sample	Inactive form of CbbM	Activated form of CbbM
Accession	PDB 9I26	PDB 9I27
Ligands	-	Mg^{2+}, CABP
**Data collection**		
Beamline	PETRA III - P13	PETRA III - P13
Wavelength (Å)	0.9762	0.9762
Space Group	$P\,4_1\,2_1\,2$	$P\,3_2\,2\,1$
Unit cell dimensions		
a, b, c (Å)	78.98, 78.98, 277.23	133.80, 133.80, 151.57
α, β, γ (°)	90.00, 90.00, 90.00	90.00, 90.00, 90.00
Resolution (Å)	29.45 - 2.30 (2.36 - 2.30)	29.59 - 2.30 (2.36 - 2.30)
Unique reflections	40094 (2889)	69625 (5111)
Multiplicity	26.38 (27.62)	11.51 (12.02)
Completeness (%)	99.8 (100.0)	99.4 (99.9)
I / σI	24.95 (3.53)	12.28 (3.10)
R_{merge}	0.131 (1.389)	0.192 (1.255)
$CC_{1/2}$	1.000 (0.656)	0.996 (0.805)
**Refinement**		
R_{work} / R_{free}	0.2036 / 0.2263	0.1978 / 0.2166
RMS bonds	0.002	0.003
RMS angles	0.442	0.650
Ramachandran		
favored (%)	96.91	97.68
allowed (%)	2.84	2.32
outliers (%)	0.25	0.00
Rotamer outliers (%)	0.00	0.00
Number of atoms	6524	7459
Protein	6369	7000
Ligands	-	415
Solvent	155	44
Average B-factor	50.90	31.15
Protein	51.05	31.04
Ligands	-	26.56
Solvent	44.87	33.46

Values in parentheses correspond to the highest resolution shell.

Supplementary Table 7 CryoEM data collection, refinement, and model statistics.

**Data collection**	
Model	CbbM-EPYC1$_{CR}$Δα
Accessions	PDB 9I28, EMD-52579
Microscope	Titan Krios G3i
Voltage (kV)	300
Camera	K3
Magnification	135,000
Pixel size at detector (Å/pixel)	0.655
Total electron exposure (e^-/Å^2)	60
Frames per exposure	60
Defocus range (μm)	-1.2 to -2.4
Automation software	EPU, CryoSPARC
Micrographs collected (no.)	11525
Micrographs used (no.)	7116
Total extracted particles (no.)	2167614
Final particles (no.)	209691
Point-group	*C* 2
Resolution (global, Å)	2.9
FSC$_{0.143}$	3.3 / 2.9 (unmasked / masked)
Resolution range (local, Å)	2.5 – 6.0 (according to local resolution histogram)
Map sharpening B-factor (Å^2)	-108.7
Map sharpening methods	Phenix anisotropic sharpening
**Model Refinement**	
Refinement package	Phenix
method	real space
resolution cutoff	2.89
Protein residues	898 (2×modified lysine - KCX)
Ligands	2× Mg^{2+}, 2× CABP
Model-Map scores	
CC$_{Volume}$	0.81
avg. FSC$_{0.143}$	2.93 / 2.89 (unmasked / masked)
B-factors (Å^2) (min / max / mean)	
Protein residues	16.13 / 113.46 / 53.69
Ligands	47.45 / 91.06 / 71.05
R.M.S. Bond lengths (Å)	0.004
R.M.S. Bond angles (°)	0.617
**Validation**	
MolProbity score	1.57
CaBLAM outliers	1.91
Clashscore	6.28
Poor rotamers (%)	0.44
C-beta deviations	0.00
Ramachandran favored (%)	96.51
Ramachandran outliers (%)	0.00

Supplementary Table 8: Reaction mixtures for the fluorescence detection of Rubisco carboxylation activity.

40 x Mix	Volumes	Stock concentrations	Final concentrations
Kpi buffer	82.3 μL	pH 7.5, 50 mM	
Amplex	25 μL	20 mM	2.5 mM
Pgmi	37.7 μL	265 μM	50 μM
HRP	2 μL	1000 U/mL	10 U/mL
ADP	20 μL	500 mM	50 mM
Enolase	10 μL	5 mg/mL	0.25 mg/mL
Carbonic anhydrase	10 μL	2 mg/mL	0.1 mg/mL
Pyruvate kinase	10 μL	25 U/mL	1.25 U/mL

V(total)	200 uL

Reaction mix	Volumes	Stock concentrations	Final concentrations
Rubisco	x		1 µM
40 x Mix	1 µL		
$MgCl_2$	0.5 µL	0.2 M	2.5 mM
$NaHCO_3$	0.5 µL	500 mM	6.25 mM
Pyruvate oxidase	1 µL	1 U/mL	0.025 U/mL
1-5-Ribulose bisphosphate	0.4 µL	100 mM	1 mM
Tris buffer	Rest	pH 7.5, 50 mM	
V(total)	40 µL		
